# Supplementary material for: Effects of PROtein enriched MEDiterranean Diet and EXercise on nutritional status and cognition in adults at risk of undernutrition and cognitive decline: the PROMED-EX Randomised Controlled Trial
Source: BMJ Open. 2023 Oct 26;13(10):e070689. doi: 10.1136/bmjopen-2022-070689 (PMC10603411; doi:10.1136/bmjopen-2022-070689)
Supplement: Supplementary data [file bmjopen-2022-070689supp002.pdf]

Appendix 2- PROMED-EX Trial Steering Committee (TSC)

Table S1: TSC members and their roles

Member	Role
**Prof Emma Stevenson**	Independent Chair
**Prof Jayne Woodside**	Dependant- scientific advisor
**Prof Dorothee Volkert**	Dependant- scientific advisor
**Prof Lisette de Groot**	Dependant- scientific advisor
**Prof Lorraine Brennan**	Dependant- scientific advisor
**Prof Chris Cardwell**	Dependant- statistician
**Ms Bernie Devlin**	Independent lay member
**Ms Eleanor Butterwick**	Independent lay member
**Ms Clare Jess**	Facilitator
**Dr Roisin O'Neill/ Dr Dominic Farsi**	Dependant- Trial manager
**Dr Claire McEvoy**	Dependant- PROMED-EX principal investigator

Table S2: Roles and responsibilities of the TSC

**Roles and responsibilities**	
Aims of the TSC	To act as the oversight body for PROMED-EX on behalf of the Sponsor/Funder.
Terms of reference	The role of the TSC is to provide oversight for the trial [Medical Research Council (MRC) Good Clinical Practice (GCP) guidelines (1998)]. It should also provide advice through its independent Chairperson to the PI, local trial team, BBSRC on all aspects of the trial.
Specific roles of TSC	• provide expert oversight of the trial<br>• maintain confidentiality of all trial information that is not already in the public domain<br>• make decisions as to the future continuation (or otherwise) of the trial/s<br>• monitor recruitment rates and encourage the trial team to develop strategies to deal with any recruitment problems<br>• review regular reports of the trial from the trial team.<br>• assess the impact and relevance of any accumulating external evidence<br>• monitor completion of CRFs and comment on strategies from trial team to encourage satisfactory completion in the future<br>• monitor follow-up rates and review strategies from Trial team to deal with problems<br>• approve any amendments to the protocol, where appropriate<br>• approve any proposals by the Trial team concerning any change to the design of the trial, including additional substudies<br>• oversee the timely reporting of trial results<br>• comment on the main trial manuscript<br>• comment on any abstracts and presentations of any results *during* the running of the trial
