# Supplementary material for: Effects of PROtein enriched MEDiterranean Diet and EXercise on nutritional status and cognition in adults at risk of undernutrition and cognitive decline: the PROMED-EX Randomised Controlled Trial
Source: BMJ Open. 2023 Oct 26;13(10):e070689. doi: 10.1136/bmjopen-2022-070689 (PMC10603411; doi:10.1136/bmjopen-2022-070689)
Supplement: Supplementary data [file bmjopen-2022-070689supp003.pdf]

Study no ___________ Date___________

PROMED DIET SCORE

Visit: Baseline ☐ 3 mon ☐ 6 mon ☐

Questions	Self-reported	Criteria for 1 point	Point?
1. Do you use olive oil or rapeseed oil as your main cooking fat?		Yes	
2. How much olive/ rapeseed oil do you consume in a given day? Including oil used for frying, salads, out-of-house meals etc (in tablespoons)<br><br>How much olive or rapeseed oil-based spread do you consume in a given day? (in teaspoons)		≥1 tbsp oil / day<br><br>**and/or**<br><br>3 tsp spread/ day	
3. How many servings of dairy foods (milk, cheese, yogurts) or plant proteins alternatives do you eat per day?		≥2 portions per day	
4. How many portions of fruit (including natural fruit juices) do you consume per day? (1 portion = 1 apple/banana (80g), small glass juice (150ml)		≥2 portions/ day	
5. How many vegetable servings do you consume per day? Including raw/ cooked vegetables, salad but not including potatoes (1 serving: 3 tablespoons/80g)		≥3 portions/ day	
6. How many servings of legumes (peas, beans and lentils including kidney beans, baked beans, chickpeas, red lentils etc) do you consume per week? (1 serving :3 tablespoons/ 80g)		≥3 servings/ week	
7. How many servings of red meat including beef, pork, lamb and minced beef do you consume per week? (1 serving: medium portion/ 100–150 g)		≤2 servings /week	
8. How many servings of processed meat including ham, bacon, sausages, meat pies and other meat products etc.) do you consume per week? (1 serving: medium portion/ 100–150 g)		≤1 serving /week	
9. How many servings of chicken/ turkey do you consume per week? (1 serving: medium portion/ 100-150g)		≥2 servings /week	
10. How many servings of fish (tuna, cod, haddock, salmon, mackerel, herring, and sardines etc, including tinned varieties, excluding crumbed or battered fish) or shellfish do you consume per week? (1 serving: 1 fillet/small fish or 140g)		≥2 servings /week	
11. Do you preferentially consume wholegrain bread and/ or cereal and/ or rice and/ or pasta instead of non-wholegrain (white) varieties?		Yes	
12. How many servings of natural nuts do you consume per week? (1 serving: 1 small handful/ 30 g)		≥3 servings /week	
13. How many times per week do you consume sweet foods (including biscuits, buns, pastries, chocolate, sweets, sweet or carbonated beverages or desserts)?		≤3 times/ week	
14. How often would you consume up to 3 small glasses of wine or equivalent other alcoholic beverages per week? (1 small glass:125ml)		1-3 glasses or equivalent ≥ 3 days/ week	
