# Supplementary material for: Effects of PROtein enriched MEDiterranean Diet and EXercise on nutritional status and cognition in adults at risk of undernutrition and cognitive decline: the PROMED-EX Randomised Controlled Trial
Source: BMJ Open. 2023 Oct 26;13(10):e070689. doi: 10.1136/bmjopen-2022-070689 (PMC10603411; doi:10.1136/bmjopen-2022-070689)
Supplement: Supplementary data [file bmjopen-2022-070689supp004.pdf]

PROMED-EX STUDY ID:_______________

CONSENT FORM

Title of Project:	**PROMED-EX**: A Randomised Controlled Trial to evaluate the effect of a protein enriched Mediterranean Diet (MedDiet) and exercise intervention on the nutritional status and cognitive performance of individuals at risk of undernutrition and cognitive decline.
**Chief Investigator:**	Dr Claire McEvoy
**Study Number:**	21/NW/0215

Please initial each box

1. I confirm that I have read, or had read to me, and understand the participant information sheet dated 20/11/21, version 3 for the above study. I have had the opportunity to ask questions and these have been answered fully. ☐

2. I understand that my participation is voluntary and I am free to withdraw at any time, without giving any reason and without my legal rights or medical care being affected. ☐

3. I understand that data collected as part of this study may be looked at by authorised individuals from Queen's University Belfast, the research team or regulatory authorities where it is relevant to my taking part in this research. I give permission for these individuals to have access to this information. ☐

4. I understand the study is being conducted by researchers from Queen's University Belfast and that my personal information will be held securely on University premises and handled in accordance with the Data Protection Act 2018 and General Data Protection Regulations. ☐

Ward NA, *et al. BMJ Open* 2023; 13:e070689. doi: 10.1136/bmjopen-2022-070689

PROMED-EX STUDY ID:________________

5. I agree to the research team informing my General Practitioner (GP) if my blood pressure is higher than normal (≥179/109) or tests completed during the study (specifically the nutritional assessment or the cognitive test scores) suggest I may be malnourished or have a cognitive impairment that may require medical intervention. ☐

6. I understand that the information I provide may be published as part of a report. Confidentiality and anonymity will be maintained and it will not be possible to identify me from any publications. ☐

7. I agree to gift a small amount of the blood that will be transferred from Queen's University Belfast to our collaborators laboratory at University College Dublin for analysis. Samples will be labelled using a unique study ID and they will not contain any identifiable information. ☐

8. I understand that if I withdraw (or lose capacity to consent) during the trial, I agree that data and samples already taken can be retained, but no further samples will be collected. ☐

9. I agree to take part in the above study inclusive of all the procedures mentioned in the participant information sheet. ☐

Optional measurement

10. I agree to gift a small sample of stool at the start and end of the 6 month study. This will be sent for analysis to study collaborators at the Quadram Institute, Norwich, England. Samples will be labelled with a unique study ID and they will not contain any identifiable information. ☐

Regarding future Research Studies (Optional)

11. I give permission for my stored, anonymised blood sample to be analysed in future research studies for indicators of age-related health and nutrition–related factors, or by other research groups. ☐

PROMED_Consent Form V3. 20.11.2021

PROMED-EX STUDY ID:________________

Regarding future research studies (Cont.)

12. I agree to being contacted and invited to take part in future studies of a similar nature. I understand that I am only agreeing to receive information and I am under no obligation to take part in any future studies. ☐

*If you decide not to consent to being contacted in the future it will not have any influence on your involvement in this particular research study and will not affect any standard of care that you receive.

________________________	______________________	________________
Name of Participant (please print)	Signature	Date

___________________________	______________________	_________________
Name of Person Taking Consent (Please print)	Signature	Date

Contact details for study team

Chief Investigator	Trial Manager
Dr Claire McEvoy<br>Centre for Public Health<br>01.024 Institute of Clinical Sciences, A<br>Queen's University Belfast<br>Grosvenor Road<br>Belfast<br>BT12 6BJ<br>Email: c.mcevoy@qub.ac.uk<br><br>Telephone: 0044 2897 6078	Dr Dominic Farsi<br>Centre for Public Health<br>02.031 Institute of Clinical Sciences, B<br>Queen's University Belfast<br>Grosvenor Road<br>Belfast<br>BT12 6BJ<br>Email: d.farsi@qub.ac.uk<br><br>PROMED-EX Mobile: 07594 966740

PROMED_Consent Form V3. 20.11.2021
