# Supplementary material for: Effects of PROtein enriched MEDiterranean Diet and EXercise on nutritional status and cognition in adults at risk of undernutrition and cognitive decline: the PROMED-EX Randomised Controlled Trial
Source: BMJ Open. 2023 Oct 26;13(10):e070689. doi: 10.1136/bmjopen-2022-070689 (PMC10603411; doi:10.1136/bmjopen-2022-070689)
Supplement: Author's manuscript [file bmjopen-2022-070689.draft_revisions.pdf]

BMJ Open

BMJ Open is committed to open peer review. As part of this commitment we make the peer review history of every article we publish publicly available.

When an article is published we post the peer reviewers' comments and the authors' responses online. We also post the versions of the paper that were used during peer review. These are the versions that the peer review comments apply to.

The versions of the paper that follow are the versions that were submitted during the peer review process. They are not the versions of record or the final published versions. They should not be cited or distributed as the published version of this manuscript.

BMJ Open is an open access journal and the full, final, typeset and author-corrected version of record of the manuscript is available on our site with no access controls, subscription charges or pay-per-view fees (http://bmjopen.bmj.com).

If you have any questions on BMJ Open's open peer review process please email info.bmjopen@bmj.com

Study Protocol: Randomised controlled trial to evaluate the effects of a PROtein enriched MEDiterranean Diet and EXercise on nutritional status and cognition in undernourished adults with subjective cognitive decline: The PROMED-EX Study.

Journal:	*BMJ Open*
Manuscript ID	bmjopen-2022-070689
Article Type:	Protocol
Date Submitted by the Author:	05-Dec-2022

Complete List of Authors:	Ward, Nicola Ann; Queen's University Belfast, Centre for Public Health<br>Reid-McCann, Rachel; Queen's University Belfast, Centre for Public Health<br>Brennan, Lorraine; University College Dublin School of Agriculture and Food Science, Institute of Food and Health and Conway Institute<br>Cardwell, Christopher; Queen's University Belfast, Centre for Public Health<br>de Groot , LC; Wageningen University, Division of Human Nutrition<br>Maggi, Stefania; Neuroscience Research Institute National Research Council Padua Branch, Aging Branch<br>McCaffrey, Noel; c/o Irish Wheelchair Association, ExWell Medical<br>McGuinness, Bernadette; Queen's University Belfast, Centre for Public Health<br>McKinley, Michelle C.; Queen's University Belfast, Centre for Public Health<br>Noale, M; Neuroscience Research Institute National Research Council Padua Branch, Aging Branch<br>O'Neill, Roisin; Queen's University Belfast, Centre for Public Health<br>Prinelli, Federica; Institute for Biomedical Technologies National Research Council, Epidemiology Unit<br>Sergi, Giuseppe; University of Ferrara, Department of Medical Sciences<br>Trevisan, Caterina.; University of Ferrara Department of Medical Sciences, Department of Medical Sciences<br>Volkert, Dorothee; Friedrich-Alexander-Universitat Erlangen-Nurnberg, Institute for Biomedicine of Aging<br>Woodside, Jayne; Queen's University Belfast, Centre for Public Health<br>McEvoy, Claire T.; Queen's University Belfast, Centre for Public Health; Global Brain Health Institute, Trinity College Dublin, Ireland & University of California San Francisco, USA

Keywords:	Nutrition < TROPICAL MEDICINE, Dementia < NEUROLOGY, NUTRITION & DIETETICS, PUBLIC HEALTH, PREVENTIVE MEDICINE

For peer review only - http://bmjopen.bmj.com/site/about/guidelines.xhtml

Study Protocol: Randomised controlled trial to evaluate the effects of a PROtein enriched MEDiterranean Diet and EXercise on nutritional status and cognition in undernourished adults with subjective cognitive decline: The PROMED-EX Study.

Ward N[1], Reid-McCann R[1], Brennan L[2], Cardwell C[1], de Groot CPGM[3], Maggi S[4], McCaffrey N[5], McGuinness B[1], McKinley MC[1], Noale M[4], O'Neill RF[1], Prinelli F[6], Sergi G[7], Trevisan C[7], Volkert D[8], Woodside JV[1], McEvoy CT[1,9] and the PROMED-COG consortium.

[1]Centre for Public Health, Queen's University Belfast, Belfast. Northern Ireland. UK.

[2]School of Agriculture and Food Science, Institute of Food and Health and Conway Institute, University College Dublin, Dublin. Ireland.

[3]Division of Human Nutrition, Wageningen University, Wageningen. Netherlands

[4] Neuroscience Institute, Aging Branch, National Research Council (CNR), Padua. Italy.

[5]ExWell Medical, c/o Irish Wheelchair Association, Dublin. Ireland.

[6] Institute of Biomedical Technologies, Epidemiology Unit, National Research Council (CNR), Segrate (MI). Italy

[7]Department of Medical Sciences, University of Ferrara, Ferrara. Italy

[8]Institute for Biomedicine of Aging, Friedrich-Alexander-Universität Erlangen-Nürnberg, Nuremberg. Germany.

[9]The Global Brain Institute, Trinity College Dublin, Ireland & University of California San Francisco, USA.

Corresponding Author: Dr Claire McEvoy, Centre for Public Health, Institute of Clinical Sciences building A, Queen's University Belfast, Grosvenor Road, Belfast. BT12 6BJ. Northern Ireland. UK. Email: **c.mcevoy@qub.ac.uk**

Word count, excluding title page, abstract, references, figures, and table

Key Words: nutrition, exercise, cognitive function, dementia

For peer review only

ABSTRACT

Introduction Undernutrition leading to unplanned weight loss is common in older age and has been linked to increased dementia risk in later life. Weight loss can precede dementia by a decade or more, providing a unique opportunity for early intervention to correct undernutrition and potentially prevent or delay cognitive impairment. The combined effects of diet and exercise on undernutrition have not yet been evaluated. The objective of the PROMED-EX trial is to determine the effect of a protein-enriched Mediterranean Diet, with and without exercise, on nutritional status and cognitive performance in older adults at risk of undernutrition and cognitive decline.

Methods One hundred and five participants aged 60 years and over at risk of undernutrition and with subjective cognitive decline will be recruited to participate in a 6-month, single-blind, parallel-group randomised controlled trial. Participants will be block randomised into one of three groups: Group 1. PROMED-EX (diet + exercise), Group 2. PROMED (diet only), and Group 3. Standard Care (control). The primary outcome is nutritional status measured using the Mini Nutritional Assessment (MNA). Secondary outcomes include cognitive function, nutritional intake, body composition, physical function, and quality of life. Mechanistic pathways for potential diet and exercise-induced change in nutritional status and cognition will be explored by measuring inflammatory, metabolic, nutritional and metabolomic biomarkers.

Ethics and dissemination The study is approved by the UK Office for Research Ethics Committee (Ref 21/NW/0215). Written informed consent will be obtained from participants prior to recruitment. Research results will be disseminated to the public via meetings and media and the scientific community through conference presentations and publication in academic journals.

Trial registration NCT 05166564.

Strengths and limitations of this study

- PROMED-EX is the first randomised controlled trial designed to evaluate the effect of an optimised protein-enriched Mediterranean diet, with and without exercise, on nutritional status and cognitive performance in a high-risk population.
- The integration of state-of-the-art metabolomics, metabolic, inflammatory, and nutritional biomarkers will help elucidate biological mechanisms underlying how diet can influence nutrition status and cognition and how exercise can interact with nutrition to modify these processes.
- A Personal and Public Involvement (PPI) group will contribute at all stages of the PROMED-EX trial to optimise the research design and dissemination strategy of the research findings.
- The nature of the 'real world' pragmatic study design means that it is not possible to blind participants to treatment allocation, possibly leading to performance bias.
- Data will be analysed by a blinded member of the study team to minimise detection bias.

For peer review only - http://bmjopen.bmj.com/site/about/guidelines.xhtml

INTRODUCTION

Undernutrition, defined as insufficient food and/or nutrient intake that results in altered body composition and unplanned weight loss,[1] is common in older age and affects >1.3 million older adults in the UK.[2] Individuals with cognitive impairment are particularly vulnerable to undernutrition and its many adverse consequences. Undernourished people with dementia experience more rapid decline in cognitive and physical functions, increased neuropsychiatric symptoms, and greater risk of hospitalisation and mortality, than those with adequate nutritional status.[3,4] Being undernourished has also emerged as a potentially modifiable risk factor for dementia.[5] In prospective studies, weight loss increases dementia risk by 30-40%[6] and precedes the onset of dementia by a decade or more,[7,8] making it an intervention target that should be prioritised.

Causes of undernutrition are increased nutrient requirements, inadequate dietary intake and reduced nutrient bioavailability,[9] but the exact mechanisms of undernutrition in dementia are not clear. It is possible that weight loss may be both a consequence and cause of neurodegeneration and dementia. Loss of muscle and weight in older adults has been linked to accelerated decline in brain structures,[10-12] even after exclusion of cognitive impairment,[12] suggesting a pathophysiological role of undernutrition in dementia. In contrast, weight loss could be an early marker of dementia development. Pathological changes in the brain are present 10-15 years prior to the clinical symptoms of cognitive impairment and induce inflammatory[13] and physiological changes beyond normal ageing that can have a negative impact on nutrition and body weight. Dementia pathology affects brain regions such as the hypothalamus which is important for appetite regulation and eating behaviour.[14] This may explain alterations of leptin and ghrelin metabolic signals that are observed in early disease.[15] Early brain changes can also affect chemosensory perception including taste and olfactory function,[16] as well as satiety regulation, protein and lipid synthesis,[17] and food preferences. Disease progression and worsening cognitive impairment can cause forgetfulness to eat, dysphagia,[18] sleep disturbance[19] and social withdrawal,[20] which can all have adverse effects on food intake.[21]

To date, most studies have focused on the impact of diet on physical function,[22] with limited number of interventions targeting undernutrition for cognitive benefit. Short-term treatment of undernutrition has been shown to improve both nutritional status and cognitive performance.[23-25] However, small sample sizes as well as differences in study populations and nutritional interventions (dietetic counselling,[23] oral supplementation,[24,25] meal provision[25]), and cognitive outcomes make it difficult to know whether addressing undernutrition could protect against cognitive decline. Hence, trials are warranted to test the efficacy of intervention for undernutrition in populations at increased risk of cognitive decline but not yet cognitively impaired and may benefit from early intervention.

The Mediterranean Diet (MedDiet) is a nutrient dense dietary pattern with proven anti-inflammatory benefits[26] and may offer protection against cognitive decline during ageing. Observational data have reported links between the MedDiet and favourable changes in brain structures and functions,[27] slower rate of cognitive decline[28,29] and decreased dementia risk, [30,31] which appear to be attributable to the combined effect of different foods rather than the action of a single food or nutrient.[32] Furthermore, the MedDiet is associated with nutritional adequacy and lower risk of undernutrition and micronutrient deficiency.[33] Adequate protein (1.0-1.2/kg body weight/day)[34] and energy intake[35] are critical for preventing muscle loss[36] and undernutrition in older adults.[37] However, emerging data suggest that a high-quality dietary pattern is also important,[38] lending support to a 'whole diet' approach for preventing muscle loss and functional decline during ageing.

For peer review only - http://bmjopen.bmj.com/site/about/guidelines.xhtml

Physical exercise has positive effects on cognition,[39] especially in domains of executive function and processing speed,[40] by increasing neurogenesis and upregulating neurotrophic factors.[41] However, it is not known how diet and exercise interact for neuroprotection. Multi-domain interventions that include both a healthy diet and exercise appear to elicit stronger positive results for cognitive performance than each intervention in isolation.[42] Furthermore, exercise can stimulate protein synthesis[43] and enhance muscle mass and functional performance in older adults,[44] making it an attractive target for preventing undernutrition. An optimal protein MedDiet may induce additive effects on nutritional status and cognition when combined with exercise, but this has not yet been evaluated.

The PROtein enriched MEDiterranean Diet and EXercise (PROMED-EX) randomised controlled trial (RCT) aims to determine the effects of a protein-enriched MedDiet, with and without exercise, in comparison to standard care, on nutritional status in adults at risk of undernutrition and cognitive decline. PROMED-EX is part of the European "PROMED-COG" project, funded under the Horizon Joint Programming Initiative ‘a healthy diet for a healthy life’ to establish evidence for the balance between diet and physical activity for preventing undernutrition and promoting healthy neurocognitive ageing.[45]

METHODS AND ANALYSIS

PROMED-EX is a 6-month, single-blind, parallel-group RCT aiming to recruit 105 eligible adults residing in Northern Ireland (NI), aged 60 years+ and at risk of both undernutrition and cognitive decline. The protocol has been written in accordance with the Standard Protocol Items: Recommendations for Interventional Trials (SPIRIT) guidelines,[46] and a summary is reported using the checklist (Appendix 1). An independent Trial Steering Committee (TSC) has been established (Appendix 2) to oversee the conduct of the study.

Patient and public involvement (PPI)

A patient and public involvement (PPI) group (n=8) comprising older adults residing in NI with lived experience of subjective cognitive decline (SCD) and/or poor nutritional status were recruited for PROMED-EX. PPI members have contributed to the intervention design and co-development of materials, including lay summary, participant information sheet and consent form, advertisement materials, written educational resources for the interventions. Besides the trial design, two PPI members sit on the TSC and will input to future stages including implementation, analysis, and dissemination.

Study Design

Study participants will be randomised into one of three groups: Group 1. PROMED-EX: Protein-enriched MedDiet and exercise intervention (n=35); Group 2. PROMED: Protein-enriched MedDiet intervention (n=35); or Group 3. Control: Standard care healthy eating diet sheet (n=35) as shown in Figure 1. Participants will be informed of their allocated group after baseline data has been collected. Those randomised to the PROMED-EX and PROMED interventions will receive active support to adopt the behavioural targets in the first 3 months, after which time they will continue with self-directed maintenance of the newly adopted behaviours for a further 3 months.

For peer review only - http://bmjopen.bmj.com/site/about/guidelines.xhtml

Recruitment and Eligibility of Study Participants

Multiple recruitment strategies will be employed including paid Facebook advertisements, other media (e.g., newspaper, radio, and television), and identification of potentially eligible participants from community networks, primary care sectors and health and social care trusts in NI. Those interested in participating will be provided with an information sheet detailing the study protocol, followed by a phone call to discuss study procedures.

Potentially eligible participants will be screened by telephone/video to last around 45 minutes. Individuals will be eligible to enrol if they are community-dwelling, aged ≥60 years, at risk of undernutrition defined by a Mini Nutritional Assessment -Short Form (MNA-SF) score of 8-11[47] and have a positive screen for SCD.[48] The screening assessment includes the Telephone cognitive screen (T-CogS) or Mini Mental State Examination (MMSE) to exclude participants with cognitive impairment and a health and medical history questionnaire to determine if the participant has any medical conditions that compromise their ability to take part. This includes if they receive nutritional support, have a diagnosis of mild cognitive impairment or dementia, or dietary restrictions as shown in Table 1.

Randomisation, blinding and allocation

A study statistician independent of recruitment, intervention delivery and data collection will perform block randomisation using a computer-generated random number sequence in blocks of size 6 (using command RALLOC in STATA Release 16, StataCorp LP, College Station). Allocations will be prepared in sealed opaque envelopes by an independent study administrator and will not be revealed to participants until completion of baseline data collection. It will not be possible to blind the participant nor study researchers involved in intervention delivery to the allocated intervention group; however, data collection and analysis will be carried out by investigators blinded to treatment allocation.

Table 1: PROMED-EX Trial eligibility criteria

Inclusion Criteria	Exclusion Criteria
• Age ≥ 60 years<br>• Community-dwelling<br>• Mini Nutrition Assessment-Short Form (MNA-SF) score 8-11, indicating increased risk of undernutrition[47]<br><br>• Positive screen for subjective cognitive decline (SCD)[48] defined as a 'yes' response to the question:	• Cognitive impairment: Telephone cognitive screen (T-CogS) ≤*21* or Mini Mental State Examination (MMSE) ≤*24* or clinical diagnosis of mild cognitive impairment or dementia<br>• In receipt of prescribed oral or artificial nutritional support<br>• Dietary restrictions/allergies to PROMED foods (e.g. nuts) or taking high-dose nutritional (e.g. Vitamin

For peer review only

"During the past 12 months, have you experienced confusion or slight memory loss that is happening more often or is getting worse?"<br><br>• Ability to provide informed consent.	E) that limit the ability to take part in the study<br>• Living in long-term care facilities<br>• Self-reported:<br>o Dysphagia<br>o Chronic Kidney disease<br>o Diabetes complications or HbA1c >8%<br>o Significant medical comorbidities that may alter cognitive test performance, e.g., stroke, brain injury<br>• Inability to complete cognitive testing due to language or visual impairment

Interventions

Group 1. PROMED-EX

Protein enriched MedDiet intervention

Participants will meet with a trained researcher within one week following randomisation to receive personalised dietary counselling based on their baseline anthropometry, MNA score and PROMED diet score (appendix 3). This session will last up to 60 minutes where education will be provided on adequate nutritional status, avoiding weight loss, and encouraging regular meal pattern. Participants will be encouraged to set dietary goals to adopt PROMED diet behaviours (shown in Box 1) to achieve overall intervention targets: (1) increased protein intake up to 1.5g per kg current body weight;[37] (2) increased MNA score by ≥2 points; and (3) increased PROMED diet score by ≥2 points. Daily energy requirements will be calculated at 30kcal/kg of body weight with a supplement of 400-600kcal only if weight gain is indicated.[49]

Box 1: PROMED dietary behaviours

- 1tbsp of olive oil per day
- 5 or more portions of fruit and vegetables per day (≥ 2 portions fruit and ≥3 portions vegetables per day)
- Preferential consumption of chicken and oily fish, ≥2 portions of each per week (and reduction of red meat <2).
- ≥2 portions per day of wholegrain foods (including wholemeal bread, wholegrain rice, whole wheat pasta)
- 3 handfuls of natural nuts (90g) per week
- ≥2 portions per day of dairy foods (including, milk, cheese, yoghurts) or plant protein alternatives
- Moderate alcohol (1-2 glasses of wine/beer per day consumed with meals) for alcohol consumers

For peer review only - http://bmjopen.bmj.com/site/about/guidelines.xhtml

Written materials (PROMED diet guide and recipe book) will be provided to support adoption of diet behaviours. Participants will be given a recipe book with suggested quick, low-cost menu plans and practical dietary tips to overcome small appetite. Key foods will be delivered to their home free of charge for the first 3-months to encourage adoption of the PROMED diet behaviours. Foods will be self-selected by participants from a pre-defined list to facilitate personal preference and will include olive oil, fruits and vegetables, wholegrain foods (cereals, breads, rice and pasta), nuts, nut butters, canned oily fish and low-fat dairy foods.

Exercise intervention

The exercise intervention has been adapted from a commercially available 'ExWell@home' group exercise rehabilitation programme that is acceptable to older adults and suggested to improve health outcomes in those with chronic disease.[50] The home-based exercise intervention comprises strength, aerobic, core stability and balance exercises available across four levels of ability: (1) chair based, (2) basic, (3) standard and (4) advanced. After the dietary education session, participants will receive a personal exercise prescription involving a written plan, demonstration of exercises suitable for their fitness level, and instruction on safety aspects to minimise injury risk. Baseline fitness level will be determined using measured blood pressure, pulse rate, short physical performance test results, handgrip strength test obtained from the baseline study visit, and their assessed performance in a 'basic level' class with the trained researcher.

Participants will be asked to report their perceived intensity of exertion using the Borg Rate of Perceived Exertion (RPE) scale (6-20),[51] aiming for "somewhat hard" within a range of 12-14 points. Participants will be instructed to increase or decrease the frequency and intensity of exercise over the first 4 weeks to meet the intervention target of two x 30–60-minute moderate intensity exercise sessions per week. Participants will receive additional supportive materials to achieve their exercise goals including pre-recorded exercise classes, a written home-based exercise resource, a pedometer, and exercise diary.

Group 2. PROMED

Participants will receive only the Protein enriched MedDiet intervention and will be advised to keep other lifestyle factors unchanged throughout the study period, including maintenance of their usual physical activity level.

Group 3. Standard care

Participants will receive a general healthy eating diet sheet provided at baseline. To encourage retention in the study, personalised dietary advice and written materials will be offered to control group participants upon completion of the final study assessment.

Maximising and monitoring compliance with the diet and exercise interventions

For study participants randomised to the PROMED-EX and PROMED interventions (Groups 1 and 2 respectively) several approaches will be used to facilitate behaviour change and encourage compliance with the diet and exercise goals. The initial education session will be scheduled at a convenient time for the participant and in a convenient location (either in a dedicated research facility, the participants own home or via remote video call). Partners or caregivers who are responsible for food preparation and/or shopping will also be invited to the education session if applicable. Furthermore, techniques demonstrated to support diet and

For peer review only - http://bmjopen.bmj.com/site/about/guidelines.xhtml

exercise behaviour change have been incorporated into the intervention written materials and include ‘instruction on how to perform behaviour’, ‘action planning’, ‘self-monitoring’ and ‘problem solving.’[52]

Participants will be encouraged to self-monitor their compliance in achieving the diet and exercise goals where applicable. The researcher will contact participants weekly via telephone during the first 3-months to review individual progress, monitor compliance and body weight, deal with any difficulties participants may be experiencing in meeting their goals and provide support and personalised feedback as required. Participants will also be able to contact the researcher at any time during the 6-month intervention period.

Data collection and Study outcomes

Participants will meet with a researcher at baseline and after 3- and 6-months to complete a study assessment lasting no longer than 2.5-hours. The assessment will take place in a dedicated research facility at Queen’s University Belfast (QUB) or in the participant’s home if preferred. A summary of the outcomes and data collection measures at study time-points are found in Table 2.

Table 2: Overview of study data collection variables and measures

Study outcomes	Data collection variables/ measures	Baseline	3-month	6-month
**Primary outcome**				
Nutritional status	Mini Nutrition Assessment (MNA®) long-form questionnaire[53]	□	□	□
**Secondary outcomes**				
Nutrition and body composition				
Nutritional intake	PROMED diet score questionnaire and 4-day food diary	□	□	□
Appetite	Simplified Nutrition Appetite Questionnaire (SNAQ)[54]	□	□	□

For peer review only - http://bmjopen.bmj.com/site/about/guidelines.xhtml

For peer review only

Body composition	Bioelectric Impedance Analysis of body fat and fat free mass (Body Stat 1500 MDD)	☐	☐	☐
Anthropometry	Height (stadiometer) and Weight (digital scales)	☐	☐	☐
Physical activity				
Physical activity	Recent Physical Activity Questionnaire (RPAQ)[55]	☐	☐	☐
Cognitive function				
Cognitive function	Catch-Cog,[56] Trail Making Test A and B[57] and Delayed Recall Test[58]	☐	☐	☐
Physical strength and function				
Functional capacity	Short Physical Performance Battery (SPPB) [59]	☐	☐	☐
Muscle strength	Hand Grip Strength (Dynamometer)	☐	☐	☐
Psychosocial				
Health-Related Quality of Life	SF-36 Health Survey[60] questionnaire	☐	☐	☐
Depressive symptoms	Geriatric Depression Scale[61] questionnaire	☐	☐	☐
Blood Pressure				
Blood Pressure	Clinic measured blood pressure (Omron Healthcare)	☐	☐	☐
Biochemical Measures				
Blood biomarkers	Nutritional: vitamins A,E,C, carotenoids and fatty acids. cardiometabolic: lipid profile, HbA1c ,glucose and insulin. Inflammatory: hs-CRP, IL-6.	☐	☐	☐
**Evaluation**				
Study evaluation	Questionnaire			☐

Primary outcome

The primary study outcome is between-group difference in change in nutritional status at 6-months using the validated MNA score (MNA® 0-30).[53] The MNA is the most widely validated tool for detecting undernutrition risk in older adults[62] and is shown to respond to dietary intervention.[63]

Secondary outcomes

Nutrition and body composition

Dietary behaviour will be reported using the PROMED diet score (range 0-14) adapted from a validated Spanish Mediterranean diet score[64] to incorporate the higher protein food targets and tailored for a Northern European population. Higher scores indicate increased concordance with the PROMED diet. Overall dietary intake will be assessed using a 4-day prospective food diary. Participants will be instructed to record all items of food and beverages consumed over four consecutive days (two weekend days) at home and provide

For peer review only

For peer review only - http://bmjopen.bmj.com/site/about/guidelines.xhtml

details of the portion size consumed, cooking methods and named brands. Data on self-reported appetite will also be collected using the 4-item Simplified Nutrition Appetite Questionnaire (SNAQ) validated for older community dwelling adults.[54] Lower SNAQ scores indicate poorer appetite. Body composition will be assessed using non-invasive bioelectrical impedance analysis (BodyStat 1500 MDD) to estimate fat and fat free mass.[65] Anthropometric measures of weight and height will also be taken using digital scales and a stadiometer, respectively.

Physical activity

Time spent on different intensity levels of physical activity will be self-reported using a validated Recent Physical Activity Questionnaire (RPAQ) across four domains: home, commuting, occupational activity (if appropriate), and leisure activity over the previous 4 weeks.[55]

Cognitive function

A brief neuropsychological test battery (NTB) will be administered by a trained researcher to capture cognitive domains that are shown to be sensitive to progression in early stages of memory decline: global cognition, executive functioning, episodic and working memory, information processing speed and sustained attention.[56] The NTB will include the Catch-Cog assessment[56] comprising (i) three Alzheimer's Disease Assessment Scale–Cognitive subscales (Word Recognition (score range 0-12), Word Recall (score range 0-10), and Orientation (score range 0-8)) to assess episodic memory (ii) the Controlled Oral Word Association Test (number of acceptable words in one minute); Category Fluency Test (number of animals named in one minute); (iii) Digit Symbol Substitution Test (total number of correct symbols in 90 seconds) and (iv) Digit Span Backward (score range 0-14) to assess to assess executive function and working memory. The Trail Making Test (A and B) will be used to assess information processing speed and attention.[57] A delayed recall test of recalled learned words after a 5-minute delay (score range 0–10) will be administered, with higher scores indicating better memory performance.

Physical strength and function

Lower extremity functional status will be measured using the Short Physical Performance Battery (SPPB).[59] The SPPB includes three timed tasks to assess walking speed, chair stand and standing balance, with scores ranging from 0 (worst performance) to 12 (best performance). Muscle (handgrip) strength will be measured with a calibrated dynamometer (Type) (kg) using a standard procedure.[66]

Psychosocial

Health-related Quality of Life (HR QoL) will be determined by using the Short Form-36 (SF-36) 36-item questionnaire[60] which measures eight domains of health status: physical functioning, role limitations due to physical issues, role limitation due to emotional problems, energy/fatigue, emotional well-being, social functioning, pain, and general health perception. SF-36 scores range from 0-100 with higher scores indicating better HR QoL.[60] Depressive symptoms will be determined using the 15-item Geriatric Depression Scale (GDS) – Short Form). GDS scores range from 0 to 15, with a score ≥5 suggesting depression.[61]

Blood pressure

Systolic and diastolic blood pressure (mmHg) will be measured three times in the dominant arm after a 5-minute rest in the seated position using a calibrated automated device approved

For peer review only

by the British Hypertension Society (Omron Healthcare). The first measure will be discarded and the average of the final two measurements will be recorded.

Biochemical measures

A 50 ml fasting peripheral venous blood sample will be taken at the start of each study assessment for metabolomic profiling and biomarker measurement. Anonymised samples will be processed in the Nutrition laboratory at QUB according to standardised protocols and stored in aliquots at -80°C until analysis. Biochemical markers relating to nutrition (vitamins A, C, E, carotenoids, and fatty acids), metabolic (lipid profile, HbA1c, glucose and insulin), and inflammation (hs-CRP, IL-6) will be measured. Plasma samples will be transferred to University College Dublin for metabolomics analysis.

Evaluation

A questionnaire will be administered at the end of the study to determine acceptability and tolerance of the intervention and the overall experience of taking part in the study. Participants will be asked to return diet and exercise logs to evaluate engagement with the intervention.

Sample Size

As no prior studies have evaluated the effects of whole diet and exercise on nutritional status and cognition, the sample size calculation is based on a standard deviation of the change in MNA score of 4 points over a similar period from the control group of a previous nutrition intervention.[63] A total of 28 completing in each intervention and control group would provide over 80% power to detect a difference in the three means, based upon a statistically significant ANOVA result at the 5% level, hypothesising a true increase in MNA of 3 or more in both intervention groups, but not the control group. Loss to follow-up is estimated from our prior experience of conducting lifestyle trials in older adults[66-68] where attrition ranges from 6%[68] to 30%.[67] Considering, FINGER[42] evaluated an intensive multidomain lifestyle intervention among older adults at increased dementia risk and reported 12% withdrawal over 2 years. We anticipate dropout at 20% and therefore have added 20% to the sample size to account for non-compliance, dropout, or discontinuation.

Data management and statistical analysis plan

Data will be de-identified and stored securely according to General Data Protection Regulation (GDPR) and UK Data Protection Act 2018 in QUB. There are no plans for interim study analysis. Participant characteristics by allocated group will be compared using analysis of variance for continuous variables and Chi square test for dichotomous variables. Skewed variables will be log transformed prior to analysis. An intention-to-treat analysis will be conducted using multiple linear regression model to compare MNA score at 6 months between the three groups adjusting for baseline. Dunnett's procedure will then be used to compare each intervention group to the controls, accounting for multiple testing. Analysis of secondary outcomes will utilise a similar method and these exploratory analyses will be interpreted cautiously. Sensitivity analyses will be conducted to evaluate the diet and exercise behavioural changes maintained over 6-months and to determine the effect of intervention compliance on study-end-points. Multiple imputations for missing data will be used if required. Analyses will be performed using STATA Statistical Software: StataCorp LLC.

For peer review only - http://bmjopen.bmj.com/site/about/guidelines.xhtml

For peer review only

Ethics and dissemination

Ethical approval has been received from Office for Research Ethics Committee (OREC) (Ref 21/NW/0215) and the trial has been registered on ClinicalTrials.gov (NCT 05166564). The study will be reported according to CONSORT guidelines for parallel group randomised trials.[69] Written informed consent will be obtained from all study participants prior to study enrolment and any adverse events will be reported to the study sponsor (QUB) and OREC.

Study findings will be of interest to a range of stakeholders in the areas of healthy ageing, preventable malnutrition, cognitive decline, and dementia risk reduction, including academics, the public, healthcare professionals and clinicians, charitable organisations, and industry. Findings will be published in academic journals and published in open access repositories, for example the JPI-HDHL Meta Database (http://www.jpihdhl.eu) knowledge transfer platform.

COVID-19

The research team will comply with public health and QUB guidance regarding COVID-19. Several strategies will be implemented to mitigate COVID-19 risk: (i) COVID-19 risk screening for participants prior to study visits (ii) optional home study visits, (iii) optional remote delivery of intervention support, (iv) personal protective equipment for researchers conducting study assessments, (v) participant face coverings for study visits where possible, (vi) social distancing measures followed where possible, and (vii) researcher lateral flow testing pre-study visit.

DISCUSSION

To our knowledge, PROMED-EX is the first study aimed to evaluate the combined effects of a protein-enriched MedDiet and exercise on nutritional status and cognition in older adults at risk of undernutrition with SCD. Lifestyle interventions to address modifiable risk factors for dementia are likely to have a more significant public health impact when implemented at an early stage of disease and in high-risk populations. PROMED-EX will generate new knowledge about how a targeted lifestyle intervention delivered in a 'real-world' setting can impact nutritional and cognitive status among older individuals at risk of undernutrition and cognitive decline. A further novel aspect of the study is the planned evaluation that will provide insight into lifestyle behaviour changes that are feasible and acceptable for the intended population.

A key strength is the inclusion of a validated neurocognitive test battery in the current study allowing a comprehensive assessment of performance in both memory and non-memory domains of cognition. Prior studies have relied on the limited MMSE global cognition to measure cognitive performance. While MMSE is a validated screener for cognitive impairment, it provides limited examination of visuospatial cognitive ability, long-term memory, and language functions. It also has a "ceiling" effect whereby individuals can still score quite highly even though they may have significant cognitive impairment.[70]

A further strength is the integration of state-of-the-art metabolomics, metabolic, inflammatory, and nutritional biomarkers in the study will help to elucidate biological

For peer review only - http://bmjopen.bmj.com/site/about/guidelines.xhtml

mechanisms underlying how diet can influence nutrition status and cognition, and how exercise may act to modify these processes. Furthermore, the interventions will be delivered using a personalised approach with incorporation of evidence-based techniques to support and encourage behaviour change. Study participants will also benefit from written educational resources that have been co-designed with PPI members to support the desired diet and exercise behaviour changes. The ongoing collaboration with PPI members in the trial will undoubtedly optimise the dissemination of study findings.

There are some limitations to the study design that should be considered. The optimum period for lifestyle-induced change in nutritional status is not clear, and the duration of the current study intervention is shorter than in other multidomain trials; [42] however, improvement in MNA score in response to nutrition intervention has been observed in 12 weeks. [63] Furthermore, 6 months should be sufficient duration to detect increases in nutrient biomarkers in response to the interventions, especially in a high-risk population. The pragmatic study design also means that participants will not be blinded to treatment allocation, consistent with other lifestyle interventions. However, data will be analysed by a blinded member of the study team to minimise detection bias.

Conclusion

PROMED-EX focuses on undernutrition and cognitive impairment which are major contributors to mortality and disability in older age. The findings will provide new and critical insight into how modifications of diet and exercise behaviour can act to prevent undernutrition in older citizens at risk of undernutrition and cognitive decline. The study will advance understanding of how the protein enriched MedDiet influences nutritional status and cognitive performance, and whether biological pathways are altered by the addition of exercise. We expect the study findings to inform future dietary guidelines for prevention of undernutrition to promote healthy neurocognitive ageing, and guide the design of future larger trials to test the efficacy of diet and exercise on cognition.

Trial status:

Ongoing: Recruitment began in January 2022 and will complete in July 2023. Data collection is scheduled to complete in December 2023.

Contributions:

CME, LB, CC, FP, LdG, BMG, MMK, JVW, DV contributed to the conception and design of the trial. CME and NW drafted the manuscript. All authors contributed to the editing of manuscript and revising it critically for intellectual content. The Trial Steering Committee approved this manuscript submission. CME gave final manuscript approval and is the corresponding and senior author.

Sponsor: The study sponsor is Queen’s University Belfast (Ref: B21/16) in accordance with UK Policy Framework for Health and Social Care.

Funding

This work is supported by funding from national funding organisations (listed below) under the umbrella of the European Joint Programming Initiative "A Healthy Diet for a Healthy

For peer review only - http://bmjopen.bmj.com/site/about/guidelines.xhtml

Life" (JPI HDHL) and of the ERA NET Cofund ERA HDHL (GA N 696295 of the EU Horizon 2020 Research and Innovation Programme).

UK. Research & Innovation (UKRI): Biotechnology and Biological Sciences Research Council (BBSRC) and Medical Research Council (MRC) - Queen's University Belfast

Ministry of Universities and Research (MUR) Italy - National Research Council (CNR), Institute of Biomedical Technologies, Epidemiology Unit and Neuroscience Institute, Aging Branch; and University of Padova Geriatric Unit, Department of Medicine.

Health Research Board (HRB) Ireland - University College Dublin (UCD), Institute of Food and Health.

Funders and study sponsors had no input in any aspect of study design; writing of the protocol; and the decision to submit the protocol for publication.

Competing interests none declared

Ethics approval

Office for Research Ethics Committee (OREC).

Protocol Version 3: Any amendments will be reviewed and approved by Trial Steering Committee and OREC prior to implementation.

For peer review only

REFERENCES

1. Cederholm T, Bosaeus I, Barazzoni R, et al. Diagnostic criteria for malnutrition – An ESPEN Consensus Statement. Clinical Nutrition 2015;34(3):335-40. doi: 10.1016/j.clnu.2015.03.001
2. Stratton RJ Smith T, Gabe S. Managing malnutrition to improve lives and save money. BAPEN, 2018.
3. Sanders CL, Wengreen HJ, Schwartz S, et al. Nutritional Status is Associated With Severe Dementia and Mortality: The Cache County Dementia Progression Study. Alzheimer Dis Assoc Disord 2018;32(4):298-304. doi: 10.1097/wad.0000000000000274
4. Guerin O, Soto ME, Brocker P, et al. Nutritional status assessment during Alzheimer's disease: results after one year (the REAL French Study Group). J Nutr Health Aging 2005;9(2):81-4.

5. Qizilbash N, Gregson J, Johnson ME, et al. BMI and risk of dementia in two million people over two decades: a retrospective cohort study. Lancet Diabetes Endocrinol 2015;3(6):431-36. doi: 10.1016/s2213-8587(15)00033-9 [published Online First: 20150409]
6. Lee CM, Woodward M, Batty GD, et al. Association of anthropometry and weight change with risk of dementia and its major subtypes: A meta-analysis consisting 2.8 million adults with 57 294 cases of dementia. Obes Rev 2020;21(4):e12989. doi: 10.1111/obr.12989 [published Online First: 20200103]
7. Knopman DS, Edland SD, Cha RH, et al. Incident dementia in women is preceded by weight loss by at least a decade. Neurology 2007;69(8):739-46. doi: 10.1212/01.wnl.0000267661.65586.33
8. LeBlanc ES, Rizzo JH, Pedula KL, et al. Weight Trajectory over 20 Years and Likelihood of Mild Cognitive Impairment or Dementia Among Older Women. J Am Geriatr Soc 2017;65(3):511-19. doi: 10.1111/jgs.14552 [published Online First: 20161219]
9. Volkert D, Kiesswetter E, Cederholm T, et al. Development of a Model on Determinants of Malnutrition in Aged Persons: A MaNuEL Project. Gerontol Geriatr Med 2019;5:2333721419858438-38. doi: 10.1177/2333721419858438
10. Burns JM, Johnson DK, Watts A, et al. Reduced lean mass in early Alzheimer disease and its association with brain atrophy. Arch Neurol 2010;67(4):428-33. doi: 10.1001/archneurol.2010.38
11. Yu JH, Kim REY, Jung JM, et al. Sarcopenia is associated with decreased gray matter volume in the parietal lobe: a longitudinal cohort study. BMC Geriatr 2021;21(1):622. doi: 10.1186/s12877-021-02581-4 [published Online First: 20211102]
12. Jimenez A, Pegueroles J, Carmona-Iragui M, et al. Weight loss in the healthy elderly might be a non-cognitive sign of preclinical Alzheimer's disease. Oncotarget 2017;8(62):104706-16. doi: 10.18632/oncotarget.22218 [published Online First: 20171031]
13. McGrattan AM, McGuinness B, McKinley MC, et al. Diet and Inflammation in Cognitive Ageing and Alzheimer's Disease. Curr Nutr Rep 2019;8(2):53-65. doi: 10.1007/s13668-019-0271-4
14. Ahima RS, Antwi DA. Brain regulation of appetite and satiety. Endocrinol Metab Clin North Am 2008;37(4):811-23. doi: 10.1016/j.ecl.2008.08.005
15. Kimura A, Sugimoto T, Niida S, et al. Association Between Appetite and Sarcopenia in Patients With Mild Cognitive Impairment and Early-Stage Alzheimer's Disease: A Case-Control Study. Front Nutr 2018;5:128. doi: 10.3389/fnut.2018.00128 [published Online First: 20181218]
16. Attems J, Walker L, Jellinger KA. Olfaction and Aging: A Mini-Review. Gerontology 2015;61(6):485-90. doi: 10.1159/000381619 [published Online First: 20150509]
17. Doorduijn AS, van de Rest O, van der Flier WM, et al. Energy and Protein Intake of Alzheimer's Disease Patients Compared to Cognitively Normal Controls: Systematic Review. J Am Med Dir Assoc 2019;20(1):14-21. doi: 10.1016/j.jamda.2018.06.019 [published Online First: 20180809]
18. Alagiakrishnan K, Bhanji RA, Kurian M. Evaluation and management of oropharyngeal dysphagia in different types of dementia: A systematic review. Archives of Gerontology and Geriatrics 2013;56(1):1-9. doi: https://doi.org/10.1016/j.archger.2012.04.011
19. Hiller AJ, Ishii M. Disorders of Body Weight, Sleep and Circadian Rhythm as Manifestations of Hypothalamic Dysfunction in Alzheimer's Disease. Front Cell Neurosci 2018;12:471. doi: 10.3389/fncel.2018.00471 [published Online First: 20181205]
20. Honda Y, Meguro K, Meguro M, et al. Social withdrawal of persons with vascular dementia associated with disturbance of basic daily activities, apathy, and impaired social judgment. Care Manag J 2013;14(2):108-13. doi: 10.1891/1521-0987.14.2.108

For peer review only - http://bmjopen.bmj.com/site/about/guidelines.xhtml

21. Volkert D, Chourdakis M, Faxen-Irving G, et al. ESPEN guidelines on nutrition in dementia. Clin Nutr 2015;34(6):1052-73. doi: 10.1016/j.clnu.2015.09.004 [published Online First: 20150925]
22. Reinders I, Wijnhoven HAH, Jyväkorpi SK, et al. Effectiveness and cost-effectiveness of personalised dietary advice aiming at increasing protein intake on physical functioning in community-dwelling older adults with lower habitual protein intake: rationale and design of the PROMISS randomised controlled trial. BMJ Open 2020;10(11):e040637. doi: 10.1136/bmjopen-2020-040637 [published Online First: 20201120]
23. Endevelt R, Lemberger J, Bregman J, et al. Intensive dietary intervention by a dietitian as a case manager among community dwelling older adults: the EDIT study. J Nutr Health Aging 2011;15(8):624-30. doi: 10.1007/s12603-011-0074-9
24. Wouters-Wesseling W, Vos AP, Van Hal M, et al. The effect of supplementation with an enriched drink on indices of immune function in frail elderly. J Nutr Health Aging 2005;9(4):281-6.
25. Blondal BS, Geirsdottir OG, Halldorsson TI, et al. HOMEFOOD randomised trial – Six-month nutrition therapy improves quality of life, self-rated health, cognitive function, and depression in older adults after hospital discharge. Clinical Nutrition ESPEN 2022;48:74-81. doi: https://doi.org/10.1016/j.clnesp.2022.01.010
26. Casas R, Sacanella E, Urpí-Sardà M, et al. Long-Term Immunomodulatory Effects of a Mediterranean Diet in Adults at High Risk of Cardiovascular Disease in the PREvención con DIeta MEDiterránea (PREDIMED) Randomized Controlled Trial. J Nutr 2016;146(9):1684-93. doi: 10.3945/jn.115.229476 [published Online First: 20160720]
27. Berti V, Walters M, Sterling J, et al. Mediterranean diet and 3-year Alzheimer brain biomarker changes in middle-aged adults. Neurology 2018;90(20):e1789-e98. doi: 10.1212/wnl.0000000000005527 [published Online First: 20180413]
28. McEvoy CT, Hoang T, Sidney S, et al. Dietary patterns during adulthood and cognitive performance in midlife: The CARDIA study. Neurology 2019;92(14):e1589-e99. doi: 10.1212/wnl.0000000000007243 [published Online First: 20190306]
29. van de Rest O, Berendsen AA, Haveman-Nies A, et al. Dietary patterns, cognitive decline, and dementia: a systematic review. Adv Nutr 2015;6(2):154-68. doi: 10.3945/an.114.007617
30. Limongi F, Siviero P, Bozanic A, et al. The Effect of Adherence to the Mediterranean Diet on Late-Life Cognitive Disorders: A Systematic Review. Journal of the American Medical Directors Association 2020;21(10):1402-09. doi: https://doi.org/10.1016/j.jamda.2020.08.020
31. Singh B, Parsaik AK, Mielke MM, et al. Association of mediterranean diet with mild cognitive impairment and Alzheimer's disease: a systematic review and meta-analysis. J Alzheimers Dis 2014;39(2):271-82. doi: 10.3233/jad-130830
32. Feart C, Samieri C, Barberger-Gateau P. Mediterranean diet and cognitive health: an update of available knowledge. Curr Opin Clin Nutr Metab Care 2015;18(1):51-62. doi: 10.1097/mco.0000000000000131
33. Castro-Quezada I, Román-Viñas B, Serra-Majem L. The Mediterranean diet and nutritional adequacy: a review. Nutrients 2014;6(1):231-48. doi: 10.3390/nu6010231
34. Kiesswetter E, Sieber CC, Volkert D. Protein intake in older people : Why, how much and how? Z Gerontol Geriatr 2020;53(4):285-89. doi: 10.1007/s00391-020-01723-4 [published Online First: 20200414]
35. Volkert D, Beck AM, Cederholm T, et al. ESPEN guideline on clinical nutrition and hydration in geriatrics. Clinical Nutrition 2019;38(1):10-47. doi: https://doi.org/10.1016/j.clnu.2018.05.024

For peer review only - http://bmjopen.bmj.com/site/about/guidelines.xhtml

36. Okamura T, Miki A, Hashimoto Y, et al. Shortage of energy intake rather than protein intake is associated with sarcopenia in elderly patients with type 2 diabetes: A cross-sectional study of the KAMOGAWA-DM cohort. J Diabetes 2019;11(6):477-83. doi: 10.1111/1753-0407.12874 [published Online First: 20181204]
37. Bauer J, Biolo G, Cederholm T, et al. Evidence-based recommendations for optimal dietary protein intake in older people: a position paper from the PROT-AGE Study Group. J Am Med Dir Assoc 2013;14(8):542-59. doi: 10.1016/j.jamda.2013.05.021 [published Online First: 20130716]
38. Granic A, Mendonça N, Sayer AA, et al. Effects of dietary patterns and low protein intake on sarcopenia risk in the very old: The Newcastle 85+ study. Clin Nutr 2020;39(1):166-73. doi: 10.1016/j.clnu.2019.01.009 [published Online First: 20190121]
39. Livingston G, Sommerlad A, Orgeta V, et al. Dementia prevention, intervention, and care. Lancet 2017;390(10113):2673-734. doi: 10.1016/s0140-6736(17)31363-6 [published Online First: 20170720]
40. Kirk-Sanchez NJ, McGough EL. Physical exercise and cognitive performance in the elderly: current perspectives. Clin Interv Aging 2014;9:51-62. doi: 10.2147/cia.S39506 [published Online First: 20131218]
41. Lista I, Sorrentino G. Biological Mechanisms of Physical Activity in Preventing Cognitive Decline. Cellular and Molecular Neurobiology 2010;30(4):493-503. doi: 10.1007/s10571-009-9488-x
42. Ngandu T, Lehtisalo J, Solomon A, et al. A 2 year multidomain intervention of diet, exercise, cognitive training, and vascular risk monitoring versus control to prevent cognitive decline in at-risk elderly people (FINGER): a randomised controlled trial. Lancet 2015;385(9984):2255-63. doi: 10.1016/s0140-6736(15)60461-5 [published Online First: 20150312]
43. Trommelen J, Betz MW, van Loon LJC. The Muscle Protein Synthetic Response to Meal Ingestion Following Resistance-Type Exercise. Sports Med 2019;49(2):185-97. doi: 10.1007/s40279-019-01053-5
44. McGlory C, van Vliet S, Stokes T, et al. The impact of exercise and nutrition on the regulation of skeletal muscle mass. J Physiol 2019;597(5):1251-58. doi: 10.1113/jp275443 [published Online First: 20180801]
45. O'Neill R, Brennan L, Prinelli F, et al. PROtein enriched MEDiterranean diet to combat undernutrition and promote healthy neuroCOGnitive ageing in older adults: The PROMED-COG consortium project. Nutrition Bulletin 2022 doi: 10.1111/nbu.12571
46. Chan A-W, Tetzlaff JM, Gøtzsche PC, et al. SPIRIT 2013 explanation and elaboration: guidance for protocols of clinical trials. BMJ : British Medical Journal 2013;346:e7586. doi: 10.1136/bmj.e7586
47. Kaiser MJ, Bauer JM, Ramsch C, et al. Validation of the Mini Nutritional Assessment short-form (MNA®-SF): A practical tool for identification of nutritional status. JNHA - The Journal of Nutrition, Health and Aging 2009;13(9):782. doi: 10.1007/s12603-009-0214-7
48. CDC. BRFSS Questionnaires. July ed: Centres for Disease Control and Prevention, 2020.
49. Excellence NNIfHaC. Nutrition support for adults: oral nutrition support, enteral tube feeding and parenteral nutrition. Clinical guideline CG32. ONLINE, 2017.
50. Skelly F. Community-based exercise rehabilitation in a diverse chronic disease population. Dublin City University, 2021.
51. Lea JWD, O'Driscoll JM, Hulbert S, et al. Convergent Validity of Ratings of Perceived Exertion During Resistance Exercise in Healthy Participants: A Systematic Review and Meta-Analysis. Sports Medicine - Open 2022;8(1):2. doi: 10.1186/s40798-021-00386-8
52. Lin H, Xu D, Yang M, et al. Behaviour change techniques that constitute effective planning interventions to improve physical activity and diet behaviour for people with

For peer review only

chronic conditions: a systematic review. BMJ Open 2022;12(8):e058229. doi: 10.1136/bmjopen-2021-058229 [published Online First: 20220822]
53. Guigoz Y, Vellas B. Nutritional Assessment in Older Adults : MNA® 25 years of a Screening Tool and a Reference Standard for Care and Research; What Next? J Nutr Health Aging 2021;25(4):528-83. doi: 10.1007/s12603-021-1601-y
54. Wilson MM, Thomas DR, Rubenstein LZ, et al. Appetite assessment: simple appetite questionnaire predicts weight loss in community-dwelling adults and nursing home residents. Am J Clin Nutr 2005;82(5):1074-81. doi: 10.1093/ajcn/82.5.1074
55. Besson H, Brage S, Jakes RW, et al. Estimating physical activity energy expenditure, sedentary time, and physical activity intensity by self-report in adults. Am J Clin Nutr 2010;91(1):106-14. doi: 10.3945/ajcn.2009.28432 [published Online First: 20091104]
56. Jutten RJ, Harrison JE, Brunner AJ, et al. The Cognitive-Functional Composite is sensitive to clinical progression in early dementia: Longitudinal findings from the Catch-Cog study cohort. Alzheimers Dement (N Y) 2020;6(1):e12020. doi: 10.1002/trc2.12020 [published Online First: 20200417]
57. Lezak MD, Howieson DB, Loring DW. Neuropsychological Assessment. 4th ed: New York: Oxford University Press 2004.
58. Zhao Q, Lv Y, Zhou Y, et al. Short-term delayed recall of auditory verbal learning test is equivalent to long-term delayed recall for identifying amnestic mild cognitive impairment. PLoS One 2012;7(12):e51157. doi: 10.1371/journal.pone.0051157 [published Online First: 20121207]
59. Guralnik JM, Simonsick EM, Ferrucci L, et al. A short physical performance battery assessing lower extremity function: association with self-reported disability and prediction of mortality and nursing home admission. J Gerontol 1994;49(2):M85-94. doi: 10.1093/geronj/49.2.m85
60. Ware JE, Jr., Sherbourne CD. The MOS 36-item short-form health survey (SF-36). I. Conceptual framework and item selection. Med Care 1992;30(6):473-83.
61. Yesavage JA, Brink TL, Rose TL, et al. Development and validation of a geriatric depression screening scale: a preliminary report. J Psychiatr Res 1982;17(1):37-49. doi: 10.1016/0022-3956(82)90033-4
62. Vellas B, Villars H, Abellan G, et al. Overview of the MNA--Its history and challenges. J Nutr Health Aging 2006;10(6):456-63; discussion 63-5.
63. Luger E, Dorner TE, Haider S, et al. Effects of a Home-Based and Volunteer-Administered Physical Training, Nutritional, and Social Support Program on Malnutrition and Frailty in Older Persons: A Randomized Controlled Trial. J Am Med Dir Assoc 2016;17(7):671.e9-71.e16. doi: 10.1016/j.jamda.2016.04.018
64. Schröder H, Fitó M, Estruch R, et al. A short screener is valid for assessing Mediterranean diet adherence among older Spanish men and women. J Nutr 2011;141(6):1140-5. doi: 10.3945/jn.110.135566 [published Online First: 20110420]
65. Genton L, Karsegard VL, Kyle UG, et al. Comparison of Four Bioelectrical Impedance Analysis Formulas in Healthy Elderly Subjects. Gerontology 2001;47:315 - 23.
66. Marseglia A, Xu W, Fratiglioni L, et al. Effect of the NU-AGE Diet on Cognitive Functioning in Older Adults: A Randomized Controlled Trial. Front Physiol 2018;9:349-49. doi: 10.3389/fphys.2018.00349
67. McEvoy CT, Moore SE, Appleton KM, et al. Trial to Encourage Adoption and Maintenance of a Mediterranean Diet (TEAM-MED): Protocol for a Randomised Feasibility Trial of a Peer Support Intervention for Dietary Behaviour Change in Adults at High Cardiovascular Disease Risk. Int J Environ Res Public Health 2018;15(6) doi: 10.3390/ijerph15061130 [published Online First: 20180531]

For peer review only

For peer review only - http://bmjopen.bmj.com/site/about/guidelines.xhtml

68. van de Rest O, van der Zwaluw NL, Tieland M, et al. Effect of resistance-type exercise training with or without protein supplementation on cognitive functioning in frail and pre-frail elderly: secondary analysis of a randomized, double-blind, placebo-controlled trial. Mech Ageing Dev 2014;136-137:85-93. doi: 10.1016/j.mad.2013.12.005 [published Online First: 20131227]
69. Moher D, Hopewell S, Schulz KF, et al. CONSORT 2010 Explanation and Elaboration: Updated guidelines for reporting parallel group randomised trials. J Clin Epidemiol 2010;63(8):e1-37. doi: 10.1016/j.jclinepi.2010.03.004 [published Online First: 20100325]
70. Franco-Marina F, García-González JJ, Wagner-Echeagaray F, et al. The Mini-mental State Examination revisited: ceiling and floor effects after score adjustment for educational level in an aging Mexican population. Int Psychogeriatr 2010;22(1):72-81. doi: 10.1017/s1041610209990822 [published Online First: 20090907]

Figure 1: Overview of PROMED-EX Study Design

For peer review only

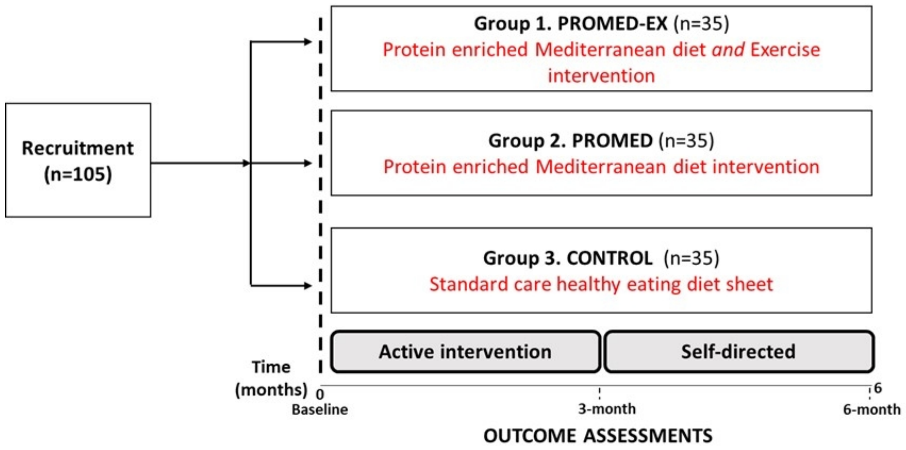

Overview of PROMED-EX Study Design

159x72mm (150 x 150 DPI)

STANDARD PROTOCOL ITEMS: RECOMMENDATIONS FOR INTERVENTIONAL TRIALS

SPIRIT 2013 Checklist: Recommended items to address in a clinical trial protocol and related documents*

Section/item	Item No	Description	Addressed on page number
**Administrative information**			
Title	1	Descriptive title identifying the study design, population, interventions, and, if applicable, trial acronym	**1**
Trial registration	2a	Trial identifier and registry name. If not yet registered, name of intended registry	**1**
	2b	All items from the World Health Organization Trial Registration Data Set	**1**
Protocol version	3	Date and version identifier	**14**
Funding	4	Sources and types of financial, material, and other support	**14**
Roles and responsibilities	5a	Names, affiliations, and roles of protocol contributors	**1,14**
	5b	Name and contact information for the trial sponsor	**1**
	5c	Role of study sponsor and funders, if any, in study design; collection, management, analysis, and interpretation of data; writing of the report; and the decision to submit the report for publication, including whether they will have ultimate authority over any of these activities	**14**
	5d	Composition, roles, and responsibilities of the coordinating centre, steering committee, endpoint adjudication committee, data management team, and other individuals or groups overseeing the trial, if applicable (see Item 21a for data monitoring committee)	**Appendix 2**

For peer review only

**Introduction**			
Background and rationale	6a	Description of research question and justification for undertaking the trial, including summary of relevant studies (published and unpublished) examining benefits and harms for each intervention	**3,4**
	6b	Explanation for choice of comparators	**4**
Objectives	7	Specific objectives or hypotheses	**4**
Trial design	8	Description of trial design including type of trial (eg, parallel group, crossover, factorial, single group), allocation ratio, and framework (eg, superiority, equivalence, noninferiority, exploratory)	**4**
**Methods: Participants, interventions, and outcomes**			
Study setting	9	Description of study settings (eg, community clinic, academic hospital) and list of countries where data will be collected. Reference to where list of study sites can be obtained	**5**
Eligibility criteria	10	Inclusion and exclusion criteria for participants. If applicable, eligibility criteria for study centres and individuals who will perform the interventions (eg, surgeons, psychotherapists)	**5,6**
Interventions	11a	Interventions for each group with sufficient detail to allow replication, including how and when they will be administered	**6-8**
	11b	Criteria for discontinuing or modifying allocated interventions for a given trial participant (eg, drug dose change in response to harms, participant request, or improving/worsening disease)	**N/A**
	11c	Strategies to improve adherence to intervention protocols, and any procedures for monitoring adherence (eg, drug tablet return, laboratory tests)	**8**
	11d	Relevant concomitant care and interventions that are permitted or prohibited during the trial	**6-8**
Outcomes	12	Primary, secondary, and other outcomes, including the specific measurement variable (eg, systolic blood pressure), analysis metric (eg, change from baseline, final value, time to event), method of aggregation (eg, median, proportion), and time point for each outcome. Explanation of the clinical relevance of chosen efficacy and harm outcomes is strongly recommended	**10,11**
Participant timeline	13	Time schedule of enrolment, interventions (including any run-ins and washouts), assessments, and visits for participants. A schematic diagram is highly recommended (see Figure)	**5 (Figure 1)**

For peer review only - http://bmjopen.bmj.com/site/about/guidelines.xhtml

Sample size	14	Estimated number of participants needed to achieve study objectives and how it was determined, including clinical and statistical assumptions supporting any sample size calculations	**11**
Recruitment	15	Strategies for achieving adequate participant enrolment to reach target sample size	**5**
**Methods: Assignment of interventions (for controlled trials)**			
Allocation:			
Sequence generation	16a	Method of generating the allocation sequence (eg, computer-generated random numbers), and list of any factors for stratification. To reduce predictability of a random sequence, details of any planned restriction (eg, blocking) should be provided in a separate document that is unavailable to those who enrol participants or assign interventions	**6**
Allocation concealment mechanism	16b	Mechanism of implementing the allocation sequence (eg, central telephone; sequentially numbered, opaque, sealed envelopes), describing any steps to conceal the sequence until interventions are assigned	**6**
Implementation	16c	Who will generate the allocation sequence, who will enrol participants, and who will assign participants to interventions	**6**
Blinding (masking)	17a	Who will be blinded after assignment to interventions (eg, trial participants, care providers, outcome assessors, data analysts), and how	**6**
	17b	If blinded, circumstances under which unblinding is permissible, and procedure for revealing a participant's allocated intervention during the trial	**N/A**
**Methods: Data collection, management, and analysis**			
Data collection methods	18a	Plans for assessment and collection of outcome, baseline, and other trial data, including any related processes to promote data quality (eg, duplicate measurements, training of assessors) and a description of study instruments (eg, questionnaires, laboratory tests) along with their reliability and validity, if known. Reference to where data collection forms can be found, if not in the protocol	**8-11**
	18b	Plans to promote participant retention and complete follow-up, including list of any outcome data to be collected for participants who discontinue or deviate from intervention protocols	**11**

Data management	19	Plans for data entry, coding, security, and storage, including any related processes to promote data quality (eg, double data entry; range checks for data values). Reference to where details of data management procedures can be found, if not in the protocol	**12**
Statistical methods	20a	Statistical methods for analysing primary and secondary outcomes. Reference to where other details of the statistical analysis plan can be found, if not in the protocol	**11**
	20b	Methods for any additional analyses (eg, subgroup and adjusted analyses)	**N/A**
	20c	Definition of analysis population relating to protocol non-adherence (eg, as randomised analysis), and any statistical methods to handle missing data (eg, multiple imputation)	**12**
**Methods: Monitoring**			
Data monitoring	21a	Composition of data monitoring committee (DMC); summary of its role and reporting structure; statement of whether it is independent from the sponsor and competing interests; and reference to where further details about its charter can be found, if not in the protocol. Alternatively, an explanation of why a DMC is not needed	**N/A -Appendix 1**
	21b	Description of any interim analyses and stopping guidelines, including who will have access to these interim results and make the final decision to terminate the trial	**N/A (no interim analysis)**
Harms	22	Plans for collecting, assessing, reporting, and managing solicited and spontaneously reported adverse events and other unintended effects of trial interventions or trial conduct	**11**
Auditing	23	Frequency and procedures for auditing trial conduct, if any, and whether the process will be independent from investigators and the sponsor	**None**
**Ethics and dissemination**			
Research ethics approval	24	Plans for seeking research ethics committee/institutional review board (REC/IRB) approval	**12**
Protocol amendments	25	Plans for communicating important protocol modifications (eg, changes to eligibility criteria, outcomes, analyses) to relevant parties (eg, investigators, REC/IRBs, trial participants, trial registries, journals, regulators)	**14**

For peer review only - http://bmjopen.bmj.com/site/about/guidelines.xhtml

Consent or assent	26a	Who will obtain informed consent or assent from potential trial participants or authorised surrogates, and how (see Item 32)	**6, Appendix 4**
	26b	Additional consent provisions for collection and use of participant data and biological specimens in ancillary studies, if applicable	**Appendix 4**
Confidentiality	27	How personal information about potential and enrolled participants will be collected, shared, and maintained in order to protect confidentiality before, during, and after the trial	**12**
Declaration of interests	28	Financial and other competing interests for principal investigators for the overall trial and each study site	**14**
Access to data	29	Statement of who will have access to the final trial dataset, and disclosure of contractual agreements that limit such access for investigators	**12**
Ancillary and post-trial care	30	Provisions, if any, for ancillary and post-trial care, and for compensation to those who suffer harm from trial participation	**N/A**
Dissemination policy	31a	Plans for investigators and sponsor to communicate trial results to participants, healthcare professionals, the public, and other relevant groups (eg, via publication, reporting in results databases, or other data sharing arrangements), including any publication restrictions	**12**
	31b	Authorship eligibility guidelines and any intended use of professional writers	**14**
	31c	Plans, if any, for granting public access to the full protocol, participant-level dataset, and statistical code	**12**
**Appendices**			
Informed consent materials	32	Model consent form and other related documentation given to participants and authorised surrogates	**Appendix 4**
Biological specimens	33	Plans for collection, laboratory evaluation, and storage of biological specimens for genetic or molecular analysis in the current trial and for future use in ancillary studies, if applicable	**Not provided**

*It is strongly recommended that this checklist be read in conjunction with the SPIRIT 2013 Explanation & Elaboration for important clarification on the items. Amendments to the protocol should be tracked and dated. The SPIRIT checklist is copyrighted by the SPIRIT Group under the Creative Commons "Attribution-NonCommercial-NoDerivs 3.0 Unported" license.

For peer review only - http://bmjopen.bmj.com/site/about/guidelines.xhtml

Appendix 2- PROMED-EX Trial Steering Committee (TSC)

Table S1: TSC members and their roles

Member	Role
**Prof Emma Stevenson**	Independent Chair
**Prof Jayne Woodside**	Dependant- scientific advisor
**Prof Dorothee Volkert**	Dependant- scientific advisor
**Prof Lisette de Groot**	Dependant- scientific advisor
**Prof Lorraine Brennan**	Dependant- scientific advisor
**Prof Chris Cardwell**	Dependant- statistician
**Ms Bernie Devlin**	Independent lay member
**Ms Eleanor Butterwick**	Independent lay member
**Ms Clare Jess**	Facilitator
**Dr Roisin O'Neill/ Dr Dominic Farsi**	Dependant- Trial manager
**Dr Claire McEvoy**	Dependant- PROMED-EX principal investigator

Table S2: Roles and responsibilities of the TSC

**Roles and responsibilities**	
Aims of the TSC	To act as the oversight body for PROMED-EX on behalf of the Sponsor/Funder.
Terms of reference	The role of the TSC is to provide oversight for the trial [Medical Research Council (MRC) Good Clinical Practice (GCP) guidelines (1998)]. It should also provide advice through its independent Chairperson to the PI, local trial team, BBSRC on all aspects of the trial.
Specific roles of TSC	• provide expert oversight of the trial<br>• maintain confidentiality of all trial information that is not already in the public domain<br>• make decisions as to the future continuation (or otherwise) of the trial/s<br>• monitor recruitment rates and encourage the trial team to develop strategies to deal with any recruitment problems<br>• review regular reports of the trial from the trial team.<br>• assess the impact and relevance of any accumulating external evidence<br>• monitor completion of CRFs and comment on strategies from trial team to encourage satisfactory completion in the future<br>• monitor follow-up rates and review strategies from Trial team to deal with problems<br>• approve any amendments to the protocol, where appropriate<br>• approve any proposals by the Trial team concerning any change to the design of the trial, including additional substudies<br>• oversee the timely reporting of trial results<br>• comment on the main trial manuscript<br>• comment on any abstracts and presentations of any results *during* the running of the trial

For peer review only

Study no ___________ Date___________

PROMED DIET SCORE

Visit: Baseline ☐ 3 mon ☐ 6 mon ☐

Questions	Self-reported	Criteria for 1 point	Point?
1. Do you use olive oil or rapeseed oil as your main cooking fat?		Yes	
2. How much olive/ rapeseed oil do you consume in a given day? Including oil used for frying, salads, out-of-house meals etc (in tablespoons)<br><br>How much olive or rapeseed oil-based spread do you consume in a given day? (in teaspoons)		≥1 tbsp oil / day<br><br>**and/or**<br><br>3 tsp spread/ day	
3. How many servings of dairy foods (milk, cheese, yogurts) or plant proteins alternatives do you eat per day?		≥2 portions per day	
4. How many portions of fruit (including natural fruit juices) do you consume per day? (1 portion = 1 apple/banana (80g), small glass juice (150ml)		≥2 portions/ day	
5. How many vegetable servings do you consume per day? Including raw/ cooked vegetables, salad but not including potatoes (1 serving: 3 tablespoons/80g)		≥3 portions/ day	
6. How many servings of legumes (peas, beans and lentils including kidney beans, baked beans, chickpeas, red lentils etc) do you consume per week? (1 serving :3 tablespoons/ 80g)		≥3 servings/ week	
7. How many servings of red meat including beef, pork, lamb and minced beef do you consume per week? (1 serving: medium portion/ 100–150 g)		≤2 servings /week	
8. How many servings of processed meat including ham, bacon, sausages, meat pies and other meat products etc.) do you consume per week? (1 serving: medium portion/ 100–150 g)		≤1 serving /week	
9. How many servings of chicken/ turkey do you consume per week? (1 serving: medium portion/ 100-150g)		≥2 servings /week	
10. How many servings of fish (tuna, cod, haddock, salmon, mackerel, herring, and sardines etc, including tinned varieties, excluding crumbed or battered fish) or shellfish do you consume per week? (1 serving: 1 fillet/small fish or 140g)		≥2 servings /week	
11. Do you preferentially consume wholegrain bread and/ or cereal and/ or rice and/ or pasta instead of non-wholegrain (white) varieties?		Yes	
12. How many servings of natural nuts do you consume per week? (1 serving: 1 small handful/ 30 g)		≥3 servings /week	
13. How many times per week do you consume sweet foods (including biscuits, buns, pastries, chocolate, sweets, sweet or carbonated beverages or desserts)?		≤3 times/ week	
14. How often would you consume up to 3 small glasses of wine or equivalent other alcoholic beverages per week? (1 small glass:125ml)		1-3 glasses or equivalent ≥ 3 days/ week	

For peer review only

Study Protocol: Randomised controlled trial to evaluate the effects of a PROtein enriched MEDiterranean Diet and EXercise on nutritional status and cognition in undernourished adults with subjective cognitive decline: The PROMED-EX Study.

Journal:	*BMJ Open*
Manuscript ID	bmjopen-2022-070689.R1
Article Type:	Protocol
Date Submitted by the Author:	14-Apr-2023
Complete List of Authors:	Ward, Nicola Ann; Queen's University Belfast, Centre for Public Health<br>Reid-McCann, Rachel; Queen's University Belfast, Centre for Public Health<br>Brennan, Lorraine; University College Dublin School of Agriculture and Food Science, Institute of Food and Health and Conway Institute<br>Cardwell, Christopher; Queen's University Belfast, Centre for Public Health<br>de Groot , LC; Wageningen University, Division of Human Nutrition<br>Maggi, Stefania; Neuroscience Research Institute National Research Council Padua Branch, Aging Branch<br>McCaffrey, Noel; c/o Irish Wheelchair Association, ExWell Medical<br>McGuinness, Bernadette; Queen's University Belfast, Centre for Public Health<br>McKinley, Michelle C.; Queen's University Belfast, Centre for Public Health<br>Noale, M; Neuroscience Research Institute National Research Council Padua Branch, Aging Branch<br>O'Neill, Roisin; Queen's University Belfast, Centre for Public Health<br>Prinelli, Federica; Institute for Biomedical Technologies National Research Council, Epidemiology Unit<br>Sergi, Giuseppe; University of Ferrara, Department of Medical Sciences<br>Trevisan, Caterina.; University of Ferrara Department of Medical Sciences, Department of Medical Sciences<br>Volkert, Dorothee; Friedrich-Alexander-Universitat Erlangen-Nurnberg, Institute for Biomedicine of Aging<br>Woodside, Jayne; Queen's University Belfast, Centre for Public Health<br>McEvoy, Claire T.; Queen's University Belfast, Centre for Public Health; Global Brain Health Institute, Trinity College Dublin, Ireland & University of California San Francisco, USA
**Primary Subject Heading**:	Public health
Secondary Subject Heading:	Neurology, Public health, Sports and exercise medicine, Nutrition and metabolism
Keywords:	Nutrition < TROPICAL MEDICINE, Dementia < NEUROLOGY, NUTRITION

	& DIETETICS, PUBLIC HEALTH, PREVENTIVE MEDICINE

SCHOLARONE™
Manuscripts

Study Protocol: Randomised controlled trial to evaluate the effects of a PROtein enriched MEDiterranean Diet and EXercise on nutritional status and cognition in undernourished adults with subjective cognitive decline: The PROMED-EX Study.

Ward N[1], Reid-McCann R[1], Brennan L[2], Cardwell C[1], de Groot CPGM[3], Maggi S[4], McCaffrey N[5], McGuinness B[1], McKinley MC[1], Noale M[4], O'Neill RF[1], Prinelli F[6], Sergi G[7], Trevisan C[7], Volkert D[8], Woodside JV[1], McEvoy CT[1,9] and the PROMED-COG consortium.

[1]Centre for Public Health, Queen's University Belfast, Belfast. Northern Ireland. UK.

[2]School of Agriculture and Food Science, Institute of Food and Health and Conway Institute, University College Dublin, Dublin. Ireland.

[3]Division of Human Nutrition, Wageningen University, Wageningen. Netherlands

[4] Neuroscience Institute, Aging Branch, National Research Council (CNR), Padua. Italy.

[5]ExWell Medical, c/o Irish Wheelchair Association, Dublin. Ireland.

[6] Institute of Biomedical Technologies, Epidemiology Unit, National Research Council (CNR), Segrate (MI). Italy

[7]Department of Medical Sciences, University of Ferrara, Ferrara. Italy

[8]Institute for Biomedicine of Aging, Friedrich-Alexander-Universität Erlangen-Nürnberg, Nuremberg. Germany.

[9]The Global Brain Institute, Trinity College Dublin, Ireland & University of California San Francisco, USA.

Corresponding Author: Dr Claire McEvoy, Centre for Public Health, Institute of Clinical Sciences building A, Queen's University Belfast, Grosvenor Road, Belfast. BT12 6BJ. Northern Ireland. UK. Email: **c.mcevoy@qub.ac.uk**

Word count, excluding title page, abstract, references, figures, and table

Key Words: nutrition, exercise, cognitive function, dementia

For peer review only - http://bmjopen.bmj.com/site/about/guidelines.xhtml

ABSTRACT

Introduction Undernutrition leading to unplanned weight loss is common in older age and has been linked to increased dementia risk in later life. Weight loss can precede dementia by a decade or more, providing a unique opportunity for early intervention to correct undernutrition and potentially prevent or delay cognitive impairment. The combined effects of diet and exercise on undernutrition have not yet been evaluated. The objective of the PROMED-EX trial is to determine the effect of a protein-enriched Mediterranean Diet, with and without exercise, on nutritional status and cognitive performance in older adults at risk of undernutrition and cognitive decline.

Methods One hundred and five participants aged 60 years and over at risk of undernutrition and with subjective cognitive decline will be recruited to participate in a 6-month, single-blind, parallel-group randomised controlled trial. Participants will be block randomised into one of three groups: Group 1. PROMED-EX (diet + exercise), Group 2. PROMED (diet only), and Group 3. Standard Care (control). The primary outcome is nutritional status measured using the Mini Nutritional Assessment (MNA). Secondary outcomes include cognitive function, nutritional intake, body composition, physical function, and quality of life. Mechanistic pathways for potential diet and exercise-induced change in nutritional status and cognition will be explored by measuring inflammatory, metabolic, nutritional and metabolomic biomarkers.

Ethics and dissemination The study is approved by the UK Office for Research Ethics Committee (Ref 21/NW/0215). Written informed consent will be obtained from participants prior to recruitment. Research results will be disseminated to the public via meetings and media and the scientific community through conference presentations and publication in academic journals.

Trial registration NCT 05166564.

Strengths and limitations of this study

- PROMED-EX is the first randomised controlled trial designed to evaluate the effect of an optimised protein-enriched Mediterranean diet, with and without exercise, on nutritional status and cognitive performance in a high-risk population.
- The integration of state-of-the-art metabolomics, metabolic, inflammatory, and nutritional biomarkers will help elucidate biological mechanisms underlying how diet can influence nutrition status and cognition and how exercise can interact with nutrition to modify these processes.
- A Personal and Public Involvement (PPI) group will contribute at all stages of the PROMED-EX trial to optimise the research design and dissemination strategy of the research findings.
- The nature of the 'real world' pragmatic study design means that it is not possible to blind participants to treatment allocation, possibly leading to performance bias.
- Data will be analysed by a blinded member of the study team to minimise detection bias.

For peer review only - http://bmjopen.bmj.com/site/about/guidelines.xhtml

INTRODUCTION

Undernutrition, defined as insufficient food and/or nutrient intake that results in altered body composition and unplanned weight loss,[1] is common in older age and affects >1.3 million older adults in the UK.[2] Individuals with cognitive impairment are particularly vulnerable to undernutrition and its many adverse consequences. Undernourished people with dementia experience more rapid decline in cognitive and physical functions, increased neuropsychiatric symptoms, and greater risk of hospitalisation and mortality, than those with adequate nutritional status.[3,4] Being undernourished has also emerged as a potentially modifiable risk factor for dementia.[5] In prospective studies, weight loss has been associated with increased dementia risk,[6] and precedes the onset of dementia by a decade or more,[7,8] making it an intervention target that should be prioritised.

Causes of undernutrition are increased nutrient requirements, inadequate dietary intake and reduced nutrient bioavailability,[9] but the exact mechanisms of undernutrition in dementia are not clear. It is possible that weight loss may be both a consequence and cause of neurodegeneration and dementia. Loss of muscle and weight in older adults has been linked to accelerated decline in brain structures,[10-12] even after exclusion of cognitive impairment,[12] suggesting a pathophysiological role of undernutrition in dementia. In contrast, weight loss could be an early marker of dementia development. Pathological changes in the brain are present 10-15 years prior to the clinical symptoms of cognitive impairment and induce inflammatory[13] and physiological changes beyond normal ageing that can have a negative impact on nutrition and body weight. Dementia pathology affects brain regions such as the hypothalamus which is important for appetite regulation and eating behaviour.[14] This may explain alterations of leptin and ghrelin metabolic signals that are observed in early disease.[15] Early brain changes can also affect chemosensory perception including taste and olfactory function,[16] as well as satiety regulation, protein and lipid synthesis,[17] and food preferences. Disease progression and worsening cognitive impairment can cause forgetfulness to eat, dysphagia,[18] sleep disturbance[19] and social withdrawal,[20] which can all have adverse effects on food intake.[21]

To date, most studies have focused on the impact of diet on physical function,[22] with limited number of interventions targeting undernutrition for cognitive benefit. Short-term treatment of undernutrition has been shown to improve both nutritional status and cognitive performance.[23-25] However, small sample sizes as well as differences in residential status of study populations and nutritional interventions (dietetic counselling,[23] oral supplementation,[24,25] meal provision[25]), and cognitive outcomes make it difficult to know whether addressing undernutrition could protect against cognitive decline. Hence, trials are warranted to test the efficacy of intervention for undernutrition in populations at increased risk of cognitive decline but not yet cognitively impaired and may benefit from early intervention.

The Mediterranean Diet (MedDiet) is a nutrient dense dietary pattern with proven anti-inflammatory benefits[26] and may offer protection against cognitive decline during ageing. Observational data have reported links between the MedDiet and favourable changes in brain structures and functions,[27] slower rate of cognitive decline[28,29] and decreased dementia risk, [30,31] which appear to be attributable to the combined effect of different foods rather than the action of a single food or nutrient.[32] Furthermore, the MedDiet is associated with nutritional adequacy and lower risk of undernutrition and micronutrient deficiency.[33] Adequate protein (1.0-1.2/kg body weight/day)[34] and energy intake[35] are critical for preventing muscle loss[36] and undernutrition in older adults.[37] However, emerging data

For peer review only - http://bmjopen.bmj.com/site/about/guidelines.xhtml

suggest that a high-quality dietary pattern is also important,[38] lending support to a 'whole diet' approach for preventing muscle loss and functional decline during ageing.

Physical exercise has positive effects on cognition,[39] especially in domains of executive function and processing speed,[40] by increasing neurogenesis and upregulating neurotrophic factors.[41] However, it is not known how diet and exercise interact for neuroprotection. Multi-domain interventions that include both a healthy diet and exercise appear to elicit stronger positive results for cognitive performance than each intervention in isolation.[42] Furthermore, exercise can stimulate protein synthesis[43] and enhance muscle mass and functional performance in older adults,[44] making it an attractive target for preventing undernutrition. An optimal protein MedDiet may induce additive effects on nutritional status and cognition when combined with exercise, but this has not yet been evaluated.

The PROtein enriched MEDiterranean Diet and EXercise (PROMED-EX) randomised controlled trial (RCT) aims to determine the effects of a protein-enriched MedDiet, with and without exercise, in comparison to standard care, on nutritional status in adults at risk of undernutrition and cognitive decline. PROMED-EX is part of the European "PROMED-COG" project, funded under the Horizon Joint Programming Initiative ‘a healthy diet for a healthy life’ to establish evidence for the balance between diet and physical activity for preventing undernutrition and promoting healthy neurocognitive ageing.[45]

METHODS AND ANALYSIS

PROMED-EX is a 6-month, single-blind, parallel-group RCT aiming to recruit 105 eligible adults residing in Northern Ireland (NI), aged 60 years+ and at risk of both undernutrition and cognitive decline. The protocol has been written in accordance with the Standard Protocol Items: Recommendations for Interventional Trials (SPIRIT) guidelines,[46] and a summary is reported using the checklist (Appendix 1). An independent Trial Steering Committee (TSC) has been established (Appendix 2) to oversee the conduct of the study.

Patient and public involvement (PPI)

A patient and public involvement (PPI) group (n=8) comprising older adults residing in NI with lived experience of subjective cognitive decline (SCD) and/or poor nutritional status were recruited for PROMED-EX. PPI members have contributed to the intervention design and co-development of materials, including lay summary, participant information sheet and consent form, advertisement materials, written educational resources for the interventions. Besides the trial design, two PPI members sit on the TSC and will input to future stages including implementation, analysis, and dissemination.

Study Design

Study participants will be randomised into one of three groups: Group 1. PROMED-EX: Protein-enriched MedDiet and exercise intervention (n=35); Group 2. PROMED: Protein-enriched MedDiet intervention (n=35); or Group 3. Control: Standard care healthy eating diet sheet (n=35) as shown in Figure 1. Participants will be informed of their allocated group after baseline data has been collected. Those randomised to the PROMED-EX and PROMED interventions will receive active support to adopt the behavioural targets in the first 3 months, after which time they will continue with self-directed maintenance of the newly adopted behaviours for a further 3 months.

Sample Size

For peer review only - http://bmjopen.bmj.com/site/about/guidelines.xhtml

As no prior studies have evaluated the effects of whole diet and exercise on nutritional status and cognition, the sample size calculation is based on a standard deviation of the change in MNA score of 4 points over a similar period from the control group of a previous nutrition intervention.[47] A total of 28 completing in each intervention and control group would provide over 80% power to detect a difference in the three means, based upon a statistically significant ANOVA result at the 5% level, hypothesising a true increase in MNA of 3 or more in both intervention groups, but not the control group. Loss to follow-up is estimated from our prior experience of conducting lifestyle trials in older adults[48-50] where attrition ranges from 6%[50] to 30%.[49] Considering, FINGER[42] evaluated an intensive multidomain lifestyle intervention among older adults at increased dementia risk and reported 12% withdrawal over 2 years. We anticipate dropout at 20% and therefore have added 20% to the sample size to account for non-compliance, dropout, or discontinuation.

Recruitment and Eligibility of Study Participants

Multiple recruitment strategies will be employed including paid Facebook advertisements, other media (e.g., newspaper, radio, and television), and identification of potentially eligible participants from community networks, primary care sectors and health and social care trusts in NI. Those interested in participating will be provided with an information sheet detailing the study protocol, followed by a phone call to discuss study procedures.

Potentially eligible participants will be screened by telephone/video to last around 45 minutes. Individuals will be eligible to enrol if they are community-dwelling, aged ≥60 years, at risk of undernutrition defined by a Mini Nutritional Assessment -Short Form (MNA-SF) score of 8-11[51] and have a positive screen for SCD.[52] The screening assessment includes the Telephone cognitive screen (T-CogS) or Mini Mental State Examination (MMSE) to exclude participants with cognitive impairment and a health and medical history questionnaire to determine if the participant has any medical conditions that compromise their ability to take part. This includes if they receive nutritional support, have a diagnosis of mild cognitive impairment or dementia, or dietary restrictions as shown in Table 1.

Randomisation, blinding and allocation

A study statistician independent of recruitment, intervention delivery and data collection will perform block randomisation using a computer-generated random number sequence in blocks of size 6 (using command RALLOC in STATA Release 16, StataCorp LP, College Station). Allocations will be prepared in sealed opaque envelopes by an independent study administrator and will not be revealed to participants until completion of baseline data collection. It will not be possible to blind the participant nor study researchers involved in intervention delivery to the allocated intervention group; however, data collection and analysis will be carried out by investigators blinded to treatment allocation.

Table 1: PROMED-EX Trial eligibility criteria

Inclusion Criteria	Exclusion Criteria

For peer review only - http://bmjopen.bmj.com/site/about/guidelines.xhtml

• Age ≥ 60 years<br>• Community-dwelling<br>• Mini Nutrition Assessment-Short Form (MNA-SF) score 8-11, indicating increased risk of undernutrition[51]<br><br>• Positive screen for subjective cognitive decline (SCD)[52] defined as a 'yes' response to the question: *"During the past 12 months, have you experienced confusion or slight memory loss that is happening more often or is getting worse?"*<br><br>• Ability to provide informed consent.	• Cognitive impairment: Telephone cognitive screen (T-CogS) ≤*21* or Mini Mental State Examination (MMSE) ≤*24* or clinical diagnosis of mild cognitive impairment or dementia<br>• In receipt of prescribed oral or artificial nutritional support<br>• Dietary restrictions/allergies to PROMED foods (e.g. nuts) or taking high-dose nutritional (e.g. Vitamin E) that limit the ability to take part in the study<br>• Living in long-term care facilities<br>• Self-reported:<br>○ Dysphagia<br>○ Chronic Kidney disease<br>○ Diabetes complications or HbA1c >8%<br>○ Significant medical comorbidities that may alter cognitive test performance, e.g., stroke, brain injury<br>• Inability to complete cognitive testing due to language or visual impairment

Interventions

Group 1. PROMED-EX

Protein enriched MedDiet intervention

Participants will meet with a trained researcher within one week following randomisation to receive personalised dietary counselling based on their baseline anthropometry, MNA score and PROMED diet score (appendix 3). This session will last up to 60 minutes where education will be provided on adequate nutritional status, avoiding weight loss, and encouraging regular meal pattern. Participants will be encouraged to set dietary goals to adopt PROMED diet behaviours (shown in Box 1) to achieve overall intervention targets: (1) increased protein intake up to 1.5g per kg current body weight;[37] (2) increased MNA score by ≥2 points; and (3) increased PROMED diet score by ≥2 points. Daily energy requirements will be calculated at 30kcal/kg of body weight with a supplement of 400-600kcal only if weight gain is indicated.[53]

Box 1: PROMED dietary behaviours

- 1tbsp of olive oil per day
- 5 or more portions of fruit and vegetables per day (≥ 2 portions fruit and ≥3 portions vegetables per day)

For peer review only - http://bmjopen.bmj.com/site/about/guidelines.xhtml

- Preferential consumption of chicken and oily fish, ≥2 portions of each per week (and reduction of red meat <2).
- ≥2 portions per day of wholegrain foods (including wholemeal bread, wholegrain rice, whole wheat pasta)
- 3 handfuls of natural nuts (90g) per week
- ≥2 portions per day of dairy foods (including, milk, cheese, yoghurts) or plant protein alternatives
- Moderate alcohol (1-2 glasses of wine/beer per day consumed with meals) for alcohol consumers

Written materials (PROMED diet guide and recipe book) will be provided to support adoption of diet behaviours. Participants will be given a recipe book with suggested quick, low-cost menu plans and practical dietary tips to overcome small appetite. Key foods will be delivered to their home free of charge for the first 3-months to encourage adoption of the PROMED diet behaviours. Foods will be self-selected by participants from a pre-defined list to facilitate personal preference and will include olive oil, fruits and vegetables, wholegrain foods (cereals, breads, rice and pasta), nuts, nut butters, canned oily fish and low-fat dairy foods.

Exercise intervention

The exercise intervention has been adapted from a commercially available 'ExWell@home' group exercise rehabilitation programme that is acceptable to older adults and suggested to improve health outcomes in those with chronic disease.[54] The home-based exercise intervention comprises strength, aerobic, core stability and balance exercises available across four levels of ability: (1) chair based, (2) basic, (3) standard and (4) advanced. After the dietary education session, participants will receive a personal exercise prescription involving a written plan, demonstration of exercises suitable for their fitness level, and instruction on safety aspects to minimise injury risk. Baseline fitness level will be determined using measured blood pressure, pulse rate, short physical performance test results, handgrip strength test obtained from the baseline study visit, and their assessed performance in a 'basic level' class with the trained researcher.

Participants will be asked to report their perceived intensity of exertion using the Borg Rate of Perceived Exertion (RPE) scale (6-20),[55] aiming for "somewhat hard" within a range of 12-14 points. Participants will be instructed to increase or decrease the frequency and intensity of exercise over the first 4 weeks to meet the intervention target of two x 30–60-minute moderate intensity exercise sessions per week. Participants will receive additional supportive materials to achieve their exercise goals including pre-recorded exercise classes, a written home-based exercise resource, a pedometer, and exercise diary.

Group 2. PROMED

Participants will receive only the Protein enriched MedDiet intervention and will be advised to keep other lifestyle factors unchanged throughout the study period, including maintenance of their usual physical activity level.

Group 3. Standard care

Participants will receive a general healthy eating diet sheet provided at baseline. To encourage retention in the study, personalised dietary advice and written materials will be offered to control group participants upon completion of the final study assessment.

For peer review only - http://bmjopen.bmj.com/site/about/guidelines.xhtml

Maximising and monitoring compliance with the diet and exercise interventions

For study participants randomised to the PROMED-EX and PROMED interventions (Groups 1 and 2 respectively) several approaches will be used to facilitate behaviour change and encourage compliance with the diet and exercise goals. The initial education session will be scheduled at a convenient time for the participant and in a convenient location (either in a dedicated research facility, the participants own home or via remote video call). Partners or caregivers who are responsible for food preparation and/or shopping will also be invited to the education session if applicable. Furthermore, techniques demonstrated to support diet and exercise behaviour change have been incorporated into the intervention written materials and include 'instruction on how to perform behaviour', 'action planning', 'self-monitoring' and 'problem solving.'[56]

Participants will be encouraged to self-monitor their compliance in achieving the diet and exercise goals where applicable. The researcher will contact participants weekly via telephone during the first 3-months to review individual progress, monitor compliance and body weight, deal with any difficulties participants may be experiencing in meeting their goals and provide support and personalised feedback as required. Participants will also be able to contact the researcher at any time during the 6-month intervention period.

Data collection and Study outcomes

Participants will meet with a researcher at baseline and after 3- and 6-months to complete a study assessment lasting no longer than 2.5-hours. The assessment will take place in a dedicated research facility at Queen's University Belfast (QUB) or in the participant's home if preferred. A summary of the outcomes and data collection measures at study time-points are found in Table 2.

For peer review only

For peer review only - http://bmjopen.bmj.com/site/about/guidelines.xhtml

Table 2: Overview of study data collection variables and measures

Study outcomes	Data collection variables/ measures	Baseline	3-month	6-month
**Primary outcome**				
Nutritional status	Mini Nutrition Assessment (MNA®) long-form questionnaire[57]	□	□	□
**Secondary outcomes**				
Nutrition and body composition				
Nutritional intake	PROMED diet score questionnaire and 4-day food diary	□	□	□
Appetite	Simplified Nutrition Appetite Questionnaire (SNAQ)[58]	□	□	□
Body composition	Bioelectric Impedance Analysis of body fat and fat free mass (Body Stat 1500 MDD)	□	□	□
Anthropometry	Height (stadiometer) and Weight (digital scales)	□	□	□
Physical activity				
Physical activity	Recent Physical Activity Questionnaire (RPAQ)[59]	□	□	□
Cognitive function				
Cognitive function	Catch-Cog,[60] Trail Making Test A and B[61] and Delayed Recall Test[62]	□	□	□
Physical strength and function				
Functional capacity	Short Physical Performance Battery (SPPB) [63]	□	□	□
Muscle strength	Hand Grip Strength (Dynamometer)	□	□	□
Psychosocial				
Health-Related Quality of Life	SF-36 Health Survey[64] questionnaire	□	□	□
Depressive symptoms	Geriatric Depression Scale[65] questionnaire	□	□	□
Blood Pressure				
Blood Pressure	Clinic measured blood pressure (Omron Healthcare)	□	□	□
Biochemical Measures				
Blood biomarkers	Nutritional: vitamins A,E,C, carotenoids and fatty acids. cardiometabolic: lipid profile, HbA1c ,glucose and insulin. Inflammatory: hs-CRP, IL-6.	□	□	□
**Evaluation**				
Study evaluation	Questionnaire			□

For peer review only

For peer review only - http://bmjopen.bmj.com/site/about/guidelines.xhtml

Primary outcome

The primary study outcome is between-group difference in change in nutritional status at 6-months using the validated MNA score (MNA® 0-30).[57] The MNA is the most widely validated tool for detecting undernutrition risk in older adults[66] and is shown to respond to dietary intervention.[47]

Secondary outcomes

Nutrition and body composition

Dietary behaviour will be reported using the PROMED diet score (range 0-14) adapted from a validated Spanish Mediterranean diet score[67] to incorporate the higher protein food targets and tailored for a Northern European population. Higher scores indicate increased concordance with the PROMED diet. Overall dietary intake will be assessed using a 4-day prospective food diary. Participants will be instructed to record all items of food and beverages consumed over four consecutive days (two weekend days) at home and provide details of the portion size consumed, cooking methods and named brands. Data on self-reported appetite will also be collected using the 4-item Simplified Nutrition Appetite Questionnaire (SNAQ) validated for older community dwelling adults.[58] Lower SNAQ scores indicate poorer appetite. Body composition will be assessed using non-invasive bioelectrical impedance analysis (BodyStat 1500 MDD) to estimate fat and fat free mass.[68] Anthropometric measures of weight and height will also be taken using digital scales and a stadiometer, respectively.

Physical activity

Time spent on different intensity levels of physical activity will be self-reported using a validated Recent Physical Activity Questionnaire (RPAQ) across four domains: home, commuting, occupational activity (if appropriate), and leisure activity over the previous 4 weeks.[59]

Cognitive function

A brief neuropsychological test battery (NTB) will be administered by a trained researcher to capture cognitive domains that are shown to be sensitive to progression in early stages of memory decline: global cognition, executive functioning, episodic and working memory, information processing speed and sustained attention.[60] The NTB will include the Catch-Cog assessment[60] comprising (i) three Alzheimer's Disease Assessment Scale–Cognitive subscales (Word Recognition (score range 0-12), Word Recall (score range 0-10), and Orientation (score range 0-8)) to assess episodic memory (ii) the Controlled Oral Word Association Test (number of acceptable words in one minute); Category Fluency Test (number of animals named in one minute); (iii) Digit Symbol Substitution Test (total number of correct symbols in 90 seconds) and (iv) Digit Span Backward (score range 0-14) to assess to assess executive function and working memory. The Trail Making Test (A and B) will be used to assess information processing speed and attention.[61] A delayed recall test of recalled learned words after a 5-minute delay (score range 0–10) will be administered, with higher scores indicating better memory performance. Summary composite scores will be created for global cognition and domains of memory and executive function.

Physical strength and function

For peer review only - http://bmjopen.bmj.com/site/about/guidelines.xhtml

Lower extremity functional status will be measured using the Short Physical Performance Battery (SPPB).[63] The SPPB includes three timed tasks to assess walking speed, chair stand and standing balance, with scores ranging from 0 (worst performance) to 12 (best performance). Muscle (handgrip) strength will be measured with a calibrated dynamometer (Type) (kg) using a standard procedure.[48]

Psychosocial

Health-related Quality of Life (HR QoL) will be determined by using the Short Form-36 (SF-36) 36-item questionnaire[64] which measures eight domains of health status: physical functioning, role limitations due to physical issues, role limitation due to emotional problems, energy/fatigue, emotional well-being, social functioning, pain, and general health perception. SF-36 scores range from 0-100 with higher scores indicating better HR QoL.[64] Depressive symptoms will be determined using the 15-item Geriatric Depression Scale (GDS) – Short Form). GDS scores range from 0 to 15, with a score ≥5 suggesting depression.[65]

Blood pressure

Systolic and diastolic blood pressure (mmHg) will be measured three times in the dominant arm after a 5-minute rest in the seated position using a calibrated automated device approved by the British Hypertension Society (Omron Healthcare). The first measure will be discarded and the average of the final two measurements will be recorded.

Biochemical measures

A 50 ml fasting peripheral venous blood sample will be taken at the start of each study assessment for metabolomic profiling and biomarker measurement. Anonymised samples will be processed in the Nutrition laboratory at QUB according to standardised protocols and stored in aliquots at -80°C until analysis. Biochemical markers relating to nutrition (vitamins A, C, E, carotenoids, and fatty acids), metabolic (lipid profile, HbA1c, glucose and insulin), and inflammation (hs-CRP, IL-6) will be measured. Plasma samples will be transferred to University College Dublin for metabolomics analysis.

Evaluation

A questionnaire will be administered at the end of the study to determine acceptability and tolerance of the intervention and the overall experience of taking part in the study. Participants will be asked to return diet and exercise logs to evaluate engagement with the intervention.

Data management and statistical analysis plan

Data will be de-identified and stored securely according to General Data Protection Regulation (GDPR) and UK Data Protection Act 2018 in QUB. There are no plans for interim study analysis. Participant characteristics by allocated group will be compared using analysis of variance for continuous variables and Chi square test for dichotomous variables. Skewed variables will be log transformed prior to analysis. An intention-to-treat analysis will be conducted using multiple linear regression model to compare MNA score at 6 months between the three groups adjusting for baseline. Dunnett's procedure will then be used to compare each intervention group to the controls, accounting for multiple testing. Analysis of secondary outcomes will utilise a similar method and these exploratory analyses will be

For peer review only - http://bmjopen.bmj.com/site/about/guidelines.xhtml

interpreted cautiously. Sensitivity analyses will be conducted to evaluate the diet and exercise behavioural changes maintained over 6-months and to determine the effect of intervention compliance on study-end-points. Multiple imputations for missing data will be used if required. Analyses will be performed using STATA Statistical Software: StataCorp LLC.

Ethics and dissemination

Ethical approval has been received from Office for Research Ethics Committee (OREC) (Ref 21/NW/0215) and the trial has been registered on ClinicalTrials.gov (NCT 05166564). The study will be reported according to CONSORT guidelines for parallel group randomised trials.[69] Written informed consent will be obtained from all study participants prior to study enrolment and any adverse events will be reported to the study sponsor (QUB) and OREC.

Study findings will be of interest to a range of stakeholders in the areas of healthy ageing, preventable malnutrition, cognitive decline, and dementia risk reduction, including academics, the public, healthcare professionals and clinicians, charitable organisations, and industry. Findings will be published in academic journals and published in open access repositories, for example the JPI-HDHL Meta Database (http://www.jpihdhl.eu) knowledge transfer platform.

COVID-19

The research team will comply with public health and QUB guidance regarding COVID-19. Several strategies will be implemented to mitigate COVID-19 risk: (i) COVID-19 risk screening for participants prior to study visits (ii) optional home study visits, (iii) optional remote delivery of intervention support, (iv) personal protective equipment for researchers conducting study assessments, (v) participant face coverings for study visits where possible, (vi) social distancing measures followed where possible, and (vii) researcher lateral flow testing pre-study visit.

DISCUSSION

To our knowledge, PROMED-EX is the first study aimed to evaluate the combined effects of a protein-enriched MedDiet and exercise on nutritional status and cognition in older adults at risk of undernutrition with SCD. Lifestyle interventions to address modifiable risk factors for dementia are likely to have a more significant public health impact when implemented at an early stage of disease and in high-risk populations. PROMED-EX will generate new knowledge about how a targeted lifestyle intervention delivered in a 'real-world' setting can impact nutritional and cognitive status among older individuals at risk of undernutrition and cognitive decline. A further novel aspect of the study is the planned evaluation that will provide insight into lifestyle behaviour changes that are feasible and acceptable for the intended population.

A key strength is the inclusion of a validated neurocognitive test battery in the current study allowing a comprehensive assessment of performance in both memory and non-memory domains of cognition cognition as used in other multidomain interventions in adults at increased dementia risk [42]. Prior studies in undernourished patients have relied on the limited MMSE global cognition to measure cognitive performance.[25] While MMSE is a

For peer review only - http://bmjopen.bmj.com/site/about/guidelines.xhtml

validated screener for cognitive impairment, it provides limited examination of visuospatial cognitive ability, long-term memory, and language functions. It also has a “ceiling” effect whereby individuals can still score quite highly even though they may have significant cognitive impairment.[70]

A further strength is the integration of state-of-the-art metabolomics, metabolic, inflammatory, and nutritional biomarkers in the study will help to elucidate biological mechanisms underlying how diet can influence nutrition status and cognition, and how exercise may act to modify these processes. Furthermore, the interventions will be delivered using a personalised approach with incorporation of evidence-based techniques to support and encourage behaviour change. Study participants will also benefit from written educational resources that have been co-designed with PPI members to support the desired diet and exercise behaviour changes. The ongoing collaboration with PPI members in the trial will undoubtedly optimise the dissemination of study findings.

There are some limitations to the study design that should be considered. The optimum period for lifestyle-induced change in nutritional status is not clear, and the duration of the current study intervention is shorter than in other multidomain trials;[42] however, improvement in MNA score in response to nutrition intervention has been observed in 12 weeks.[63] Furthermore, 6 months should be sufficient duration to detect increases in nutrient biomarkers in response to the interventions, especially in a high-risk population. The pragmatic study design also means that we may have limited power to detect between intervention arm effects on MNA. Furthermore, participants will not be blinded to treatment allocation, consistent with other lifestyle interventions. However, data will be analysed by a blinded member of the study team to minimise detection bias.

Conclusion

PROMED-EX focuses on undernutrition and cognitive impairment which are major contributors to mortality and disability in older age. The findings will provide new and critical insight into how modifications of diet and exercise behaviour can act to prevent undernutrition in older citizens at risk of undernutrition and cognitive decline. The study will advance understanding of how the protein enriched MedDiet influences nutritional status and cognitive performance, and whether biological pathways are altered by the addition of exercise. We expect the study findings to inform future dietary guidelines for prevention of undernutrition to promote healthy neurocognitive ageing, and guide the design of future larger trials to test the efficacy of diet and exercise on cognition.

Trial status:

Ongoing: Recruitment began in January 2022 and will complete in July 2023. Data collection is scheduled to complete in December 2023.

Contributions:

CME, LB, CC, FP, LdG, BMG, MMK, JVW, DV contributed to the conception and design of the trial. CME and NW drafted the manuscript. All authors (CME, NW, RRM, LB, CC, LdG, SM, NMC, BMG , MMK, MN, RON, FP, GS,CT, DV, JVW) contributed to the editing of manuscript and revising it critically for intellectual content. The Trial Steering Committee

For peer review only - http://bmjopen.bmj.com/site/about/guidelines.xhtml

approved this manuscript submission. CME gave final manuscript approval and is the corresponding and senior author.

Sponsor: The study sponsor is Queen’s University Belfast (Ref: B21/16) in accordance with UK Policy Framework for Health and Social Care.

Funding

This work is supported by funding from national funding organisations (listed below) under the umbrella of the European Joint Programming Initiative "A Healthy Diet for a Healthy Life" (JPI HDHL) and of the ERA NET Cofund ERA HDHL (GA N 696295 of the EU Horizon 2020 Research and Innovation Programme).

UK. Research & Innovation (UKRI): Biotechnology and Biological Sciences Research Council (BBSRC) and Medical Research Council (MRC) - Queen's University Belfast

Ministry of Universities and Research (MUR) Italy - National Research Council (CNR), Institute of Biomedical Technologies, Epidemiology Unit and Neuroscience Institute, Aging Branch; and University of Padova Geriatric Unit, Department of Medicine.

Health Research Board (HRB) Ireland - University College Dublin (UCD), Institute of Food and Health.

Funders and study sponsors had no input in any aspect of study design; writing of the protocol; and the decision to submit the protocol for publication.

Competing interests none declared

Ethics approval

Office for Research Ethics Committee (OREC).

Protocol Version 3: Any amendments will be reviewed and approved by Trial Steering Committee and OREC prior to implementation.

For peer review only

REFERENCES

1. Cederholm T, Bosaeus I, Barazzoni R, et al. Diagnostic criteria for malnutrition – An ESPEN Consensus Statement. Clinical Nutrition 2015;34(3):335-40. doi: 10.1016/j.clnu.2015.03.001
2. Stratton RJ Smith T, Gabe S. Managing malnutrition to improve lives and save money. BAPEN, 2018.
3. Sanders CL, Wengreen HJ, Schwartz S, et al. Nutritional Status is Associated With Severe Dementia and Mortality: The Cache County Dementia Progression Study. Alzheimer Dis Assoc Disord 2018;32(4):298-304. doi: 10.1097/wad.0000000000000274
4. Guerin O, Soto ME, Brocker P, et al. Nutritional status assessment during Alzheimer's disease: results after one year (the REAL French Study Group). J Nutr Health Aging 2005;9(2):81-4.
5. Qizilbash N, Gregson J, Johnson ME, et al. BMI and risk of dementia in two million people over two decades: a retrospective cohort study. Lancet Diabetes Endocrinol 2015;3(6):431-36. doi: 10.1016/s2213-8587(15)00033-9 [published Online First: 20150409]
6. Lee CM, Woodward M, Batty GD, et al. Association of anthropometry and weight change with risk of dementia and its major subtypes: A meta-analysis consisting 2.8 million adults with 57 294 cases of dementia. Obes Rev 2020;21(4):e12989. doi: 10.1111/obr.12989 [published Online First: 20200103]
7. Knopman DS, Edland SD, Cha RH, et al. Incident dementia in women is preceded by weight loss by at least a decade. Neurology 2007;69(8):739-46. doi: 10.1212/01.wnl.0000267661.65586.33
8. LeBlanc ES, Rizzo JH, Pedula KL, et al. Weight Trajectory over 20 Years and Likelihood of Mild Cognitive Impairment or Dementia Among Older Women. J Am Geriatr Soc 2017;65(3):511-19. doi: 10.1111/jgs.14552 [published Online First: 20161219]
9. Volkert D, Kiesswetter E, Cederholm T, et al. Development of a Model on Determinants of Malnutrition in Aged Persons: A MaNuEL Project. Gerontol Geriatr Med 2019;5:2333721419858438-38. doi: 10.1177/2333721419858438
10. Burns JM, Johnson DK, Watts A, et al. Reduced lean mass in early Alzheimer disease and its association with brain atrophy. Arch Neurol 2010;67(4):428-33. doi: 10.1001/archneurol.2010.38
11. Yu JH, Kim REY, Jung JM, et al. Sarcopenia is associated with decreased gray matter volume in the parietal lobe: a longitudinal cohort study. BMC Geriatr 2021;21(1):622. doi: 10.1186/s12877-021-02581-4 [published Online First: 20211102]
12. Jimenez A, Pegueroles J, Carmona-Iragui M, et al. Weight loss in the healthy elderly might be a non-cognitive sign of preclinical Alzheimer's disease. Oncotarget 2017;8(62):104706-16. doi: 10.18632/oncotarget.22218 [published Online First: 20171031]
13. McGrattan AM, McGuinness B, McKinley MC, et al. Diet and Inflammation in Cognitive Ageing and Alzheimer's Disease. Curr Nutr Rep 2019;8(2):53-65. doi: 10.1007/s13668-019-0271-4
14. Ahima RS, Antwi DA. Brain regulation of appetite and satiety. Endocrinol Metab Clin North Am 2008;37(4):811-23. doi: 10.1016/j.ecl.2008.08.005
15. Kimura A, Sugimoto T, Niida S, et al. Association Between Appetite and Sarcopenia in Patients With Mild Cognitive Impairment and Early-Stage Alzheimer's Disease: A Case-Control Study. Front Nutr 2018;5:128. doi: 10.3389/fnut.2018.00128 [published Online First: 20181218]
16. Attems J, Walker L, Jellinger KA. Olfaction and Aging: A Mini-Review. Gerontology 2015;61(6):485-90. doi: 10.1159/000381619 [published Online First: 20150509]

For peer review only - http://bmjopen.bmj.com/site/about/guidelines.xhtml

17. Doorduijn AS, van de Rest O, van der Flier WM, et al. Energy and Protein Intake of Alzheimer's Disease Patients Compared to Cognitively Normal Controls: Systematic Review. J Am Med Dir Assoc 2019;20(1):14-21. doi: 10.1016/j.jamda.2018.06.019 [published Online First: 20180809]
18. Alagiakrishnan K, Bhanji RA, Kurian M. Evaluation and management of oropharyngeal dysphagia in different types of dementia: A systematic review. Archives of Gerontology and Geriatrics 2013;56(1):1-9. doi: https://doi.org/10.1016/j.archger.2012.04.011
19. Hiller AJ, Ishii M. Disorders of Body Weight, Sleep and Circadian Rhythm as Manifestations of Hypothalamic Dysfunction in Alzheimer's Disease. Front Cell Neurosci 2018;12:471. doi: 10.3389/fncel.2018.00471 [published Online First: 20181205]
20. Honda Y, Meguro K, Meguro M, et al. Social withdrawal of persons with vascular dementia associated with disturbance of basic daily activities, apathy, and impaired social judgment. Care Manag J 2013;14(2):108-13. doi: 10.1891/1521-0987.14.2.108
21. Volkert D, Chourdakis M, Faxen-Irving G, et al. ESPEN guidelines on nutrition in dementia. Clin Nutr 2015;34(6):1052-73. doi: 10.1016/j.clnu.2015.09.004 [published Online First: 20150925]
22. Reinders I, Wijnhoven HAH, Jyväkorpi SK, et al. Effectiveness and cost-effectiveness of personalised dietary advice aiming at increasing protein intake on physical functioning in community-dwelling older adults with lower habitual protein intake: rationale and design of the PROMISS randomised controlled trial. BMJ Open 2020;10(11):e040637. doi: 10.1136/bmjopen-2020-040637 [published Online First: 20201120]
23. Endevelt R, Lemberger J, Bregman J, et al. Intensive dietary intervention by a dietitian as a case manager among community dwelling older adults: the EDIT study. J Nutr Health Aging 2011;15(8):624-30. doi: 10.1007/s12603-011-0074-9
24. Wouters-Wesseling W, Vos AP, Van Hal M, et al. The effect of supplementation with an enriched drink on indices of immune function in frail elderly. J Nutr Health Aging 2005;9(4):281-6.
25. Blondal BS, Geirsdottir OG, Halldorsson TI, et al. HOMEFOOD randomised trial – Six-month nutrition therapy improves quality of life, self-rated health, cognitive function, and depression in older adults after hospital discharge. Clinical Nutrition ESPEN 2022;48:74-81. doi: https://doi.org/10.1016/j.clnesp.2022.01.010
26. Casas R, Sacanella E, Urpí-Sardà M, et al. Long-Term Immunomodulatory Effects of a Mediterranean Diet in Adults at High Risk of Cardiovascular Disease in the PREvención con DIeta MEDiterránea (PREDIMED) Randomized Controlled Trial. J Nutr 2016;146(9):1684-93. doi: 10.3945/jn.115.229476 [published Online First: 20160720]
27. Berti V, Walters M, Sterling J, et al. Mediterranean diet and 3-year Alzheimer brain biomarker changes in middle-aged adults. Neurology 2018;90(20):e1789-e98. doi: 10.1212/wnl.0000000000005527 [published Online First: 20180413]
28. McEvoy CT, Hoang T, Sidney S, et al. Dietary patterns during adulthood and cognitive performance in midlife: The CARDIA study. Neurology 2019;92(14):e1589-e99. doi: 10.1212/wnl.0000000000007243 [published Online First: 20190306]
29. van de Rest O, Berendsen AA, Haveman-Nies A, et al. Dietary patterns, cognitive decline, and dementia: a systematic review. Adv Nutr 2015;6(2):154-68. doi: 10.3945/an.114.007617
30. Limongi F, Siviero P, Bozanic A, et al. The Effect of Adherence to the Mediterranean Diet on Late-Life Cognitive Disorders: A Systematic Review. Journal of the American Medical Directors Association 2020;21(10):1402-09. doi: https://doi.org/10.1016/j.jamda.2020.08.020

For peer review only - http://bmjopen.bmj.com/site/about/guidelines.xhtml

31. Singh B, Parsaik AK, Mielke MM, et al. Association of mediterranean diet with mild cognitive impairment and Alzheimer's disease: a systematic review and meta-analysis. J Alzheimers Dis 2014;39(2):271-82. doi: 10.3233/jad-130830
32. Feart C, Samieri C, Barberger-Gateau P. Mediterranean diet and cognitive health: an update of available knowledge. Curr Opin Clin Nutr Metab Care 2015;18(1):51-62. doi: 10.1097/mco.0000000000000131
33. Castro-Quezada I, Román-Viñas B, Serra-Majem L. The Mediterranean diet and nutritional adequacy: a review. Nutrients 2014;6(1):231-48. doi: 10.3390/nu6010231
34. Kiesswetter E, Sieber CC, Volkert D. Protein intake in older people : Why, how much and how? Z Gerontol Geriatr 2020;53(4):285-89. doi: 10.1007/s00391-020-01723-4 [published Online First: 20200414]
35. Volkert D, Beck AM, Cederholm T, et al. ESPEN guideline on clinical nutrition and hydration in geriatrics. Clinical Nutrition 2019;38(1):10-47. doi: https://doi.org/10.1016/j.clnu.2018.05.024
36. Okamura T, Miki A, Hashimoto Y, et al. Shortage of energy intake rather than protein intake is associated with sarcopenia in elderly patients with type 2 diabetes: A cross-sectional study of the KAMOGAWA-DM cohort. J Diabetes 2019;11(6):477-83. doi: 10.1111/1753-0407.12874 [published Online First: 20181204]
37. Bauer J, Biolo G, Cederholm T, et al. Evidence-based recommendations for optimal dietary protein intake in older people: a position paper from the PROT-AGE Study Group. J Am Med Dir Assoc 2013;14(8):542-59. doi: 10.1016/j.jamda.2013.05.021 [published Online First: 20130716]
38. Granic A, Mendonça N, Sayer AA, et al. Effects of dietary patterns and low protein intake on sarcopenia risk in the very old: The Newcastle 85+ study. Clin Nutr 2020;39(1):166-73. doi: 10.1016/j.clnu.2019.01.009 [published Online First: 20190121]
39. Livingston G, Sommerlad A, Orgeta V, et al. Dementia prevention, intervention, and care. Lancet 2017;390(10113):2673-734. doi: 10.1016/s0140-6736(17)31363-6 [published Online First: 20170720]
40. Kirk-Sanchez NJ, McGough EL. Physical exercise and cognitive performance in the elderly: current perspectives. Clin Interv Aging 2014;9:51-62. doi: 10.2147/cia.S39506 [published Online First: 20131218]
41. Lista I, Sorrentino G. Biological Mechanisms of Physical Activity in Preventing Cognitive Decline. Cellular and Molecular Neurobiology 2010;30(4):493-503. doi: 10.1007/s10571-009-9488-x
42. Ngandu T, Lehtisalo J, Solomon A, et al. A 2 year multidomain intervention of diet, exercise, cognitive training, and vascular risk monitoring versus control to prevent cognitive decline in at-risk elderly people (FINGER): a randomised controlled trial. Lancet 2015;385(9984):2255-63. doi: 10.1016/s0140-6736(15)60461-5 [published Online First: 20150312]
43. Trommelen J, Betz MW, van Loon LJC. The Muscle Protein Synthetic Response to Meal Ingestion Following Resistance-Type Exercise. Sports Med 2019;49(2):185-97. doi: 10.1007/s40279-019-01053-5
44. McGlory C, van Vliet S, Stokes T, et al. The impact of exercise and nutrition on the regulation of skeletal muscle mass. J Physiol 2019;597(5):1251-58. doi: 10.1113/jp275443 [published Online First: 20180801]
45. O'Neill R, Brennan L, Prinelli F, et al. PROtein enriched MEDiterranean diet to combat undernutrition and promote healthy neuroCOGnitive ageing in older adults: The PROMED-COG consortium project. Nutrition Bulletin 2022 doi: 10.1111/nbu.12571

For peer review only - http://bmjopen.bmj.com/site/about/guidelines.xhtml

46. Chan A-W, Tetzlaff JM, Gøtzsche PC, et al. SPIRIT 2013 explanation and elaboration: guidance for protocols of clinical trials. BMJ : British Medical Journal 2013;346:e7586. doi: 10.1136/bmj.e7586
47. Luger E, Dorner TE, Haider S, et al. Effects of a Home-Based and Volunteer-Administered Physical Training, Nutritional, and Social Support Program on Malnutrition and Frailty in Older Persons: A Randomized Controlled Trial. J Am Med Dir Assoc 2016;17(7):671.e9-71.e16. doi: 10.1016/j.jamda.2016.04.018
48. Marseglia A, Xu W, Fratiglioni L, et al. Effect of the NU-AGE Diet on Cognitive Functioning in Older Adults: A Randomized Controlled Trial. Front Physiol 2018;9:349-49. doi: 10.3389/fphys.2018.00349
49. McEvoy CT, Moore SE, Appleton KM, et al. Trial to Encourage Adoption and Maintenance of a Mediterranean Diet (TEAM-MED): Protocol for a Randomised Feasibility Trial of a Peer Support Intervention for Dietary Behaviour Change in Adults at High Cardiovascular Disease Risk. Int J Environ Res Public Health 2018;15(6) doi: 10.3390/ijerph15061130 [published Online First: 20180531]
50. van de Rest O, van der Zwaluw NL, Tieland M, et al. Effect of resistance-type exercise training with or without protein supplementation on cognitive functioning in frail and pre-frail elderly: secondary analysis of a randomized, double-blind, placebo-controlled trial. Mech Ageing Dev 2014;136-137:85-93. doi: 10.1016/j.mad.2013.12.005 [published Online First: 20131227]
51. Kaiser MJ, Bauer JM, Ramsch C, et al. Validation of the Mini Nutritional Assessment short-form (MNA®-SF): A practical tool for identification of nutritional status. JNHA - The Journal of Nutrition, Health and Aging 2009;13(9):782. doi: 10.1007/s12603-009-0214-7
52. CDC. BRFSS Questionnaires. July ed: Centres for Disease Control and Prevention, 2020.
53. Excellence NNIfHaC. Nutrition support for adults: oral nutrition support, enteral tube feeding and parenteral nutrition. Clinical guideline CG32. ONLINE, 2017.
54. Skelly F. Community-based exercise rehabilitation in a diverse chronic disease population. Dublin City University, 2021.
55. Lea JWD, O'Driscoll JM, Hulbert S, et al. Convergent Validity of Ratings of Perceived Exertion During Resistance Exercise in Healthy Participants: A Systematic Review and Meta-Analysis. Sports Medicine - Open 2022;8(1):2. doi: 10.1186/s40798-021-00386-8
56. Lin H, Xu D, Yang M, et al. Behaviour change techniques that constitute effective planning interventions to improve physical activity and diet behaviour for people with chronic conditions: a systematic review. BMJ Open 2022;12(8):e058229. doi: 10.1136/bmjopen-2021-058229 [published Online First: 20220822]
57. Guigoz Y, Vellas B. Nutritional Assessment in Older Adults : MNA® 25 years of a Screening Tool and a Reference Standard for Care and Research; What Next? J Nutr Health Aging 2021;25(4):528-83. doi: 10.1007/s12603-021-1601-y
58. Wilson MM, Thomas DR, Rubenstein LZ, et al. Appetite assessment: simple appetite questionnaire predicts weight loss in community-dwelling adults and nursing home residents. Am J Clin Nutr 2005;82(5):1074-81. doi: 10.1093/ajcn/82.5.1074
59. Besson H, Brage S, Jakes RW, et al. Estimating physical activity energy expenditure, sedentary time, and physical activity intensity by self-report in adults. Am J Clin Nutr 2010;91(1):106-14. doi: 10.3945/ajcn.2009.28432 [published Online First: 20091104]
60. Jutten RJ, Harrison JE, Brunner AJ, et al. The Cognitive-Functional Composite is sensitive to clinical progression in early dementia: Longitudinal findings from the Catch-Cog study cohort. Alzheimers Dement (N Y) 2020;6(1):e12020. doi: 10.1002/trc2.12020 [published Online First: 20200417]
61. Lezak MD, Howieson DB, Loring DW. Neuropsychological Assessment. 4th ed: New York: Oxford University Press 2004.

For peer review only - http://bmjopen.bmj.com/site/about/guidelines.xhtml

62. Zhao Q, Lv Y, Zhou Y, et al. Short-term delayed recall of auditory verbal learning test is equivalent to long-term delayed recall for identifying amnestic mild cognitive impairment. PLoS One 2012;7(12):e51157. doi: 10.1371/journal.pone.0051157 [published Online First: 20121207]
63. Guralnik JM, Simonsick EM, Ferrucci L, et al. A short physical performance battery assessing lower extremity function: association with self-reported disability and prediction of mortality and nursing home admission. J Gerontol 1994;49(2):M85-94. doi: 10.1093/geronj/49.2.m85
64. Ware JE, Jr., Sherbourne CD. The MOS 36-item short-form health survey (SF-36). I. Conceptual framework and item selection. Med Care 1992;30(6):473-83.
65. Yesavage JA, Brink TL, Rose TL, et al. Development and validation of a geriatric depression screening scale: a preliminary report. J Psychiatr Res 1982;17(1):37-49. doi: 10.1016/0022-3956(82)90033-4
66. Vellas B, Villars H, Abellan G, et al. Overview of the MNA--Its history and challenges. J Nutr Health Aging 2006;10(6):456-63; discussion 63-5.
67. Schröder H, Fitó M, Estruch R, et al. A short screener is valid for assessing Mediterranean diet adherence among older Spanish men and women. J Nutr 2011;141(6):1140-5. doi: 10.3945/jn.110.135566 [published Online First: 20110420]
68. Genton L, Karsegard VL, Kyle UG, et al. Comparison of Four Bioelectrical Impedance Analysis Formulas in Healthy Elderly Subjects. Gerontology 2001;47:315 - 23.
69. Moher D, Hopewell S, Schulz KF, et al. CONSORT 2010 Explanation and Elaboration: Updated guidelines for reporting parallel group randomised trials. J Clin Epidemiol 2010;63(8):e1-37. doi: 10.1016/j.jclinepi.2010.03.004 [published Online First: 20100325]
70. Franco-Marina F, García-González JJ, Wagner-Echeagaray F, et al. The Mini-mental State Examination revisited: ceiling and floor effects after score adjustment for educational level in an aging Mexican population. Int Psychogeriatr 2010;22(1):72-81. doi: 10.1017/s1041610209990822 [published Online First: 20090907]

Figure 1: Overview of PROMED-EX Study Design

For peer review only

For peer review only - http://bmjopen.bmj.com/site/about/guidelines.xhtml

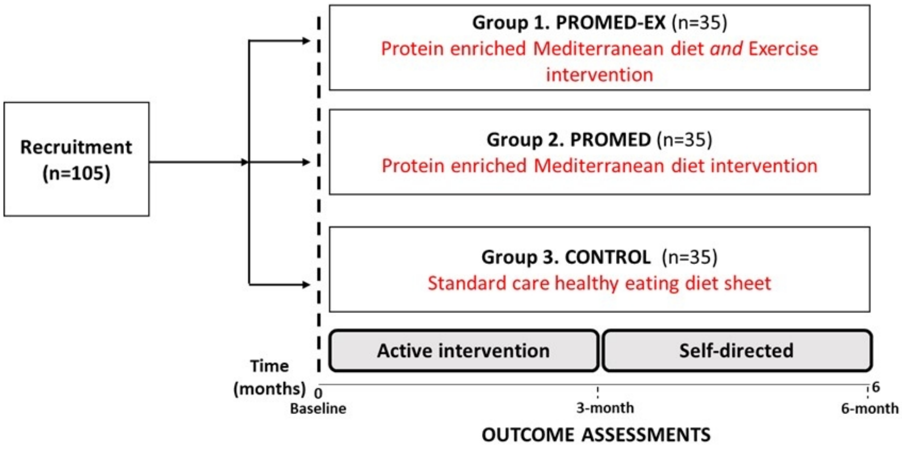

Overview of PROMED-EX Study Design

159x72mm (150 x 150 DPI)

STANDARD PROTOCOL ITEMS: RECOMMENDATIONS FOR INTERVENTIONAL TRIALS

SPIRIT 2013 Checklist: Recommended items to address in a clinical trial protocol and related documents*

Section/item	Item No	Description	Addressed on page number
**Administrative information**			
Title	1	Descriptive title identifying the study design, population, interventions, and, if applicable, trial acronym	**1**
Trial registration	2a	Trial identifier and registry name. If not yet registered, name of intended registry	**1**
	2b	All items from the World Health Organization Trial Registration Data Set	**1**
Protocol version	3	Date and version identifier	**14**
Funding	4	Sources and types of financial, material, and other support	**14**
Roles and responsibilities	5a	Names, affiliations, and roles of protocol contributors	**1,14**
	5b	Name and contact information for the trial sponsor	**1**
	5c	Role of study sponsor and funders, if any, in study design; collection, management, analysis, and interpretation of data; writing of the report; and the decision to submit the report for publication, including whether they will have ultimate authority over any of these activities	**14**
	5d	Composition, roles, and responsibilities of the coordinating centre, steering committee, endpoint adjudication committee, data management team, and other individuals or groups overseeing the trial, if applicable (see Item 21a for data monitoring committee)	**Appendix 2**

For peer review only
For peer review only - http://bmjopen.bmj.com/site/about/guidelines.xhtml

Introduction

Background and rationale	6a	Description of research question and justification for undertaking the trial, including summary of relevant studies (published and unpublished) examining benefits and harms for each intervention	**3,4**
	6b	Explanation for choice of comparators	**4**
Objectives	7	Specific objectives or hypotheses	**4**
Trial design	8	Description of trial design including type of trial (eg, parallel group, crossover, factorial, single group), allocation ratio, and framework (eg, superiority, equivalence, noninferiority, exploratory)	**4**

Methods: Participants, interventions, and outcomes

Study setting	9	Description of study settings (eg, community clinic, academic hospital) and list of countries where data will be collected. Reference to where list of study sites can be obtained	**5**
Eligibility criteria	10	Inclusion and exclusion criteria for participants. If applicable, eligibility criteria for study centres and individuals who will perform the interventions (eg, surgeons, psychotherapists)	**5,6**
Interventions	11a	Interventions for each group with sufficient detail to allow replication, including how and when they will be administered	**6-8**
	11b	Criteria for discontinuing or modifying allocated interventions for a given trial participant (eg, drug dose change in response to harms, participant request, or improving/worsening disease)	**N/A**
	11c	Strategies to improve adherence to intervention protocols, and any procedures for monitoring adherence (eg, drug tablet return, laboratory tests)	**8**
	11d	Relevant concomitant care and interventions that are permitted or prohibited during the trial	**6-8**
Outcomes	12	Primary, secondary, and other outcomes, including the specific measurement variable (eg, systolic blood pressure), analysis metric (eg, change from baseline, final value, time to event), method of aggregation (eg, median, proportion), and time point for each outcome. Explanation of the clinical relevance of chosen efficacy and harm outcomes is strongly recommended	**10,11**
Participant timeline	13	Time schedule of enrolment, interventions (including any run-ins and washouts), assessments, and visits for participants. A schematic diagram is highly recommended (see Figure)	**5 (Figure 1)**

For peer review only - http://bmjopen.bmj.com/site/about/guidelines.xhtml

Sample size	14	Estimated number of participants needed to achieve study objectives and how it was determined, including clinical and statistical assumptions supporting any sample size calculations	**11**
Recruitment	15	Strategies for achieving adequate participant enrolment to reach target sample size	**5**
**Methods: Assignment of interventions (for controlled trials)**			
Allocation:			
Sequence generation	16a	Method of generating the allocation sequence (eg, computer-generated random numbers), and list of any factors for stratification. To reduce predictability of a random sequence, details of any planned restriction (eg, blocking) should be provided in a separate document that is unavailable to those who enrol participants or assign interventions	**6**
Allocation concealment mechanism	16b	Mechanism of implementing the allocation sequence (eg, central telephone; sequentially numbered, opaque, sealed envelopes), describing any steps to conceal the sequence until interventions are assigned	**6**
Implementation	16c	Who will generate the allocation sequence, who will enrol participants, and who will assign participants to interventions	**6**
Blinding (masking)	17a	Who will be blinded after assignment to interventions (eg, trial participants, care providers, outcome assessors, data analysts), and how	**6**
	17b	If blinded, circumstances under which unblinding is permissible, and procedure for revealing a participant's allocated intervention during the trial	**N/A**
**Methods: Data collection, management, and analysis**			
Data collection methods	18a	Plans for assessment and collection of outcome, baseline, and other trial data, including any related processes to promote data quality (eg, duplicate measurements, training of assessors) and a description of study instruments (eg, questionnaires, laboratory tests) along with their reliability and validity, if known. Reference to where data collection forms can be found, if not in the protocol	**8-11**
	18b	Plans to promote participant retention and complete follow-up, including list of any outcome data to be collected for participants who discontinue or deviate from intervention protocols	**11**

Data management	19	Plans for data entry, coding, security, and storage, including any related processes to promote data quality (eg, double data entry; range checks for data values). Reference to where details of data management procedures can be found, if not in the protocol	**12**
Statistical methods	20a	Statistical methods for analysing primary and secondary outcomes. Reference to where other details of the statistical analysis plan can be found, if not in the protocol	**11**
	20b	Methods for any additional analyses (eg, subgroup and adjusted analyses)	**N/A**
	20c	Definition of analysis population relating to protocol non-adherence (eg, as randomised analysis), and any statistical methods to handle missing data (eg, multiple imputation)	**12**
**Methods: Monitoring**			
Data monitoring	21a	Composition of data monitoring committee (DMC); summary of its role and reporting structure; statement of whether it is independent from the sponsor and competing interests; and reference to where further details about its charter can be found, if not in the protocol. Alternatively, an explanation of why a DMC is not needed	**N/A -Appendix 1**
	21b	Description of any interim analyses and stopping guidelines, including who will have access to these interim results and make the final decision to terminate the trial	**N/A (no interim analysis)**
Harms	22	Plans for collecting, assessing, reporting, and managing solicited and spontaneously reported adverse events and other unintended effects of trial interventions or trial conduct	**11**
Auditing	23	Frequency and procedures for auditing trial conduct, if any, and whether the process will be independent from investigators and the sponsor	**None**
**Ethics and dissemination**			
Research ethics approval	24	Plans for seeking research ethics committee/institutional review board (REC/IRB) approval	**12**
Protocol amendments	25	Plans for communicating important protocol modifications (eg, changes to eligibility criteria, outcomes, analyses) to relevant parties (eg, investigators, REC/IRBs, trial participants, trial registries, journals, regulators)	**14**

For peer review only - http://bmjopen.bmj.com/site/about/guidelines.xhtml

Consent or assent	26a	Who will obtain informed consent or assent from potential trial participants or authorised surrogates, and how (see Item 32)	**6, Appendix 4**
	26b	Additional consent provisions for collection and use of participant data and biological specimens in ancillary studies, if applicable	**Appendix 4**
Confidentiality	27	How personal information about potential and enrolled participants will be collected, shared, and maintained in order to protect confidentiality before, during, and after the trial	**12**
Declaration of interests	28	Financial and other competing interests for principal investigators for the overall trial and each study site	**14**
Access to data	29	Statement of who will have access to the final trial dataset, and disclosure of contractual agreements that limit such access for investigators	**12**
Ancillary and post-trial care	30	Provisions, if any, for ancillary and post-trial care, and for compensation to those who suffer harm from trial participation	**N/A**
Dissemination policy	31a	Plans for investigators and sponsor to communicate trial results to participants, healthcare professionals, the public, and other relevant groups (eg, via publication, reporting in results databases, or other data sharing arrangements), including any publication restrictions	**12**
	31b	Authorship eligibility guidelines and any intended use of professional writers	**14**
	31c	Plans, if any, for granting public access to the full protocol, participant-level dataset, and statistical code	**12**
**Appendices**			
Informed consent materials	32	Model consent form and other related documentation given to participants and authorised surrogates	**Appendix 4**
Biological specimens	33	Plans for collection, laboratory evaluation, and storage of biological specimens for genetic or molecular analysis in the current trial and for future use in ancillary studies, if applicable	**Not provided**

*It is strongly recommended that this checklist be read in conjunction with the SPIRIT 2013 Explanation & Elaboration for important clarification on the items. Amendments to the protocol should be tracked and dated. The SPIRIT checklist is copyrighted by the SPIRIT Group under the Creative Commons "Attribution-NonCommercial-NoDerivs 3.0 Unported" license.

For peer review only

Appendix 2- PROMED-EX Trial Steering Committee (TSC)

Table S1: TSC members and their roles

Member	Role
**Prof Emma Stevenson**	Independent Chair
**Prof Jayne Woodside**	Dependant- scientific advisor
**Prof Dorothee Volkert**	Dependant- scientific advisor
**Prof Lisette de Groot**	Dependant- scientific advisor
**Prof Lorraine Brennan**	Dependant- scientific advisor
**Prof Chris Cardwell**	Dependant- statistician
**Ms Bernie Devlin**	Independent lay member
**Ms Eleanor Butterwick**	Independent lay member
**Ms Clare Jess**	Facilitator
**Dr Roisin O'Neill/ Dr Dominic Farsi**	Dependant- Trial manager
**Dr Claire McEvoy**	Dependant- PROMED-EX principal investigator

Table S2: Roles and responsibilities of the TSC

**Roles and responsibilities**	
Aims of the TSC	To act as the oversight body for PROMED-EX on behalf of the Sponsor/Funder.
Terms of reference	The role of the TSC is to provide oversight for the trial [Medical Research Council (MRC) Good Clinical Practice (GCP) guidelines (1998)]. It should also provide advice through its independent Chairperson to the PI, local trial team, BBSRC on all aspects of the trial.
Specific roles of TSC	• provide expert oversight of the trial<br>• maintain confidentiality of all trial information that is not already in the public domain<br>• make decisions as to the future continuation (or otherwise) of the trial/s<br>• monitor recruitment rates and encourage the trial team to develop strategies to deal with any recruitment problems<br>• review regular reports of the trial from the trial team.<br>• assess the impact and relevance of any accumulating external evidence<br>• monitor completion of CRFs and comment on strategies from trial team to encourage satisfactory completion in the future<br>• monitor follow-up rates and review strategies from Trial team to deal with problems<br>• approve any amendments to the protocol, where appropriate<br>• approve any proposals by the Trial team concerning any change to the design of the trial, including additional substudies<br>• oversee the timely reporting of trial results<br>• comment on the main trial manuscript<br>• comment on any abstracts and presentations of any results *during* the running of the trial

Study no ____________ Date____________

PROMED DIET SCORE

Visit: Baseline ☐ 3 mon ☐ 6 mon ☐

Questions	Self-reported	Criteria for 1 point	Point?
1. Do you use olive oil or rapeseed oil as your main cooking fat?		Yes	
2. How much olive/ rapeseed oil do you consume in a given day? Including oil used for frying, salads, out-of-house meals etc (in tablespoons)<br><br>How much olive or rapeseed oil-based spread do you consume in a given day? (in teaspoons)		≥1 tbsp oil / day<br><br>**and/or**<br><br>3 tsp spread/ day	
3. How many servings of dairy foods (milk, cheese, yogurts) or plant proteins alternatives do you eat per day?		≥2 portions per day	
4. How many portions of fruit (including natural fruit juices) do you consume per day? (1 portion = 1 apple/banana (80g), small glass juice (150ml)		≥2 portions/ day	
5. How many vegetable servings do you consume per day? Including raw/ cooked vegetables, salad but not including potatoes (1 serving: 3 tablespoons/80g)		≥3 portions/ day	
6. How many servings of legumes (peas, beans and lentils including kidney beans, baked beans, chickpeas, red lentils etc) do you consume per week? (1 serving :3 tablespoons/ 80g)		≥3 servings/ week	
7. How many servings of red meat including beef, pork, lamb and minced beef do you consume per week? (1 serving: medium portion/ 100–150 g)		≤2 servings /week	
8. How many servings of processed meat including ham, bacon, sausages, meat pies and other meat products etc.) do you consume per week? (1 serving: medium portion/ 100–150 g)		≤1 serving /week	
9. How many servings of chicken/ turkey do you consume per week? (1 serving: medium portion/ 100-150g)		≥2 servings /week	
10. How many servings of fish (tuna, cod, haddock, salmon, mackerel, herring, and sardines etc, including tinned varieties, excluding crumbed or battered fish) or shellfish do you consume per week? (1 serving: 1 fillet/small fish or 140g)		≥2 servings /week	
11. Do you preferentially consume wholegrain bread and/ or cereal and/ or rice and/ or pasta instead of non-wholegrain (white) varieties?		Yes	
12. How many servings of natural nuts do you consume per week? (1 serving: 1 small handful/ 30 g)		≥3 servings /week	
13. How many times per week do you consume sweet foods (including biscuits, buns, pastries, chocolate, sweets, sweet or carbonated beverages or desserts)?		≤3 times/ week	
14. How often would you consume up to 3 small glasses of wine or equivalent other alcoholic beverages per week? (1 small glass:125ml)		1-3 glasses or equivalent ≥ 3 days/ week	

For peer review only

BMJ Open

Study Protocol: Randomised controlled trial to evaluate the effects of a PROtein enriched MEDiterranean Diet and EXercise on nutritional status and cognition in undernourished adults with subjective cognitive decline: The PROMED-EX Study.

Journal:	*BMJ Open*
Manuscript ID	bmjopen-2022-070689.R2
Article Type:	Protocol
Date Submitted by the Author:	08-Aug-2023

Complete List of Authors:	Ward, Nicola Ann; Queen's University Belfast, Centre for Public Health<br>Reid-McCann, Rachel; Queen's University Belfast, Centre for Public Health<br>Brennan, Lorraine; University College Dublin School of Agriculture and Food Science, Institute of Food and Health and Conway Institute<br>Cardwell, Christopher; Queen's University Belfast, Centre for Public Health<br>de Groot , LC; Wageningen University, Division of Human Nutrition<br>Maggi, Stefania; Neuroscience Research Institute National Research Council Padua Branch, Aging Branch<br>McCaffrey, Noel; c/o Irish Wheelchair Association, ExWell Medical<br>McGuinness, Bernadette; Queen's University Belfast, Centre for Public Health<br>McKinley, Michelle C.; Queen's University Belfast, Centre for Public Health<br>Noale, M; Neuroscience Research Institute National Research Council Padua Branch, Aging Branch<br>O'Neill, Roisin; Queen's University Belfast, Centre for Public Health<br>Prinelli, Federica; Institute for Biomedical Technologies National Research Council, Epidemiology Unit<br>Sergi, Giuseppe; University of Ferrara, Department of Medical Sciences<br>Trevisan, Caterina.; University of Ferrara Department of Medical Sciences, Department of Medical Sciences<br>Volkert, Dorothee; Friedrich-Alexander-Universitat Erlangen-Nurnberg, Institute for Biomedicine of Aging<br>Woodside, Jayne; Queen's University Belfast, Centre for Public Health<br>McEvoy, Claire T.; Queen's University Belfast, Centre for Public Health; Global Brain Health Institute, Trinity College Dublin, Ireland & University of California San Francisco, USA

**Primary Subject Heading**:	Public health
Secondary Subject Heading:	Neurology, Public health, Sports and exercise medicine, Nutrition and metabolism
Keywords:	Nutrition < TROPICAL MEDICINE, Dementia < NEUROLOGY, NUTRITION

	& DIETETICS, PUBLIC HEALTH, PREVENTIVE MEDICINE

SCHOLARONE™
Manuscripts

Study Protocol: Randomised controlled trial to evaluate the effects of a PROtein enriched MEDiterranean Diet and EXercise on nutritional status and cognition in undernourished adults with subjective cognitive decline: The PROMED-EX Study.

Ward N[1], Reid-McCann R[1], Brennan L[2], Cardwell C[1], de Groot CPGM[3], Maggi S[4], McCaffrey N[5], McGuinness B[1], McKinley MC[1], Noale M[4], O'Neill RF[1], Prinelli F[6], Sergi G[7], Trevisan C[7], Volkert D[8], Woodside JV[1], McEvoy CT[1,9] and the PROMED-COG consortium.

[1]Centre for Public Health, Queen's University Belfast, Belfast. Northern Ireland. UK.

[2]School of Agriculture and Food Science, Institute of Food and Health and Conway Institute, University College Dublin, Dublin. Ireland.

[3]Division of Human Nutrition, Wageningen University, Wageningen. Netherlands

[4] Neuroscience Institute, Aging Branch, National Research Council (CNR), Padua. Italy.

[5]ExWell Medical, c/o Irish Wheelchair Association, Dublin. Ireland.

[6] Institute of Biomedical Technologies, Epidemiology Unit, National Research Council (CNR), Segrate (MI). Italy

[7]Department of Medical Sciences, University of Ferrara, Ferrara. Italy

[8]Institute for Biomedicine of Aging, Friedrich-Alexander-Universität Erlangen-Nürnberg, Nuremberg. Germany.

[9]The Global Brain Institute, Trinity College Dublin, Ireland & University of California San Francisco, USA.

Corresponding Author: Dr Claire McEvoy, Centre for Public Health, Institute of Clinical Sciences building A, Queen's University Belfast, Grosvenor Road, Belfast. BT12 6BJ. Northern Ireland. UK. Email: **c.mcevoy@qub.ac.uk**

Word count, excluding title page, abstract, references, figures, and table

Key Words: nutrition, exercise, cognitive function, dementia

For peer review only - http://bmjopen.bmj.com/site/about/guidelines.xhtml

ABSTRACT

Introduction Undernutrition leading to unplanned weight loss is common in older age and has been linked to increased dementia risk in later life. Weight loss can precede dementia by a decade or more, providing a unique opportunity for early intervention to correct undernutrition and potentially prevent or delay cognitive impairment. The combined effects of diet and exercise on undernutrition have not yet been evaluated. The objective of the PROMED-EX trial is to determine the effect of a protein-enriched Mediterranean Diet, with and without exercise, on nutritional status and cognitive performance in older adults at risk of undernutrition and cognitive decline.

Methods One hundred and five participants aged 60 years and over at risk of undernutrition and with subjective cognitive decline will be recruited to participate in a 6-month, single-blind, parallel-group randomised controlled trial. Participants will be block randomised into one of three groups: Group 1. PROMED-EX (diet + exercise), Group 2. PROMED (diet only), and Group 3. Standard Care (control). The primary outcome is nutritional status measured using the Mini Nutritional Assessment (MNA). Secondary outcomes include cognitive function, nutritional intake, body composition, physical function, and quality of life. Mechanistic pathways for potential diet and exercise-induced change in nutritional status and cognition will be explored by measuring inflammatory, metabolic, nutritional and metabolomic biomarkers.

Ethics and dissemination The study is approved by the UK Office for Research Ethics Committee (Ref 21/NW/0215). Written informed consent will be obtained from participants prior to recruitment. Research results will be disseminated to the public via meetings and media and the scientific community through conference presentations and publication in academic journals.

Trial registration NCT 05166564.

Strengths and limitations of this study

- PROMED-EX is the first randomised controlled trial designed to evaluate the effect of an optimised protein-enriched Mediterranean diet, with and without exercise, on nutritional status and cognitive performance in a high-risk population.
- The integration of state-of-the-art metabolomics, metabolic, inflammatory, and nutritional biomarkers will help elucidate biological mechanisms underlying how diet can influence nutrition status and cognition and how exercise can interact with nutrition to modify these processes.
- A Personal and Public Involvement (PPI) group will contribute at all stages of the PROMED-EX trial to optimise the research design and dissemination strategy of the research findings.
- The nature of the 'real world' pragmatic study design means that it is not possible to blind participants to treatment allocation, possibly leading to performance bias.
- Data will be analysed by a blinded member of the study team to minimise detection bias.

For peer review only - http://bmjopen.bmj.com/site/about/guidelines.xhtml

INTRODUCTION

Undernutrition, defined as insufficient food and/or nutrient intake that results in altered body composition and unplanned weight loss,[1] is common in older age and affects >1.3 million older adults in the UK.[2] Individuals with cognitive impairment are particularly vulnerable to undernutrition and its many adverse consequences. Undernourished people with dementia experience more rapid decline in cognitive and physical functions, increased neuropsychiatric symptoms, and greater risk of hospitalisation and mortality, than those with adequate nutritional status.[3,4] Being undernourished has also emerged as a potentially modifiable risk factor for dementia.[5] In prospective studies, weight loss has been associated with increased dementia risk,[6] and precedes the onset of dementia by a decade or more,[7,8] making it an intervention target that should be prioritised.

Causes of undernutrition are increased nutrient requirements, inadequate dietary intake and reduced nutrient bioavailability,[9] but the exact mechanisms of undernutrition in dementia are not clear. It is possible that weight loss may be both a consequence and cause of neurodegeneration and dementia. Loss of muscle and weight in older adults has been linked to accelerated decline in brain structures,[10-12] even after exclusion of cognitive impairment,[12] suggesting a pathophysiological role of undernutrition in dementia. In contrast, weight loss could be an early marker of dementia development. Pathological changes in the brain are present 10-15 years prior to the clinical symptoms of cognitive impairment and induce inflammatory[13] and physiological changes beyond normal ageing that can have a negative impact on nutrition and body weight. Dementia pathology affects brain regions such as the hypothalamus which is important for appetite regulation and eating behaviour.[14] This may explain alterations of leptin and ghrelin metabolic signals that are observed in early disease.[15] Early brain changes can also affect chemosensory perception including taste and olfactory function,[16] as well as satiety regulation, protein and lipid synthesis,[17] and food preferences. Disease progression and worsening cognitive impairment can cause forgetfulness to eat, dysphagia,[18] sleep disturbance[19] and social withdrawal,[20] which can all have adverse effects on food intake.[21]

To date, most studies have focused on the impact of diet on physical function,[22] with limited number of interventions targeting undernutrition for cognitive benefit. Short-term treatment of undernutrition has been shown to improve both nutritional status and cognitive performance.[23-25] However, small sample sizes as well as differences in residential status of study populations and nutritional interventions (dietetic counselling,[23] oral supplementation,[24,25] meal provision[25]), and cognitive outcomes make it difficult to know whether addressing undernutrition could protect against cognitive decline. Hence, trials are warranted to test the efficacy of intervention for undernutrition in populations at increased risk of cognitive decline but not yet cognitively impaired and may benefit from early intervention.

The Mediterranean Diet (MedDiet) is a nutrient dense dietary pattern with proven anti-inflammatory benefits[26] and may offer protection against cognitive decline during ageing. Observational data have reported links between the MedDiet and favourable changes in brain structures and functions,[27] slower rate of cognitive decline[28,29] and decreased dementia risk, [30,31] which appear to be attributable to the combined effect of different foods rather than the action of a single food or nutrient.[32] Furthermore, the MedDiet is associated with nutritional adequacy and lower risk of undernutrition and micronutrient deficiency.[33] Adequate protein (1.0-1.2/kg body weight/day)[34] and energy intake[35] are critical for preventing muscle loss[36] and undernutrition in older adults.[37] However, emerging data

For peer review only - http://bmjopen.bmj.com/site/about/guidelines.xhtml

suggest that a high-quality dietary pattern is also important,[38] lending support to a 'whole diet' approach for preventing muscle loss and functional decline during ageing.

Physical exercise has positive effects on cognition,[39] especially in domains of executive function and processing speed,[40] by increasing neurogenesis and upregulating neurotrophic factors.[41] However, it is not known how diet and exercise interact for neuroprotection. Multi-domain interventions that include both a healthy diet and exercise appear to elicit stronger positive results for cognitive performance than each intervention in isolation.[42] Furthermore, exercise can stimulate protein synthesis[43] and enhance muscle mass and functional performance in older adults,[44] making it an attractive target for preventing undernutrition. An optimal protein MedDiet may induce additive effects on nutritional status and cognition when combined with exercise, but this has not yet been evaluated.

The PROtein enriched MEDiterranean Diet and EXercise (PROMED-EX) randomised controlled trial (RCT) aims to determine the effects of a protein-enriched MedDiet, with and without exercise, in comparison to standard care, on nutritional status in adults at risk of undernutrition and cognitive decline. PROMED-EX is part of the European "PROMED-COG" project, funded under the Horizon Joint Programming Initiative 'a healthy diet for a healthy life' to establish evidence for the balance between diet and physical activity for preventing undernutrition and promoting healthy neurocognitive ageing.[45]

METHODS AND ANALYSIS

PROMED-EX is a 6-month, single-blind, parallel-group RCT aiming to recruit 105 eligible adults residing in Northern Ireland (NI), aged 60 years+ and at risk of both undernutrition and cognitive decline. The protocol has been written in accordance with the Standard Protocol Items: Recommendations for Interventional Trials (SPIRIT) guidelines,[46] and a summary is reported using the checklist (Appendix 1). An independent Trial Steering Committee (TSC) has been established (Appendix 2) to oversee the conduct of the study.

Patient and public involvement (PPI)

A patient and public involvement (PPI) group (n=8) comprising older adults residing in NI with lived experience of subjective cognitive decline (SCD) and/or poor nutritional status were recruited for PROMED-EX. PPI members have contributed to the intervention design and co-development of materials, including lay summary, participant information sheet and consent form, advertisement materials, written educational resources for the interventions. Besides the trial design, two PPI members sit on the TSC and will input to future stages including implementation, analysis, and dissemination.

Study Design

Study participants will be randomised into one of three groups: Group 1. PROMED-EX: Protein-enriched MedDiet and exercise intervention (n=35); Group 2. PROMED: Protein-enriched MedDiet intervention (n=35); or Group 3. Control: Standard care healthy eating diet sheet (n=35) as shown in Figure 1. Participants will be informed of their allocated group after baseline data has been collected. Those randomised to the PROMED-EX and PROMED interventions will receive active support to adopt the behavioural targets in the first 3 months, after which time they will continue with self-directed maintenance of the newly adopted behaviours for a further 3 months.

Sample Size

For peer review only - http://bmjopen.bmj.com/site/about/guidelines.xhtml

As no prior studies have evaluated the effects of whole diet and exercise on nutritional status and cognition, the sample size calculation is based on a standard deviation of the change in MNA score of 4 points over a similar period from the control group of a previous nutrition intervention.[47] A total of 28 completing in each intervention and control group would provide over 80% power to detect a difference in the three means, based upon a statistically significant ANOVA result at the 5% level, hypothesising a true increase in MNA of 3 or more in both intervention groups, but not the control group. Loss to follow-up is estimated from our prior experience of conducting lifestyle trials in older adults[48-50] where attrition ranges from 6%[50] to 30%.[49] Considering, FINGER[42] evaluated an intensive multidomain lifestyle intervention among older adults at increased dementia risk and reported 12% withdrawal over 2 years. We anticipate dropout at 20% and therefore have added 20% to the sample size to account for non-compliance, dropout, or discontinuation.

Recruitment and Eligibility of Study Participants

Multiple recruitment strategies will be employed including paid Facebook advertisements, other media (e.g., newspaper, radio, and television), and identification of potentially eligible participants from community networks, primary care sectors and health and social care trusts in NI. Those interested in participating will be provided with an information sheet detailing the study protocol, followed by a phone call to discuss study procedures.

Potentially eligible participants will be screened by telephone/video to last around 45 minutes. Individuals will be eligible to enrol if they are community-dwelling, aged ≥60 years, at risk of undernutrition defined by a Mini Nutritional Assessment -Short Form (MNA-SF) score of 8-11[51] and have a positive screen for SCD.[52] The screening assessment includes the Telephone cognitive screen (T-CogS) or Mini Mental State Examination (MMSE) to exclude participants with cognitive impairment and a health and medical history questionnaire to determine if the participant has any medical conditions that compromise their ability to take part. This includes if they receive nutritional support, have a diagnosis of mild cognitive impairment or dementia, or dietary restrictions as shown in Table 1.

Randomisation, blinding and allocation

A study statistician independent of recruitment, intervention delivery and data collection will perform block randomisation using a computer-generated random number sequence in blocks of size 6 (using command RALLOC in STATA Release 16, StataCorp LP, College Station). Allocations will be prepared in sealed opaque envelopes by an independent study administrator and will not be revealed to participants until completion of baseline data collection. It will not be possible to blind the participant nor study researchers involved in intervention delivery to the allocated intervention group; however, data collection and analysis will be carried out by investigators blinded to treatment allocation.

Table 1: PROMED-EX Trial eligibility criteria

Inclusion Criteria	Exclusion Criteria

For peer review only - http://bmjopen.bmj.com/site/about/guidelines.xhtml

• Age ≥ 60 years<br>• Community-dwelling<br>• Mini Nutrition Assessment-Short Form (MNA-SF) score 8-11, indicating increased risk of undernutrition[51]<br><br>• Positive screen for subjective cognitive decline (SCD)[52] defined as a 'yes' response to the question: *"During the past 12 months, have you experienced confusion or slight memory loss that is happening more often or is getting worse?"*<br><br>• Ability to provide informed consent.	• Cognitive impairment: Telephone cognitive screen (T-CogS) ≤*21* or Mini Mental State Examination (MMSE) ≤*24* or clinical diagnosis of mild cognitive impairment or dementia<br>• In receipt of prescribed oral or artificial nutritional support<br>• Dietary restrictions/allergies to PROMED foods (e.g. nuts) or taking high-dose nutritional supplements (e.g. Vitamin E) that limit the ability to take part in the study<br>• Living in long-term care facilities<br>• Self-reported:<br>○ Dysphagia<br>○ Chronic Kidney disease<br>○ Diabetes complications or HbA1c >8%<br>○ Significant medical comorbidities that may alter cognitive test performance, e.g., stroke, brain injury<br>• Inability to complete cognitive testing due to language or visual impairment

Interventions

Group 1. PROMED-EX

Protein enriched MedDiet intervention

The PROMED-EX Trial will be overseen by a registered dietitian (CME). Participants will meet a trained researcher, with a background in nutrition and exercise within one week following randomisation to receive personalised dietary counselling based on their baseline anthropometry, MNA score and PROMED diet score (Appendix 3). This session will last up to 60 minutes where education will be provided on adequate nutritional status, avoiding weight loss, and encouraging regular meal pattern. Participants will be encouraged to set dietary goals to adopt PROMED diet behaviours (shown in Table 2) to achieve overall intervention targets: (1) increased protein intake up to 1.5g per kg current body weight;[37] (2) increased MNA score by ≥2 points; and (3) increased PROMED diet score by ≥2 points. Daily energy requirements will be calculated at 30kcal/kg of body weight with a supplement of 400-600kcal only if weight gain is indicated.[53]

For peer review only - http://bmjopen.bmj.com/site/about/guidelines.xhtml

Table 2: PROMED diet behaviours

**Daily**
1tbsp of olive oil<br>≥ 2 portions fruit<br>≥3 portions vegetables<br>≥2 portions of wholegrain foods (including wholemeal bread, wholegrain rice, whole wheat pasta)<br>≥2 portions of dairy foods (including, milk, cheese, yoghurts) or plant protein alternatives<br>Moderate alcohol (1-2 glasses of wine/beer consumed with meals) for alcohol consumers
**Weekly**
Preferential consumption of chicken and oily fish over red meat, ≥2 portions of each and red meat <2 portions<br>3 handfuls of natural nuts (90g)

Written materials (PROMED diet guide and recipe book) will be provided to support adoption of diet behaviours. Participants will be given a recipe book with suggested quick, low-cost menu plans and practical dietary tips to overcome small appetite. Key foods will be delivered to their home free of charge for the first 3-months to encourage adoption of the PROMED diet behaviours. Foods will be self-selected by participants from a pre-defined list to facilitate personal preference and will include olive oil, fruits and vegetables, wholegrain foods (cereals, breads, rice and pasta), nuts, nut butters, canned oily fish and low-fat dairy foods.

Exercise intervention

The exercise intervention has been adapted from a commercially available 'ExWell@home' group exercise rehabilitation programme that is acceptable to older adults and suggested to improve health outcomes in those with chronic disease.[54] The home-based exercise intervention comprises strength, aerobic, core stability and balance exercises available across four levels of ability: (1) chair based, (2) basic, (3) standard and (4) advanced. After the dietary education session, participants will receive a personal exercise prescription involving a written plan, demonstration of exercises suitable for their fitness level, and instruction on safety aspects to minimise injury risk. A researcher trained to deliver the adapted Exwell programme, will determine baseline fitness level using measured blood pressure, pulse rate, short physical performance test results, handgrip strength test obtained from the baseline study visit, and their assessed performance in a ‘basic level’ class.

Participants will be asked to report their perceived intensity of exertion using the Borg Rate of Perceived Exertion (RPE) scale (6-20),[55] aiming for "somewhat hard" within a range of 12-14 points. Participants will be instructed to increase or decrease the frequency and intensity of exercise over the first 4 weeks to meet the intervention target of two x 30–60-

For peer review only - http://bmjopen.bmj.com/site/about/guidelines.xhtml

minute moderate intensity exercise sessions per week. Participants will receive additional supportive materials to achieve their exercise goals including pre-recorded exercise classes, a written home-based exercise resource, a pedometer, and exercise diary.

Group 2. PROMED

Participants will receive only the Protein enriched MedDiet intervention and will be advised to keep other lifestyle factors unchanged throughout the study period, including maintenance of their usual physical activity level.

Group 3. Standard care

Participants will receive a general healthy eating diet sheet provided at baseline. To encourage retention in the study, personalised dietary advice and written materials will be offered to control group participants upon completion of the final study assessment.

Maximising and monitoring compliance with the diet and exercise interventions

For study participants randomised to the PROMED-EX and PROMED interventions (Groups 1 and 2 respectively) several approaches will be used to facilitate behaviour change and encourage compliance with the diet and exercise goals. The initial education session will be scheduled at a convenient time for the participant and in a convenient location (either in a dedicated research facility, the participants own home or via remote video call). Partners or caregivers who are responsible for food preparation and/or shopping will also be invited to the education session if applicable. Furthermore, techniques demonstrated to support diet and exercise behaviour change have been incorporated into the intervention written materials and include ‘instruction on how to perform behaviour’, ‘action planning’, ‘self-monitoring’ and ‘problem solving.’[56]

Participants will be encouraged to self-monitor their compliance in achieving the diet and exercise goals where applicable. The researcher will contact participants weekly via telephone during the first 3-months to review individual progress, monitor compliance and body weight, deal with any difficulties participants may be experiencing in meeting their goals and provide support and personalised feedback as required. Participants will also be able to contact the researcher at any time during the 6-month intervention period.

Data collection and Study outcomes

Participants will meet with a researcher at baseline and after 3- and 6-months to complete a study assessment lasting no longer than 2.5-hours. The assessment will take place in a dedicated research facility at Queen’s University Belfast (QUB) or in the participant’s home if preferred. A summary of the outcomes and data collection measures at study time-points are found in Table 3.

Table 3: Overview of study data collection variables and measures

Study outcomes	Data collection variables/ measures	Baseline	3-month	6-month

For peer review only - http://bmjopen.bmj.com/site/about/guidelines.xhtml

**Primary outcome**				
Nutritional status (6-months)	Mini Nutrition Assessment (MNA®) long-form questionnaire[57]	☐		☐
**Secondary outcomes**				
Nutrition and body composition				
Nutritional status (midpoint change)	MNA® long-form	☐	☐	
Nutritional intake	PROMED diet score questionnaire and 4-day food diary	☐	☐	☐
Appetite	Simplified Nutrition Appetite Questionnaire (SNAQ)[58]	☐	☐	☐
Body composition	Bioelectric Impedance Analysis of body water, fat and lean muscle mass (Body Stat 1500 MDD)	☐	☐	☐
Anthropometry	Height (stadiometer) and Weight (digital scales); Waist circumference	☐	☐	☐
Physical activity				
Physical activity	Recent Physical Activity Questionnaire (RPAQ)[59]	☐	☐	☐
Cognitive function				
Cognitive function	Catch-Cog,[60] Trail Making Test A and B[61] and Delayed Recall Test[62]	☐	☐	☐
Physical strength and function				
Functional capacity	Short Physical Performance Battery (SPPB) [63]	☐	☐	☐
Muscle strength	Hand Grip Strength (Dynamometer)	☐	☐	☐
Psychosocial				
Health-Related Quality of Life	SF-36 Health Survey[64] questionnaire	☐	☐	☐
Depressive symptoms	Geriatric Depression Scale[65] questionnaire	☐	☐	☐
Blood Pressure				
Blood Pressure	Clinic measured blood pressure (Omron Healthcare)	☐	☐	☐
Biochemical Measures				
Blood biomarkers	Nutritional: vitamins A,E, and carotenoids.<br>cardiometabolic: lipid profile, HbA1c ,glucose and insulin.<br>Inflammatory: hs-CRP, IL-6.<br>Metabolomic analysis	☐	☐	☐
**Evaluation**				
Study evaluation	Questionnaire			☐

For peer review only - http://bmjopen.bmj.com/site/about/guidelines.xhtml

Primary outcome

The primary study outcome is between-group difference in change in nutritional status at 6-months using the validated MNA score (MNA® 0-30).[57] The MNA is the most widely validated tool for detecting undernutrition risk in older adults[66] and is shown to respond to dietary intervention.[47]

Secondary outcomes

Nutrition and body composition

Change in MNA score will be analysed at midpoint (3 months from baseline).. Dietary behaviour will be reported using the PROMED diet score (range 0-14) adapted from a validated Spanish Mediterranean diet score[67] to incorporate the higher protein food targets and tailored for a Northern European population. Higher scores indicate increased concordance with the PROMED diet. Overall dietary intake will be assessed using a 4-day prospective food diary. Participants will be instructed to record all items of food and beverages consumed over four consecutive days (two weekend days) at home and provide details of the portion size consumed, cooking methods and named brands. Data on self-reported appetite will also be collected using the 4-item Simplified Nutrition Appetite Questionnaire (SNAQ) validated for older community dwelling adults.[58] Lower SNAQ scores indicate poorer appetite. Body composition will be assessed using non-invasive bioelectrical impedance analysis (BodyStat 1500 MDD) to estimate body water, fat and lean muscle mass.[68] Anthropometric measures of weight, height and waist circumference will also be taken using digital scales, a stadiometer and flexible measuring tape, respectively.

Physical activity

Time spent on different intensity levels of physical activity will be self-reported using a validated Recent Physical Activity Questionnaire (RPAQ) across four domains: home, commuting, occupational activity (if appropriate), and leisure activity over the previous 4 weeks.[59]

Cognitive function

A brief neuropsychological test battery (NTB) will be administered by a trained researcher to capture cognitive domains that are shown to be sensitive to progression in early stages of memory decline: global cognition, executive functioning, episodic and working memory, information processing speed and sustained attention.[60] The NTB will include the Catch-Cog assessment[60] comprising (i) three Alzheimer's Disease Assessment Scale–Cognitive subscales (Word Recognition (score range 0-12), Word Recall (score range 0-10), and Orientation (score range 0-8)) to assess episodic memory (ii) the Controlled Oral Word Association Test (number of acceptable words in one minute); Category Fluency Test (number of animals named in one minute); (iii) Digit Symbol Substitution Test (total number of correct symbols in 90 seconds) and (iv) Digit Span Backward (score range 0-14) to assess to assess executive function and working memory. The Trail Making Test (A and B) will be used to assess information processing speed and attention.[61] A delayed recall test of recalled learned words after a 5-minute delay (score range 0–10) will be administered, with higher scores indicating better memory performance. Summary composite scores will be created for global cognition and domains of memory and executive function.

Physical strength and function

For peer review only - http://bmjopen.bmj.com/site/about/guidelines.xhtml

Lower extremity functional status will be measured using the Short Physical Performance Battery (SPPB).[63] The SPPB includes three timed tasks to assess walking speed, chair stand and standing balance, with scores ranging from 0 (worst performance) to 12 (best performance). Muscle (handgrip) strength will be measured with a calibrated dynamometer (Type) (kg) using a standard procedure.[48]

Psychosocial

Health-related Quality of Life (HR QoL) will be determined by using the Short Form-36 (SF-36) 36-item questionnaire[64] which measures eight domains of health status: physical functioning, role limitations due to physical issues, role limitation due to emotional problems, energy/fatigue, emotional well-being, social functioning, pain, and general health perception. SF-36 scores range from 0-100 with higher scores indicating better HR QoL.[64] Depressive symptoms will be determined using the 15-item Geriatric Depression Scale (GDS) – Short Form). GDS scores range from 0 to 15, with a score ≥5 suggesting depression.[65]

Blood pressure

Systolic and diastolic blood pressure (mmHg) will be measured three times in the dominant arm after a 5-minute rest in the seated position using a calibrated automated device approved by the British Hypertension Society (Omron Healthcare). The first measure will be discarded and the average of the final two measurements will be recorded.

Biochemical measures

A 50 ml fasting peripheral venous blood sample will be taken at the start of each study assessment for metabolomic profiling and biomarker measurement. Anonymised samples will be processed in the Nutrition laboratory at QUB according to standardised protocols and stored in aliquots at -80°C until analysis. Biochemical markers relating to nutrition (vitamins A, E, and carotenoids,), metabolic (lipid profile, HbA1c, glucose and insulin), and inflammation (hs-CRP, IL-6) will be measured. Plasma samples will be transferred to University College Dublin for metabolomics analysis.

Evaluation

A questionnaire will be administered at the end of the study to determine acceptability and tolerance of the intervention and the overall experience of taking part in the study. Participants will be asked to return diet and exercise logs to evaluate engagement with the intervention.

Data management and statistical analysis plan

Data will be de-identified and stored securely according to General Data Protection Regulation (GDPR) and UK Data Protection Act 2018 in QUB. There are no plans for interim study analysis. The groups will be similar due to randomisation therefore there are no a priori plans to include covariates in the analyses. Participant characteristics by allocated group will be compared using analysis of variance for continuous variables and Chi square test for dichotomous variables. Skewed variables will be log transformed prior to analysis. An intention-to-treat analysis will be conducted using multiple linear regression model to compare MNA score at 6 months between the three groups adjusting for baseline. Dunnett's procedure will then be used to compare each intervention group to the controls, accounting

For peer review only - http://bmjopen.bmj.com/site/about/guidelines.xhtml

for multiple testing. Analysis of secondary outcomes will utilise a similar method and these exploratory analyses will be interpreted cautiously. Sensitivity analyses will be conducted to evaluate the diet and exercise behavioural changes maintained over 6-months and to determine the effect of intervention compliance on study-end-points. Multiple imputations for missing data will be used if required. Analyses will be performed using STATA Statistical Software: StataCorp LLC.

Ethics and dissemination

Ethical approval has been received from Office for Research Ethics Committee (OREC) (Ref 21/NW/0215) and the trial has been registered on ClinicalTrials.gov (NCT 05166564). The study will be reported according to CONSORT guidelines for parallel group randomised trials.[69] Written informed consent will be obtained from all study participants prior to study enrolment and any adverse events will be reported to the study sponsor (QUB) and OREC.

Study findings will be of interest to a range of stakeholders in the areas of healthy ageing, preventable malnutrition, cognitive decline, and dementia risk reduction, including academics, the public, healthcare professionals and clinicians, charitable organisations, and industry. Findings will be published in academic journals and published in open access repositories, for example the JPI-HDHL Meta Database (http://www.jpihdhl.eu) knowledge transfer platform.

COVID-19

The research team will comply with public health and QUB guidance regarding COVID-19. Several strategies will be implemented to mitigate risk: (i) COVID-19 risk screening for participants prior to study visits (ii) optional home study visits, (iii) optional remote delivery of intervention support, (iv) personal protective equipment for researchers conducting study assessments, (v) participant face coverings for study visits, (vi) social distancing measures followed where possible, and (vii) researcher lateral flow testing pre-study visit.

DISCUSSION

To our knowledge, PROMED-EX is the first study aimed to evaluate the combined effects of a protein-enriched MedDiet and exercise on nutritional status and cognition in older adults at risk of undernutrition with SCD. Lifestyle interventions to address modifiable risk factors for dementia are likely to have a more significant public health impact when implemented at an early stage of disease and in high-risk populations. PROMED-EX will generate new knowledge about how a targeted lifestyle intervention delivered in a ‘real-world’ setting can impact nutritional and cognitive status among older individuals at risk of undernutrition and cognitive decline. A further novel aspect is the planned evaluation that will provide insight into lifestyle behaviour changes that are feasible and acceptable for the intended population.

A key strength is the inclusion of a validated neurocognitive test battery in the current study allowing a comprehensive assessment of performance in both memory and non-memory domains of cognition as used in other multidomain interventions in adults at increased dementia risk [42]. Prior studies in undernourished patients have relied on the limited MMSE global cognition to measure cognitive performance.[25] While MMSE is a validated screener for cognitive impairment, it provides limited examination of visuospatial cognitive ability,

For peer review only - http://bmjopen.bmj.com/site/about/guidelines.xhtml

long-term memory, and language functions. It also has a "ceiling" effect whereby individuals can still score quite highly even though they may have significant cognitive impairment.[70]

A further strength is the integration of state-of-the-art metabolomics, metabolic, inflammatory, and nutritional biomarkers in the study will help to elucidate biological mechanisms underlying how diet can influence nutrition status and cognition, and how exercise may act to modify these processes. Furthermore, the interventions will be delivered using a personalised approach with incorporation of evidence-based techniques to support and encourage behaviour change. Study participants will also benefit from written educational resources that have been co-designed with PPI members to support the desired diet and exercise behaviour changes. The ongoing collaboration with PPI members in the trial will undoubtedly optimise the dissemination of study findings.

There are some limitations to the study design that should be considered. The optimum period for lifestyle-induced change in nutritional status is not clear, and the duration of the current study intervention is shorter than in other multidomain trials;[42] however, improvement in MNA score in response to nutrition intervention has been observed in 12 weeks.[63] Furthermore, 6 months should be sufficient duration to detect increases in nutrient biomarkers in response to the interventions, especially in a high-risk population.

The pragmatic study design means that we may have limited power to detect between intervention group effects on MNA. Furthermore, due to the novelty of the intervention and population, it was not possible to estimate sample size calculation for cognition, however the findings generated from PROMED-EX on cognitive performance will be used to inform future studies.

The level of research support for lifestyle behaviour change differs between the intervention and control groups where only the active intervention groups will receive weekly contact for the first 3 months to help meet intervention targets. As a result, intervention completers could be more motivated than control participants which may confound results. Lastly, consistent with other lifestyle interventions participants will not be blinded to treatment allocation, however, data will be analysed by a blinded member of the study team to minimise detection bias.

Conclusion

PROMED-EX focuses on undernutrition and cognitive impairment which are major contributors to mortality and disability in older age. The findings will provide new and critical insight into how modifications of diet and exercise behaviour can act to prevent undernutrition in older citizens at risk of undernutrition and cognitive decline. The study will advance understanding of how the protein enriched MedDiet influences nutritional status and cognitive performance, and whether biological pathways are altered by the addition of exercise. We expect the study findings to inform future dietary guidelines for prevention of undernutrition to promote healthy neurocognitive ageing, and guide the design of future larger trials to test the efficacy of diet and exercise on cognition.

Trial status:

Recruitment began in January 2022 and will complete August 2023. Data collection is scheduled to complete in January 2024.

For peer review only - http://bmjopen.bmj.com/site/about/guidelines.xhtml

Contributions:

CME, LB, CC, FP, LdG, BMG, MMK, JVW, DV contributed to the conception and design of the trial. CME and NW drafted the manuscript. All authors (CME, NW, RRM, LB, CC, LdG, SM, NMC, BMG, MMK, MN, RON, FP, GS, CT, DV, JVW) contributed to the editing of manuscript and revising it critically for intellectual content. The Trial Steering Committee approved this manuscript submission. CME gave final manuscript approval and is the corresponding and senior author.

Funding

Study Sponsor: Queen's University Belfast (Ref: B21/16)

Grant reference: BB/V019201/1

Acknowledgements: The authors would like to thank the study participants, primary care and other community colleagues that are assisting with recruitment. We are also grateful to our PPI and TSC members for their support in the study. We also acknowledge the European Joint Programming Initiative "A Healthy Diet for a Healthy Life" (JPI HDHL) and of the ERA NET Cofund ERA HDHL (GA N 696295 of the EU Horizon 2020 Research and Innovation Programme) for funding the overall 'PROMEDCOG consortium project' including the national funding bodies: The UK Research & Innovation (UKRI) Biotechnology and Biological Sciences Research Council and Medical Research Council, the Italian Ministry of Universities and Research and the Health Research Board Ireland.

Competing interests: none declared

Protocol Version 3: Any amendments will be reviewed and approved by Trial Steering Committee and OREC prior to implementation.

REFERENCES

For peer review only - http://bmjopen.bmj.com/site/about/guidelines.xhtml

1. Cederholm T, Bosaeus I, Barazzoni R, et al. Diagnostic criteria for malnutrition – An ESPEN Consensus Statement. Clinical Nutrition 2015;34(3):335-40. doi: 10.1016/j.clnu.2015.03.001
2. Stratton RJ Smith T, Gabe S. Managing malnutrition to improve lives and save money. BAPEN, 2018.
3. Sanders CL, Wengreen HJ, Schwartz S, et al. Nutritional Status is Associated With Severe Dementia and Mortality: The Cache County Dementia Progression Study. Alzheimer Dis Assoc Disord 2018;32(4):298-304. doi: 10.1097/wad.0000000000000274
4. Guerin O, Soto ME, Brocker P, et al. Nutritional status assessment during Alzheimer's disease: results after one year (the REAL French Study Group). J Nutr Health Aging 2005;9(2):81-4. PMID: 15791350.
5. Qizilbash N, Gregson J, Johnson ME, et al. BMI and risk of dementia in two million people over two decades: a retrospective cohort study. Lancet Diabetes Endocrinol 2015;3(6):431-36. doi: 10.1016/s2213-8587(15)00033-9 [published Online First: 20150409]
6. Lee CM, Woodward M, Batty GD, et al. Association of anthropometry and weight change with risk of dementia and its major subtypes: A meta-analysis consisting 2.8 million adults with 57 294 cases of dementia. Obes Rev 2020;21(4):e12989. doi: 10.1111/obr.12989 [published Online First: 20200103]
7. Knopman DS, Edland SD, Cha RH, et al. Incident dementia in women is preceded by weight loss by at least a decade. Neurology 2007;69(8):739-46. doi: 10.1212/01.wnl.0000267661.65586.33
8. LeBlanc ES, Rizzo JH, Pedula KL, et al. Weight Trajectory over 20 Years and Likelihood of Mild Cognitive Impairment or Dementia Among Older Women. J Am Geriatr Soc 2017;65(3):511-19. doi: 10.1111/jgs.14552 [published Online First: 20161219]
9. Volkert D, Kiesswetter E, Cederholm T, et al. Development of a Model on Determinants of Malnutrition in Aged Persons: A MaNuEL Project. Gerontol Geriatr Med 2019;5:2333721419858438-38. doi: 10.1177/2333721419858438
10. Burns JM, Johnson DK, Watts A, et al. Reduced lean mass in early Alzheimer disease and its association with brain atrophy. Arch Neurol 2010;67(4):428-33. doi: 10.1001/archneurol.2010.38
11. Yu JH, Kim REY, Jung JM, et al. Sarcopenia is associated with decreased gray matter volume in the parietal lobe: a longitudinal cohort study. BMC Geriatr 2021;21(1):622. doi: 10.1186/s12877-021-02581-4 [published Online First: 20211102]
12. Jimenez A, Pegueroles J, Carmona-Iragui M, et al. Weight loss in the healthy elderly might be a non-cognitive sign of preclinical Alzheimer's disease. Oncotarget 2017;8(62):104706-16. doi: 10.18632/oncotarget.22218 [published Online First: 20171031]
13. McGrattan AM, McGuinness B, McKinley MC, et al. Diet and Inflammation in Cognitive Ageing and Alzheimer's Disease. Curr Nutr Rep 2019;8(2):53-65. doi: 10.1007/s13668-019-0271-4
14. Ahima RS, Antwi DA. Brain regulation of appetite and satiety. Endocrinol Metab Clin North Am 2008;37(4):811-23. doi: 10.1016/j.ecl.2008.08.005
15. Kimura A, Sugimoto T, Niida S, et al. Association Between Appetite and Sarcopenia in Patients With Mild Cognitive Impairment and Early-Stage Alzheimer's Disease: A Case-Control Study. Front Nutr 2018;5:128. doi: 10.3389/fnut.2018.00128 [published Online First: 20181218]
16. Attems J, Walker L, Jellinger KA. Olfaction and Aging: A Mini-Review. Gerontology 2015;61(6):485-90. doi: 10.1159/000381619 [published Online First: 20150509]
17. Doorduijn AS, van de Rest O, van der Flier WM, et al. Energy and Protein Intake of Alzheimer's Disease Patients Compared to Cognitively Normal Controls: Systematic Review.

For peer review only

For peer review only - http://bmjopen.bmj.com/site/about/guidelines.xhtml

J Am Med Dir Assoc 2019;20(1):14-21. doi: 10.1016/j.jamda.2018.06.019 [published Online First: 20180809]
18. Alagiakrishnan K, Bhanji RA, Kurian M. Evaluation and management of oropharyngeal dysphagia in different types of dementia: A systematic review. Archives of Gerontology and Geriatrics 2013;56(1):1-9. doi: https://doi.org/10.1016/j.archger.2012.04.011
19. Hiller AJ, Ishii M. Disorders of Body Weight, Sleep and Circadian Rhythm as Manifestations of Hypothalamic Dysfunction in Alzheimer's Disease. Front Cell Neurosci 2018;12:471. doi: 10.3389/fncel.2018.00471 [published Online First: 20181205]
20. Honda Y, Meguro K, Meguro M, et al. Social withdrawal of persons with vascular dementia associated with disturbance of basic daily activities, apathy, and impaired social judgment. Care Manag J 2013;14(2):108-13. doi: 10.1891/1521-0987.14.2.108
21. Volkert D, Chourdakis M, Faxen-Irving G, et al. ESPEN guidelines on nutrition in dementia. Clin Nutr 2015;34(6):1052-73. doi: 10.1016/j.clnu.2015.09.004 [published Online First: 20150925]
22. Reinders I, Wijnhoven HAH, Jyväkorpi SK, et al. Effectiveness and cost-effectiveness of personalised dietary advice aiming at increasing protein intake on physical functioning in community-dwelling older adults with lower habitual protein intake: rationale and design of the PROMISS randomised controlled trial. BMJ Open 2020;10(11):e040637. doi: 10.1136/bmjopen-2020-040637 [published Online First: 20201120]
23. Endevelt R, Lemberger J, Bregman J, et al. Intensive dietary intervention by a dietitian as a case manager among community dwelling older adults: the EDIT study. J Nutr Health Aging 2011;15(8):624-30. doi: 10.1007/s12603-011-0074-9
24. Wouters-Wesseling W, Vos AP, Van Hal M, et al. The effect of supplementation with an enriched drink on indices of immune function in frail elderly. J Nutr Health Aging 2005;9(4):281-6. PMID: 15980931.
25. Blondal BS, Geirsdottir OG, Halldorsson TI, et al. HOMEFOOD randomised trial – Six-month nutrition therapy improves quality of life, self-rated health, cognitive function, and depression in older adults after hospital discharge. Clinical Nutrition ESPEN 2022;48:74-81. doi: https://doi.org/10.1016/j.clnesp.2022.01.010
26. Casas R, Sacanella E, Urpí-Sardà M, et al. Long-Term Immunomodulatory Effects of a Mediterranean Diet in Adults at High Risk of Cardiovascular Disease in the PREvención con DIeta MEDiterránea (PREDIMED) Randomized Controlled Trial. J Nutr 2016;146(9):1684-93. doi: 10.3945/jn.115.229476 [published Online First: 20160720]
27. Berti V, Walters M, Sterling J, et al. Mediterranean diet and 3-year Alzheimer brain biomarker changes in middle-aged adults. Neurology 2018;90(20):e1789-e98. doi: 10.1212/wnl.0000000000005527 [published Online First: 20180413]
28. McEvoy CT, Hoang T, Sidney S, et al. Dietary patterns during adulthood and cognitive performance in midlife: The CARDIA study. Neurology 2019;92(14):e1589-e99. doi: 10.1212/wnl.0000000000007243 [published Online First: 20190306]
29. van de Rest O, Berendsen AA, Haveman-Nies A, et al. Dietary patterns, cognitive decline, and dementia: a systematic review. Adv Nutr 2015;6(2):154-68. doi: 10.3945/an.114.007617
30. Limongi F, Siviero P, Bozanic A, et al. The Effect of Adherence to the Mediterranean Diet on Late-Life Cognitive Disorders: A Systematic Review. Journal of the American Medical Directors Association 2020;21(10):1402-09. doi: https://doi.org/10.1016/j.jamda.2020.08.020
31. Singh B, Parsaik AK, Mielke MM, et al. Association of mediterranean diet with mild cognitive impairment and Alzheimer's disease: a systematic review and meta-analysis. J Alzheimers Dis 2014;39(2):271-82. doi: 10.3233/jad-130830

For peer review only - http://bmjopen.bmj.com/site/about/guidelines.xhtml

32. Feart C, Samieri C, Barberger-Gateau P. Mediterranean diet and cognitive health: an update of available knowledge. Curr Opin Clin Nutr Metab Care 2015;18(1):51-62. doi: 10.1097/mco.0000000000000131
33. Castro-Quezada I, Román-Viñas B, Serra-Majem L. The Mediterranean diet and nutritional adequacy: a review. Nutrients 2014;6(1):231-48. doi: 10.3390/nu6010231
34. Kiesswetter E, Sieber CC, Volkert D. Protein intake in older people : Why, how much and how? Z Gerontol Geriatr 2020;53(4):285-89. doi: 10.1007/s00391-020-01723-4 [published Online First: 20200414]
35. Volkert D, Beck AM, Cederholm T, et al. ESPEN guideline on clinical nutrition and hydration in geriatrics. Clinical Nutrition 2019;38(1):10-47. doi: https://doi.org/10.1016/j.clnu.2018.05.024
36. Okamura T, Miki A, Hashimoto Y, et al. Shortage of energy intake rather than protein intake is associated with sarcopenia in elderly patients with type 2 diabetes: A cross-sectional study of the KAMOGAWA-DM cohort. J Diabetes 2019;11(6):477-83. doi: 10.1111/1753-0407.12874 [published Online First: 20181204]
37. Bauer J, Biolo G, Cederholm T, et al. Evidence-based recommendations for optimal dietary protein intake in older people: a position paper from the PROT-AGE Study Group. J Am Med Dir Assoc 2013;14(8):542-59. doi: 10.1016/j.jamda.2013.05.021 [published Online First: 20130716]
38. Granic A, Mendonça N, Sayer AA, et al. Effects of dietary patterns and low protein intake on sarcopenia risk in the very old: The Newcastle 85+ study. Clin Nutr 2020;39(1):166-73. doi: 10.1016/j.clnu.2019.01.009 [published Online First: 20190121]
39. Livingston G, Sommerlad A, Orgeta V, et al. Dementia prevention, intervention, and care. Lancet 2017;390(10113):2673-734. doi: 10.1016/s0140-6736(17)31363-6 [published Online First: 20170720]
40. Kirk-Sanchez NJ, McGough EL. Physical exercise and cognitive performance in the elderly: current perspectives. Clin Interv Aging 2014;9:51-62. doi: 10.2147/cia.S39506 [published Online First: 20131218]
41. Lista I, Sorrentino G. Biological Mechanisms of Physical Activity in Preventing Cognitive Decline. Cellular and Molecular Neurobiology 2010;30(4):493-503. doi: 10.1007/s10571-009-9488-x
42. Ngandu T, Lehtisalo J, Solomon A, et al. A 2 year multidomain intervention of diet, exercise, cognitive training, and vascular risk monitoring versus control to prevent cognitive decline in at-risk elderly people (FINGER): a randomised controlled trial. Lancet 2015;385(9984):2255-63. doi: 10.1016/s0140-6736(15)60461-5 [published Online First: 20150312]
43. Trommelen J, Betz MW, van Loon LJC. The Muscle Protein Synthetic Response to Meal Ingestion Following Resistance-Type Exercise. Sports Med 2019;49(2):185-97. doi: 10.1007/s40279-019-01053-5
44. McGlory C, van Vliet S, Stokes T, et al. The impact of exercise and nutrition on the regulation of skeletal muscle mass. J Physiol 2019;597(5):1251-58. doi: 10.1113/jp275443 [published Online First: 20180801]
45. O'Neill R, Brennan L, Prinelli F, et al. PROtein enriched MEDiterranean diet to combat undernutrition and promote healthy neuroCOGnitive ageing in older adults: The PROMED-COG consortium project. Nutrition Bulletin 2022 doi: 10.1111/nbu.12571
46. Chan A-W, Tetzlaff JM, Gøtzsche PC, et al. SPIRIT 2013 explanation and elaboration: guidance for protocols of clinical trials. BMJ : British Medical Journal 2013;346:e7586. doi: 10.1136/bmj.e7586
47. Luger E, Dorner TE, Haider S, et al. Effects of a Home-Based and Volunteer-Administered Physical Training, Nutritional, and Social Support Program on Malnutrition

For peer review only - http://bmjopen.bmj.com/site/about/guidelines.xhtml

and Frailty in Older Persons: A Randomized Controlled Trial. J Am Med Dir Assoc 2016;17(7):671.e9-71.e16. doi: 10.1016/j.jamda.2016.04.018
48. Marseglia A, Xu W, Fratiglioni L, et al. Effect of the NU-AGE Diet on Cognitive Functioning in Older Adults: A Randomized Controlled Trial. Front Physiol 2018;9:349-49. doi: 10.3389/fphys.2018.00349
49. McEvoy CT, Moore SE, Appleton KM, et al. Trial to Encourage Adoption and Maintenance of a Mediterranean Diet (TEAM-MED): Protocol for a Randomised Feasibility Trial of a Peer Support Intervention for Dietary Behaviour Change in Adults at High Cardiovascular Disease Risk. Int J Environ Res Public Health 2018;15(6) doi: 10.3390/ijerph15061130 [published Online First: 20180531]
50. van de Rest O, van der Zwaluw NL, Tieland M, et al. Effect of resistance-type exercise training with or without protein supplementation on cognitive functioning in frail and pre-frail elderly: secondary analysis of a randomized, double-blind, placebo-controlled trial. Mech Ageing Dev 2014;136-137:85-93. doi: 10.1016/j.mad.2013.12.005 [published Online First: 20131227]
51. Kaiser MJ, Bauer JM, Ramsch C, et al. Validation of the Mini Nutritional Assessment short-form (MNA®-SF): A practical tool for identification of nutritional status. JNHA - The Journal of Nutrition, Health and Aging 2009;13(9):782. doi: 10.1007/s12603-009-0214-7
52. CDC. BRFSS Questionnaires. July ed: Centres for Disease Control and Prevention, 2020.
53. Excellence NNIfHaC. Nutrition support for adults: oral nutrition support, enteral tube feeding and parenteral nutrition. Clinical guideline CG32. ONLINE, 2017.
54. Skelly F. Community-based exercise rehabilitation in a diverse chronic disease population. PhD thesis, Dublin City University, 2021.
55. Lea JWD, O'Driscoll JM, Hulbert S, et al. Convergent Validity of Ratings of Perceived Exertion During Resistance Exercise in Healthy Participants: A Systematic Review and Meta-Analysis. Sports Medicine - Open 2022;8(1):2. doi: 10.1186/s40798-021-00386-8
56. Lin H, Xu D, Yang M, et al. Behaviour change techniques that constitute effective planning interventions to improve physical activity and diet behaviour for people with chronic conditions: a systematic review. BMJ Open 2022;12(8):e058229. doi: 10.1136/bmjopen-2021-058229 [published Online First: 20220822]
57. Guigoz Y, Vellas B. Nutritional Assessment in Older Adults : MNA® 25 years of a Screening Tool and a Reference Standard for Care and Research; What Next? J Nutr Health Aging 2021;25(4):528-83. doi: 10.1007/s12603-021-1601-y
58. Wilson MM, Thomas DR, Rubenstein LZ, et al. Appetite assessment: simple appetite questionnaire predicts weight loss in community-dwelling adults and nursing home residents. Am J Clin Nutr 2005;82(5):1074-81. doi: 10.1093/ajcn/82.5.1074
59. Besson H, Brage S, Jakes RW, et al. Estimating physical activity energy expenditure, sedentary time, and physical activity intensity by self-report in adults. Am J Clin Nutr 2010;91(1):106-14. doi: 10.3945/ajcn.2009.28432 [published Online First: 20091104]
60. Jutten RJ, Harrison JE, Brunner AJ, et al. The Cognitive-Functional Composite is sensitive to clinical progression in early dementia: Longitudinal findings from the Catch-Cog study cohort. Alzheimers Dement (N Y) 2020;6(1):e12020. doi: 10.1002/trc2.12020 [published Online First: 20200417]
61. Lezak MD, Howieson DB, Loring DW. Neuropsychological Assessment. 4th ed: New York: Oxford University Press 2004.
62. Zhao Q, Lv Y, Zhou Y, et al. Short-term delayed recall of auditory verbal learning test is equivalent to long-term delayed recall for identifying amnestic mild cognitive impairment. PLoS One 2012;7(12):e51157. doi: 10.1371/journal.pone.0051157 [published Online First: 20121207]

For peer review only - http://bmjopen.bmj.com/site/about/guidelines.xhtml

63. Guralnik JM, Simonsick EM, Ferrucci L, et al. A short physical performance battery assessing lower extremity function: association with self-reported disability and prediction of mortality and nursing home admission. J Gerontol 1994;49(2):M85-94. doi: 10.1093/geronj/49.2.m85
64. Ware JE, Jr., Sherbourne CD. The MOS 36-item short-form health survey (SF-36). I. Conceptual framework and item selection. Med Care 1992;30(6):473-83.
65. Yesavage JA, Brink TL, Rose TL, et al. Development and validation of a geriatric depression screening scale: a preliminary report. J Psychiatr Res 1982;17(1):37-49. doi: 10.1016/0022-3956(82)90033-4
66. Vellas B, Villars H, Abellan G, et al. Overview of the MNA--Its history and challenges. J Nutr Health Aging 2006;10(6):456-63; discussion 63-5. PMID: 17183418.
67. Schröder H, Fitó M, Estruch R, et al. A short screener is valid for assessing Mediterranean diet adherence among older Spanish men and women. J Nutr 2011;141(6):1140-5. doi: 10.3945/jn.110.135566 [published Online First: 20110420]
68. Genton L, Karsegard VL, Kyle UG, et al. Comparison of Four Bioelectrical Impedance Analysis Formulas in Healthy Elderly Subjects. Gerontology 2001;47:315 - 23. PMID: 11721145.
69. Moher D, Hopewell S, Schulz KF, et al. CONSORT 2010 Explanation and Elaboration: Updated guidelines for reporting parallel group randomised trials. J Clin Epidemiol 2010;63(8):e1-37. doi: 10.1016/j.jclinepi.2010.03.004 [published Online First: 20100325]
70. Franco-Marina F, García-González JJ, Wagner-Echeagaray F, et al. The Mini-mental State Examination revisited: ceiling and floor effects after score adjustment for educational level in an aging Mexican population. Int Psychogeriatr 2010;22(1):72-81. doi: 10.1017/s1041610209990822 [published Online First: 20090907]

Figure 1: Overview of PROMED-EX Study Design

For peer review only

For peer review only - http://bmjopen.bmj.com/site/about/guidelines.xhtml

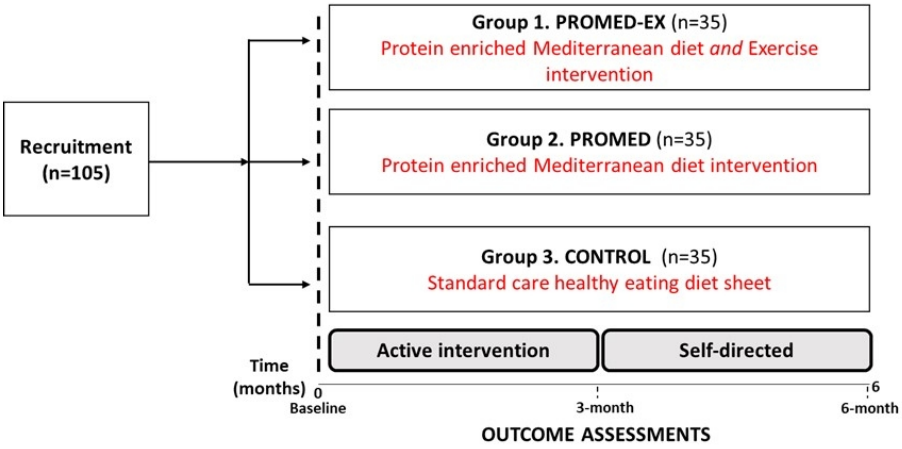

Overview of PROMED-EX Study Design

159x72mm (150 x 150 DPI)

SPIRIT

STANDARD PROTOCOL ITEMS: RECOMMENDATIONS FOR INTERVENTIONAL TRIALS

SPIRIT 2013 Checklist: Recommended items to address in a clinical trial protocol and related documents*

Section/item	Item No	Description	Addressed on page number
**Administrative information**			
Title	1	Descriptive title identifying the study design, population, interventions, and, if applicable, trial acronym	**1**
Trial registration	2a	Trial identifier and registry name. If not yet registered, name of intended registry	**1**
	2b	All items from the World Health Organization Trial Registration Data Set	**1**
Protocol version	3	Date and version identifier	**14**
Funding	4	Sources and types of financial, material, and other support	**14**
Roles and responsibilities	5a	Names, affiliations, and roles of protocol contributors	**1,14**
	5b	Name and contact information for the trial sponsor	**1**
	5c	Role of study sponsor and funders, if any, in study design; collection, management, analysis, and interpretation of data; writing of the report; and the decision to submit the report for publication, including whether they will have ultimate authority over any of these activities	**14**
	5d	Composition, roles, and responsibilities of the coordinating centre, steering committee, endpoint adjudication committee, data management team, and other individuals or groups overseeing the trial, if applicable (see Item 21a for data monitoring committee)	**Appendix 2**

For peer review only

Introduction

Background and rationale	6a	Description of research question and justification for undertaking the trial, including summary of relevant studies (published and unpublished) examining benefits and harms for each intervention	**3,4**
	6b	Explanation for choice of comparators	**4**
Objectives	7	Specific objectives or hypotheses	**4**
Trial design	8	Description of trial design including type of trial (eg, parallel group, crossover, factorial, single group), allocation ratio, and framework (eg, superiority, equivalence, noninferiority, exploratory)	**4**

Methods: Participants, interventions, and outcomes

Study setting	9	Description of study settings (eg, community clinic, academic hospital) and list of countries where data will be collected. Reference to where list of study sites can be obtained	**5**
Eligibility criteria	10	Inclusion and exclusion criteria for participants. If applicable, eligibility criteria for study centres and individuals who will perform the interventions (eg, surgeons, psychotherapists)	**5,6**
Interventions	11a	Interventions for each group with sufficient detail to allow replication, including how and when they will be administered	**6-8**
	11b	Criteria for discontinuing or modifying allocated interventions for a given trial participant (eg, drug dose change in response to harms, participant request, or improving/worsening disease)	**N/A**
	11c	Strategies to improve adherence to intervention protocols, and any procedures for monitoring adherence (eg, drug tablet return, laboratory tests)	**8**
	11d	Relevant concomitant care and interventions that are permitted or prohibited during the trial	**6-8**
Outcomes	12	Primary, secondary, and other outcomes, including the specific measurement variable (eg, systolic blood pressure), analysis metric (eg, change from baseline, final value, time to event), method of aggregation (eg, median, proportion), and time point for each outcome. Explanation of the clinical relevance of chosen efficacy and harm outcomes is strongly recommended	**10,11**
Participant timeline	13	Time schedule of enrolment, interventions (including any run-ins and washouts), assessments, and visits for participants. A schematic diagram is highly recommended (see Figure)	**5 (Figure 1)**

For peer review only - http://bmjopen.bmj.com/site/about/guidelines.xhtml

Sample size	14	Estimated number of participants needed to achieve study objectives and how it was determined, including clinical and statistical assumptions supporting any sample size calculations	**11**
Recruitment	15	Strategies for achieving adequate participant enrolment to reach target sample size	**5**
**Methods: Assignment of interventions (for controlled trials)**			
Allocation:			
Sequence generation	16a	Method of generating the allocation sequence (eg, computer-generated random numbers), and list of any factors for stratification. To reduce predictability of a random sequence, details of any planned restriction (eg, blocking) should be provided in a separate document that is unavailable to those who enrol participants or assign interventions	**6**
Allocation concealment mechanism	16b	Mechanism of implementing the allocation sequence (eg, central telephone; sequentially numbered, opaque, sealed envelopes), describing any steps to conceal the sequence until interventions are assigned	**6**
Implementation	16c	Who will generate the allocation sequence, who will enrol participants, and who will assign participants to interventions	**6**
Blinding (masking)	17a	Who will be blinded after assignment to interventions (eg, trial participants, care providers, outcome assessors, data analysts), and how	**6**
	17b	If blinded, circumstances under which unblinding is permissible, and procedure for revealing a participant's allocated intervention during the trial	**N/A**
**Methods: Data collection, management, and analysis**			
Data collection methods	18a	Plans for assessment and collection of outcome, baseline, and other trial data, including any related processes to promote data quality (eg, duplicate measurements, training of assessors) and a description of study instruments (eg, questionnaires, laboratory tests) along with their reliability and validity, if known. Reference to where data collection forms can be found, if not in the protocol	**8-11**
	18b	Plans to promote participant retention and complete follow-up, including list of any outcome data to be collected for participants who discontinue or deviate from intervention protocols	**11**

Data management	19	Plans for data entry, coding, security, and storage, including any related processes to promote data quality (eg, double data entry; range checks for data values). Reference to where details of data management procedures can be found, if not in the protocol	**12**
Statistical methods	20a	Statistical methods for analysing primary and secondary outcomes. Reference to where other details of the statistical analysis plan can be found, if not in the protocol	**11**
	20b	Methods for any additional analyses (eg, subgroup and adjusted analyses)	**N/A**
	20c	Definition of analysis population relating to protocol non-adherence (eg, as randomised analysis), and any statistical methods to handle missing data (eg, multiple imputation)	**12**
**Methods: Monitoring**			
Data monitoring	21a	Composition of data monitoring committee (DMC); summary of its role and reporting structure; statement of whether it is independent from the sponsor and competing interests; and reference to where further details about its charter can be found, if not in the protocol. Alternatively, an explanation of why a DMC is not needed	**N/A -Appendix 1**
	21b	Description of any interim analyses and stopping guidelines, including who will have access to these interim results and make the final decision to terminate the trial	**N/A (no interim analysis)**
Harms	22	Plans for collecting, assessing, reporting, and managing solicited and spontaneously reported adverse events and other unintended effects of trial interventions or trial conduct	**11**
Auditing	23	Frequency and procedures for auditing trial conduct, if any, and whether the process will be independent from investigators and the sponsor	**None**
**Ethics and dissemination**			
Research ethics approval	24	Plans for seeking research ethics committee/institutional review board (REC/IRB) approval	**12**
Protocol amendments	25	Plans for communicating important protocol modifications (eg, changes to eligibility criteria, outcomes, analyses) to relevant parties (eg, investigators, REC/IRBs, trial participants, trial registries, journals, regulators)	**14**

For peer review only - http://bmjopen.bmj.com/site/about/guidelines.xhtml

Consent or assent	26a	Who will obtain informed consent or assent from potential trial participants or authorised surrogates, and how (see Item 32)	**6, Appendix 4**
	26b	Additional consent provisions for collection and use of participant data and biological specimens in ancillary studies, if applicable	**Appendix 4**
Confidentiality	27	How personal information about potential and enrolled participants will be collected, shared, and maintained in order to protect confidentiality before, during, and after the trial	**12**
Declaration of interests	28	Financial and other competing interests for principal investigators for the overall trial and each study site	**14**
Access to data	29	Statement of who will have access to the final trial dataset, and disclosure of contractual agreements that limit such access for investigators	**12**
Ancillary and post-trial care	30	Provisions, if any, for ancillary and post-trial care, and for compensation to those who suffer harm from trial participation	**N/A**
Dissemination policy	31a	Plans for investigators and sponsor to communicate trial results to participants, healthcare professionals, the public, and other relevant groups (eg, via publication, reporting in results databases, or other data sharing arrangements), including any publication restrictions	**12**
	31b	Authorship eligibility guidelines and any intended use of professional writers	**14**
	31c	Plans, if any, for granting public access to the full protocol, participant-level dataset, and statistical code	**12**
**Appendices**			
Informed consent materials	32	Model consent form and other related documentation given to participants and authorised surrogates	**Appendix 4**
Biological specimens	33	Plans for collection, laboratory evaluation, and storage of biological specimens for genetic or molecular analysis in the current trial and for future use in ancillary studies, if applicable	**Not provided**

*It is strongly recommended that this checklist be read in conjunction with the SPIRIT 2013 Explanation & Elaboration for important clarification on the items. Amendments to the protocol should be tracked and dated. The SPIRIT checklist is copyrighted by the SPIRIT Group under the Creative Commons "Attribution-NonCommercial-NoDerivs 3.0 Unported" license.

Appendix 2- PROMED-EX Trial Steering Committee (TSC)

Table S1: TSC members and their roles

Member	Role
**Prof Emma Stevenson**	Independent Chair
**Prof Jayne Woodside**	Dependant- scientific advisor
**Prof Dorothee Volkert**	Dependant- scientific advisor
**Prof Lisette de Groot**	Dependant- scientific advisor
**Prof Lorraine Brennan**	Dependant- scientific advisor
**Prof Chris Cardwell**	Dependant- statistician
**Ms Bernie Devlin**	Independent lay member
**Ms Eleanor Butterwick**	Independent lay member
**Ms Clare Jess**	Facilitator
**Dr Roisin O'Neill/ Dr Dominic Farsi**	Dependant- Trial manager
**Dr Claire McEvoy**	Dependant- PROMED-EX principal investigator

Table S2: Roles and responsibilities of the TSC

**Roles and responsibilities**	
Aims of the TSC	To act as the oversight body for PROMED-EX on behalf of the Sponsor/Funder.
Terms of reference	The role of the TSC is to provide oversight for the trial [Medical Research Council (MRC) Good Clinical Practice (GCP) guidelines (1998)]. It should also provide advice through its independent Chairperson to the PI, local trial team, BBSRC on all aspects of the trial.
Specific roles of TSC	• provide expert oversight of the trial<br>• maintain confidentiality of all trial information that is not already in the public domain<br>• make decisions as to the future continuation (or otherwise) of the trial/s<br>• monitor recruitment rates and encourage the trial team to develop strategies to deal with any recruitment problems<br>• review regular reports of the trial from the trial team.<br>• assess the impact and relevance of any accumulating external evidence<br>• monitor completion of CRFs and comment on strategies from trial team to encourage satisfactory completion in the future<br>• monitor follow-up rates and review strategies from Trial team to deal with problems<br>• approve any amendments to the protocol, where appropriate<br>• approve any proposals by the Trial team concerning any change to the design of the trial, including additional substudies<br>• oversee the timely reporting of trial results<br>• comment on the main trial manuscript<br>• comment on any abstracts and presentations of any results *during* the running of the trial

For peer review only

Study no ____________ Date____________

PROMED DIET SCORE

Visit: Baseline ☐ 3 mon ☐ 6 mon ☐

Questions	Self-reported	Criteria for 1 point	Point?
1. Do you use olive oil or rapeseed oil as your main cooking fat?		Yes	
2. How much olive/ rapeseed oil do you consume in a given day? Including oil used for frying, salads, out-of-house meals etc (in tablespoons)<br><br>How much olive or rapeseed oil-based spread do you consume in a given day? (in teaspoons)		≥1 tbsp oil / day<br><br>**and/or**<br><br>3 tsp spread/ day	
3. How many servings of dairy foods (milk, cheese, yogurts) or plant proteins alternatives do you eat per day?		≥2 portions per day	
4. How many portions of fruit (including natural fruit juices) do you consume per day? (1 portion = 1 apple/banana (80g), small glass juice (150ml)		≥2 portions/ day	
5. How many vegetable servings do you consume per day? Including raw/ cooked vegetables, salad but not including potatoes (1 serving: 3 tablespoons/80g)		≥3 portions/ day	
6. How many servings of legumes (peas, beans and lentils including kidney beans, baked beans, chickpeas, red lentils etc) do you consume per week? (1 serving :3 tablespoons/ 80g)		≥3 servings/ week	
7. How many servings of red meat including beef, pork, lamb and minced beef do you consume per week? (1 serving: medium portion/ 100–150 g)		≤2 servings /week	
8. How many servings of processed meat including ham, bacon, sausages, meat pies and other meat products etc.) do you consume per week? (1 serving: medium portion/ 100–150 g)		≤1 serving /week	
9. How many servings of chicken/ turkey do you consume per week? (1 serving: medium portion/ 100-150g)		≥2 servings /week	
10. How many servings of fish (tuna, cod, haddock, salmon, mackerel, herring, and sardines etc, including tinned varieties, excluding crumbed or battered fish) or shellfish do you consume per week? (1 serving: 1 fillet/small fish or 140g)		≥2 servings /week	
11. Do you preferentially consume wholegrain bread and/ or cereal and/ or rice and/ or pasta instead of non-wholegrain (white) varieties?		Yes	
12. How many servings of natural nuts do you consume per week? (1 serving: 1 small handful/ 30 g)		≥3 servings /week	
13. How many times per week do you consume sweet foods (including biscuits, buns, pastries, chocolate, sweets, sweet or carbonated beverages or desserts)?		≤3 times/ week	
14. How often would you consume up to 3 small glasses of wine or equivalent other alcoholic beverages per week? (1 small glass:125ml)		1-3 glasses or equivalent ≥ 3 days/ week	

For peer review only

PROMED-EX STUDY ID:________________

CONSENT FORM

**Title of Project:**	**PROMED-EX**: A Randomised Controlled Trial to evaluate the effect of a protein enriched Mediterranean Diet (MedDiet) and exercise intervention on the nutritional status and cognitive performance of individuals at risk of undernutrition and cognitive decline.
**Chief Investigator:**	Dr Claire McEvoy
**Study Number:**	21/NW/0215

Please initial each box

1. I confirm that I have read, or had read to me, and understand the participant information sheet dated 20/11/21, version 3 for the above study. I have had the opportunity to ask questions and these have been answered fully. ☐

2. I understand that my participation is voluntary and I am free to withdraw at any time, without giving any reason and without my legal rights or medical care being affected. ☐

3. I understand that data collected as part of this study may be looked at by authorised individuals from Queen's University Belfast, the research team or regulatory authorities where it is relevant to my taking part in this research. I give permission for these individuals to have access to this information. ☐

4. I understand the study is being conducted by researchers from Queen's University Belfast and that my personal information will be held securely on University premises and handled in accordance with the Data Protection Act 2018 and General Data Protection Regulations. ☐

For peer review only - http://bmjopen.bmj.com/site/about/guidelines.xhtml

PROMED-EX STUDY ID:________________

5. I agree to the research team informing my General Practitioner (GP) if my blood pressure is higher than normal (≥179/109) or tests completed during the study (specifically the nutritional assessment or the cognitive test scores) suggest I may be malnourished or have a cognitive impairment that may require medical intervention. ☐

6. I understand that the information I provide may be published as part of a report. Confidentiality and anonymity will be maintained and it will not be possible to identify me from any publications. ☐

7. I agree to gift a small amount of the blood that will be transferred from Queen's University Belfast to our collaborators laboratory at University College Dublin for analysis. Samples will be labelled using a unique study ID and they will not contain any identifiable information. ☐

8. I understand that if I withdraw (or lose capacity to consent) during the trial, I agree that data and samples already taken can be retained, but no further samples will be collected. ☐

9. I agree to take part in the above study inclusive of all the procedures mentioned in the participant information sheet. ☐

Optional measurement

10. I agree to gift a small sample of stool at the start and end of the 6 month study. This will be sent for analysis to study collaborators at the Quadram Institute, Norwich, England. Samples will be labelled with a unique study ID and they will not contain any identifiable information. ☐

Regarding future Research Studies (Optional)

11. I give permission for my stored, anonymised blood sample to be analysed in future research studies for indicators of age-related health and nutrition–related factors, or by other research groups. ☐

For peer review only - http://bmjopen.bmj.com/site/about/guidelines.xhtml

PROMED-EX STUDY ID:________________

Regarding future research studies (Cont.)

12. I agree to being contacted and invited to take part in future studies of a similar nature. I understand that I am only agreeing to receive information and I am under no obligation to take part in any future studies. ☐

*If you decide not to consent to being contacted in the future it will not have any influence on your involvement in this particular research study and will not affect any standard of care that you receive.

______________________	______________________	______________________
Name of Participant (please print)	Signature	Date

______________________	______________________	______________________
Name of Person Taking Consent (Please print)	Signature	Date

Contact details for study team

Chief Investigator	Trial Manager
Dr Claire McEvoy<br>Centre for Public Health<br>01.024 Institute of Clinical Sciences, A<br>Queen's University Belfast<br>Grosvenor Road<br>Belfast<br>BT12 6BJ<br>Email: c.mcevoy@qub.ac.uk<br><br>Telephone: 0044 2897 6078	Dr Dominic Farsi<br>Centre for Public Health<br>02.031 Institute of Clinical Sciences, B<br>Queen's University Belfast<br>Grosvenor Road<br>Belfast<br>BT12 6BJ<br>Email: d.farsi@qub.ac.uk<br><br>PROMED-EX Mobile: 07594 966740

PROMED_Consent Form V3. 20.11.2021

For peer review only
